# Sesquiterpenoids and 2-(2-Phenylethyl)chromone Derivatives from the Resinous Heartwood of *Aquilaria sinensis*

**DOI:** 10.1007/s13659-021-00313-0

**Published:** 2021-06-01

**Authors:** Shu-Ya Wei, Dong-Bao Hu, Meng-Yuan Xia, Ji-Feng Luo, Hui Yan, Jing-Hua Yang, Yun-Song Wang, Yue-Hu Wang

**Affiliations:** 1 Key Laboratory of Medicinal Chemistry for Natural Resource, Ministry of Education, School of Chemical Science and Technology, School of Pharmacy, Yunnan University, Kunming, 650091 People’s Republic of China; 2 Key Laboratory of Economic Plants and Biotechnology, Yunnan Key Laboratory for Wild Plant Resources, and State Key Laboratory of Phytochemistry and Plant Resources in West China, Chinese Academy of Sciences, Kunming, 650201 People’s Republic of China; 3 School of Chemical Biology and Environment, Yuxi Normal University, Yuxi, 653100 People’s Republic of China

**Keywords:** Thymelaeaceae, *Aquilaria sinensis*, Sesquiterpenoids, 2-(2-phenylethyl)chromones, Neuroprotective

## Abstract

One novel spirolactone, aquilarisinolide (**1**), three new sesquiterpenoids, (2*R*,4*S*,5*R*,7*R*)-2-hydroxyeremophila-9,11-dien-8-one (**2**), (1*R*,4*S*,5*S*,7*R*,11*R*)-13-hydroxyepidaphnauran-9-en-8-one (**3**), and (4*R*,5*S*,7*R*,8*S*,10*S*,13*R*)-8,13-dihydroxyrotunda-1,11-dien-3-one (**4**), together with 13 known compounds (**5**–**17**) were isolated from the resinous heartwood of *Aquilaria sinensis* (Thymelaeaceae). The structures of the new compounds were elucidated based on the analysis of NMR and MS data and theoretical calculations their ECD spectra. The isolated compounds were evaluated for their protective activities against PC12 cell injury induced by corticosterone (CORT) and 1-methyl-4-phenylpyridine ion (MPP^+^), as well as inhibitory activities against BACE1. Compound **4**, 5,6-dihydroxy-2-(2-phenylethyl)chromone (**5**), daphnauranol B (**7**), 6-methoxy-2-[2-(3-methyoxyphenyl)ethyl]chromone (**10**), isoagarotetrol (**14**), and 1-hydroxy-1,5-diphenylpentan-3-one (**16**) showed significant protective effects on CORT-induced injury in PC12 cells at a concentration of 20 μM (*P* < 0.001). Isoagarotetrol (**14**) showed a significant protective effect on MPP^+^-induced injury in PC12 cells at a concentration of 20 μM (*P* < 0.001), while compound **4** showed a moderate activity (*P* < 0.01). The BACE1-inhibitory activities of all tested compounds were very weak with less than 30% inhibition at a concentration of 20 μM.

**Supplementary Information:**

The online version contains supplementary material available at 10.1007/s13659-021-00313-0.

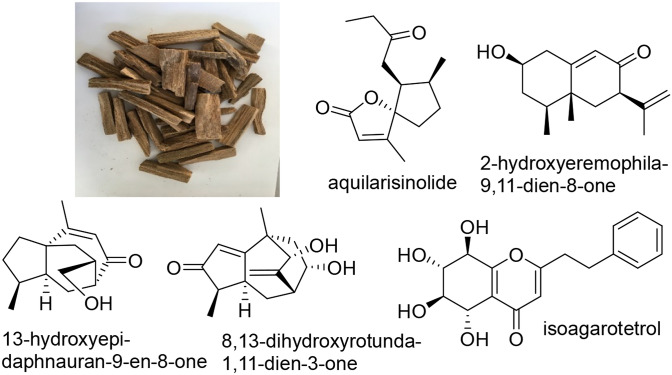

## Introduction

The resinous heartwood of *Aquilaria sinensis* (Lour.) Spreng. (Thymelaeaceae) is known as agarwood (chen-xiang in Chinese). Chen-xiang, a traditional Chinese medicine, is used to treat thoraco-abdominal distension and pain (xiong-fu zhang-men teng-tong), vomiting and hiccups due to stomach cold (wei-han ou-tu e-ni), and asthma due to kidney deficiency (shen-xu qi-ni chuan-ji) [1]. The major chemical constituents from *Aquilaria* plants are sesquiterpenoids and chromones [2–4]. The fractions and components from agarwood and *Aquilaria* trees show various pharmacological activities, such as neural activity, gastrointestinal regulation, cytotoxicity, analgesic effects, and antibacterial, antifungal, anti-inflammatory, antiasthmatic, anti-diabetic, and antioxidant activities [4].

**Fig. 1:**
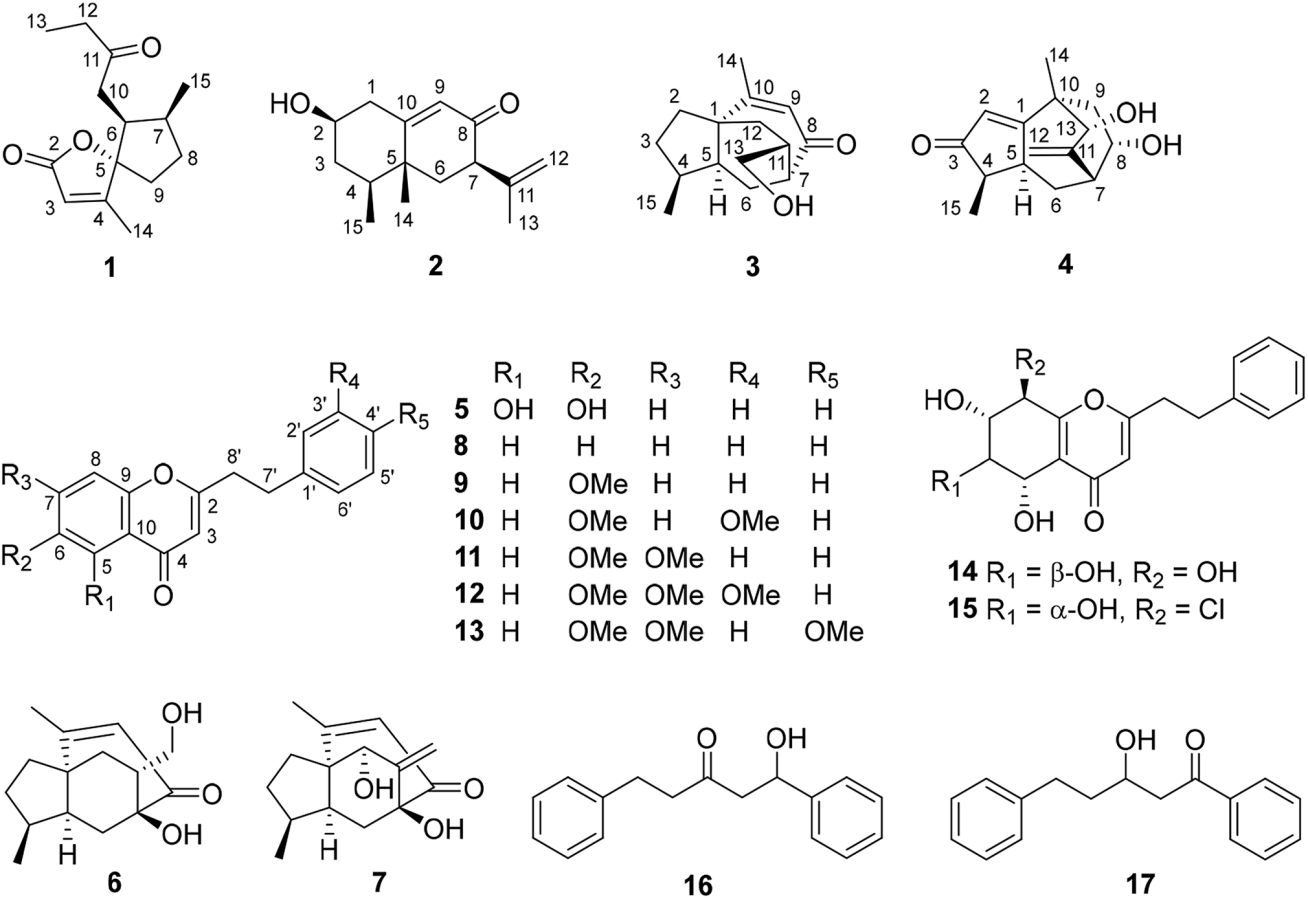
Chemical structures of compounds **1**–**17**

In a previous study, we reported several neuroprotective compounds from the resinous heartwood of *A. sinensis* with the origin in Guangdong, China. One hexahydrochromone and three sesquiterpenoids exert significant protective effects on rat adrenal pheochromocytoma (PC12) cell injury induced by corticosterone (CORT), while the hexahydrochromone and one sesquiterpenoid exhibit significant protective effects on 1-methyl-4-phenylpyridine ion (MPP^+^)-induced PC12 cell injury. All of these compounds from the plant are inactive against beta-site amyloid precursor protein cleaving enzyme 1 (BACE1) [5]. In this paper, the isolation and structural elucidation of 17 compounds (**1**–**17**, Fig. 1), including four new compounds (**1**–**4**), from chen-xiang with the origin in Hainan, China, along with bioassay results in the models of CORT-induced and MPP^+^-induced PC12 cell damage and BACE1 inhibition, are reported.

## Results and Discussion

### Structural Elucidation

**Fig. 2:**
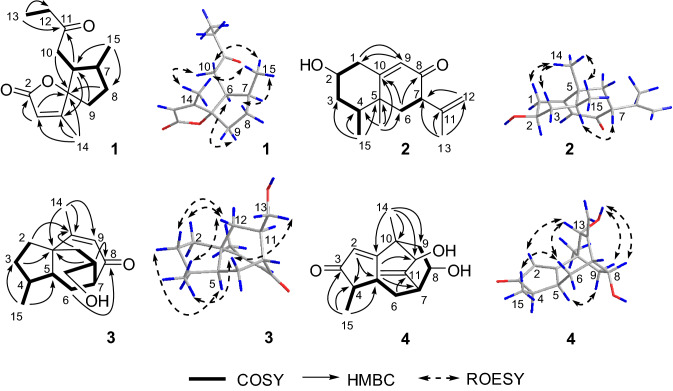
Key 2D NMR correlations of compounds **1**–**4**

The molecular formula of aquilarisinolide (**1**) was determined to be C_14_H_20_O_3_ based on ^13^C NMR data (Table 1) and the positive ion at *m/z* 259.1307 [M + Na]^+^ (calcd for C_14_H_20_NaO_3_, 259.1310) in the HRESIMS. The NMR data (Table 1) indicated the presence of one carbonyl group (*δ*_C_ 209.0), one α,β-unsaturated γ-lactone [*δ*_H_ 5.79 (br s); *δ*_C_ 172.0, 169.0, 118.8, and 99.4] [6], three methyl groups [*δ*_H_ 2.07 (s), 1.04 (t, *J* = 7.5 Hz), and 0.84 (d, *J* = 7.2 Hz); *δ*_C_ 15.5, 15.3, and 8.0], four methylenes, and two methines. Based on the COSY correlations (Fig. 2), two connections, C-9-C-8-C-7-(C-15)-C-6-C-10 and C-12-C-13 were deduced. Through its HMBC spectrum, correlations from H_3_-14 to C-3, C-4, and C-5, from H-3 to C-2 and C-5, from H_2_-8 and H_2_-10 to C-5, from H-6 to C-11, and from H_3_-13 to C-11 (Fig. 2) were observed. Combining these 2D NMR correlations and the HRESIMS, the planar structure of **1**, with a spiro ring system, was deduced to be 4,7-dimethyl-6-(2-oxobutyl)-1-oxaspiro[4.4]non-3-en-2-one. The relative configuration of **1** was deduced from its ROESY spectrum. H-6 was first assumed to be α-oriented; thus, the C-6-C-10 bond should be β-oriented. Based on the ROESY correlations of H_3_-14/H_2_-10 and H_3_-15/H_2_-10, the C-4-C-5 bond and 7-Me should be β-oriented, and thus, the C-5-O bond, H-6, and H-7 should be α-oriented.

**Table 1:** ^1^H and ^13^C NMR data of **1** and** 2** (*δ* in ppm, *J* in Hz)

No.	**1** in CDCl_3_		**2** in methanol-*d*_4_	
	*δ*_H_ (800 MHz)	*δ*_C_ (201 MHz)	*δ*_H_ (600 MHz)	*δ*_C_ (151 MHz)
1			2.50, ddd (11.5, 5.2, 1.5), α-H 2.44, ddd (11.5, 11.5, 1.3), β-H	43.1
2		172.0	3.57, m	72.3
3	5.79, br s	118.8	1.86, m, α-H 1.51, m, β-H	40.2
4		169.0	1.84, m	36.9
5		99.4		40.7
6	3.07, m	47.2	2.01, m	38.1
7	2.64, m	35.0	3.09, dd (9.9, 6.7)	51.6
8	2.16, m, α-H 1.53, m, β-H	32.0		202.0
9	2.07, m, β-H 1.94, m, α-H	34.5	5.79, d (1.2)	125.2
10	2.44, dd (17.1, 10.7) 2.25, dd (17.1, 4.4)	38.8		170.1
11		209.0		145.1
12	2.40, m	35.9	4.90, m 4.71, m	114.1
13	1.04, t (7.5)	8.0	1.75, br s	20.8
14	2.07, s	15.5	1.12, s	20.4
15	0.84, d (7.2)	15.3	0.98, d (6.7)	16.0

According to ^13^C NMR data (Table 1) and HRESIMS, the molecular formula of compound **2** was deduced to be C_15_H_22_O_2_. Its NMR data indicated the presence of one terminal double bond [*δ*_H_ 4.90 (1H, m) and 4.71 (1H, m); *δ*_C_145.1 and 114.1], one α,β-unsaturated ketone [*δ*_H_ 5.79 (1H, d, *J* = 1.2 Hz); *δ*_C_ 202.0, 170.1, and 125.2], three methyl groups [*δ*_H_ 1.75 (3H, br s), 1.12 (3H, s), and 0.98 (3H, d, *J* = 6.7 Hz)], three methylenes, three methines including one oxygenated group [*δ*_H_ 3.57 (1H, m); *δ*_C_ 72.3], and one quaternary carbon (*δ*_C_ 40.7). Two fragments of C-1-C-2-C-3-C-4-C-15 and C-6-C-7 were deduced by correlations (Fig. 2) from its COSY spectrum. Based on the key HMBC correlations from H-1 to C-9, from H-9 to C-1, from H_3_-14 and C-4, C-5, C-6, and C-10, from H_2_-6 to C-8 and C-10, and from H_3_-13 to C-7, C-11, and C-12, the planar structure of **2** was elucidated as 2-hydroxyeremophila-9,11-dien-8-one with an eremophilane skeleton. In order to deduce the relative configuration of **2**, H-2 was assumed to be α-oriented. Because a large coupling constant between H-2 and H-1β (*J*_1β,2_ = 11.5 Hz) was observed, the orientations of the two protons of C-1 were determined. In the ROESY spectrum of **2**, the correlations of H-1β/H_3_-14, H_3_-14/H_3_-15, and H-4/H-7 were observed. Thus, 4-Me and 5-Me should be β-oriented and H-7 should be α-oriented.

Compound **3** had the molecular formula C_15_H_22_O_2_ based on its ^13^C NMR data (Table 2) and the positive ion at *m*/*z* 257.1516 [M + Na]^+^ (calcd for C_15_H_22_NaO_2_, 257.1518) in the HRESIMS. The ^1^H NMR spectrum showed resonances for one trisubstituted double bond [*δ*_H_ 5.90 (br s)], as well as two methyl groups [*δ*_H_ 2.03 (d, *J* = 1.2 Hz) and 1.02 (d, *J* = 7.2 Hz)] (Table 2). The ^13^C NMR spectrum showed resonances for 15 carbon signals indicating the presence of one α,β-unsaturated ketone (*δ*_C_ 209.8, 172.5, and 130.0), two methyl groups (*δ*_C_ 22.5 and 17.3), five methylenes including one oxygenated group (*δ*_C_ 64.1), four methines, and one quaternary carbon atom (*δ*_C_ 53.5). By comparing its NMR data with those of daphnauranol A (**6**) and daphnauranol B (**7**) [7], compound **3** was deduced to be this type of sesquiterpenoid.

**Table 2:** ^1^H and ^13^C NMR data of **3** and** 4** (*δ* in ppm, *J* in Hz)

No.	**3** in methanol-*d*_4_		**4** in DMSO-*d*_6_		**4** in CDCl_3_	
	*δ*_H_ (800 MHz)	*δ*_C_ (201 MHz)	*δ*_H_ (600 MHz)	*δ*_C_ (151 MHz)	*δ*_H_ (500 MHz)	*δ*_C_ (126 MHz)
1		53.5		189.7		187.9
2	1.99, m, α-H 1.64, m, β-H	36.3	5.94, d (1.1)	126.7	5.94, d (0.7)	128.2
3	1.85, m, α-H 1.41, m, β-H	34.6		210.7		212.1
4	2.25, m	37.1	2.48, m	43.5	2.55, m	44.4
5	2.02, m	50.9	3.40, m	41.5	3.47, m	42.2
6	1.71, td (12.8, 2.5), β-H 1.50, m, α-H	20.1	2.36, m, β-H 0.86, m, α-H	35.4	2.48, m, β-H 1.10, m, α-H	35.2
7	2.72, m	50.2	2.62, m	43.5	2.82, m	43.8
8		209.8	4.13, m	64.5	4.40, m	66.9
9	5.90, br s	130.0	1.98, dd (14.0, 10.2), β-H 1.55, m, α-H	37.7	2.26, dd (14.3, 10.3), β-H 1.72, dd (14.3, 5.2), α-H	37.7
10		172.5		42.8		43.0
11	1.87, m	37.2		153.6		153.1
12	1.51, m, α-H 1.31, dd (13.5, 9.8), β-H	31.1	4.97, br s 4.95, br s	115.9	5.11, br s 5.12, br s	117.7
13	3.52, dd (10.8, 6.0) 3.47, dd (10.8, 8.3)	64.1	3.58, d (4.0)	74.0	3.82, br s	75.5
14	2.03, d (1.2)	22.5	1.17, s	24.3	1.31, s	24.3
15	1.02, d (7.2)	17.3	0.89, d (7.4)	9.9	1.05, d (7.5)	10.1
8-OH			4.84, d (3.0)			
13-OH			5.11, d (4.0)			

From the COSY correlations of **3** (Fig. 2), one moiety of C-2-C-3-C-4-(C-15)-C-5-C-6-C-7-C-11-(C-13)-C-12 was elucidated. According to the HMBC correlations (Fig. 2) from H-2 to C-10, from H-3 to C-1, from H_3_-15 to C-3 and C-5, from H_2_-6 to C-8, from H-11 to C-1 and C-8, from H_2_-12 to C-10, and from H_3_-14 to C-1, C-9, and C-10, the planar structure of **3** with a 5/6/7 ring system was elucidated as shown in Fig. 2. Its structure was very similar to that of daphnauranol A, except for the lack of a hydroxy group at C-7 in **3**. In the ROESY spectrum of **3**, correlations of H-2β/H-12β, H-3β/H-12β, H_3_-15/H-6β, H_3_-15/H-12β, H-6β/H_2_-13, and H-3α/H-5 were observed. The relative configuration of **3** was deduced to be 13-hydroxydaphnauran-9-en-8-one as shown in Fig. 2.

Compound **4** was assigned the molecular formula C_15_H_20_O_3_, as determined by ^13^C NMR data (Table 2) and the positive ion at *m/z* 271.1314 [M + Na]^+^ (calcd for C_15_H_20_NaO_3_, 271.1310) in the HRESIMS. The IR spectrum showed absorption bands for hydroxy groups (3424 cm^−1^), an α, β-unsaturated ketone (1687 cm^−1^), and an exocyclic double bond (3071 cm^−1^). The ^1^H and ^13^C NMR data in DMSO-*d*_6_ (Table 2) indicated the presence of one α,β-unsaturated ketone [*δ*_H_ 5.94 (d, *J* = 1.1 Hz); *δ*_C_ 210.7, 189.7, and 126.7], one exocyclic double bond [*δ*_H_ 4.97 (br s) and 4.95 (br s); *δ*_C_ 153.6 and 115.9], two methyl groups [*δ*_H_ 1.17 (s) and 0.89 (d, *J* = 7.4 Hz); *δ*_C_ 22.5 and 17.3], two methylenes, five methines including two oxygenated groups [*δ*_H_ 4.13 (m) and 3.58 (d, *J* = 4.0 Hz); *δ*_C_ 74.0 and 64.5), one quaternary carbon atom (*δ*_C_ 42.8), and two hydroxy groups [*δ*_H_ 5.11 (d, *J* = 4.0 Hz) and 4.84 (d, *J* = 3.0 Hz)]. According to the COSY correlations (Fig. 2), a fragment comprising of C-15-C-4-C-5-C-6-C-7-C-8-C-9 was deduced. By key HMBC correlations from H-2 to C-4 and C-5, from H_3_-15 to C-3 and C-5, from H_2_-6 to C-11, from H-12 to C-7 and C-13, and from H_3_-14 to C-1, C-9, C-10, and C-13, compound **4** was elucidated to be 8,13-dihydroxyrotunda-1,11-dien-3-one, with a very rare tricyclic rotundane skeleton. The relative configuration of **4** was deduced from its ROESY spectrum. The ROESY correlations (Fig. 2) of H_3_-15/H-6β and H-6β/H-13 indicated that 13-OH should be α-oriented; the ROESY correlations of 13-OH/H-8, 13-OH/H-9β, and H-5/H-9α indicated that 8-OH and H-5 should also be α-oriented.

**Fig. 3:**
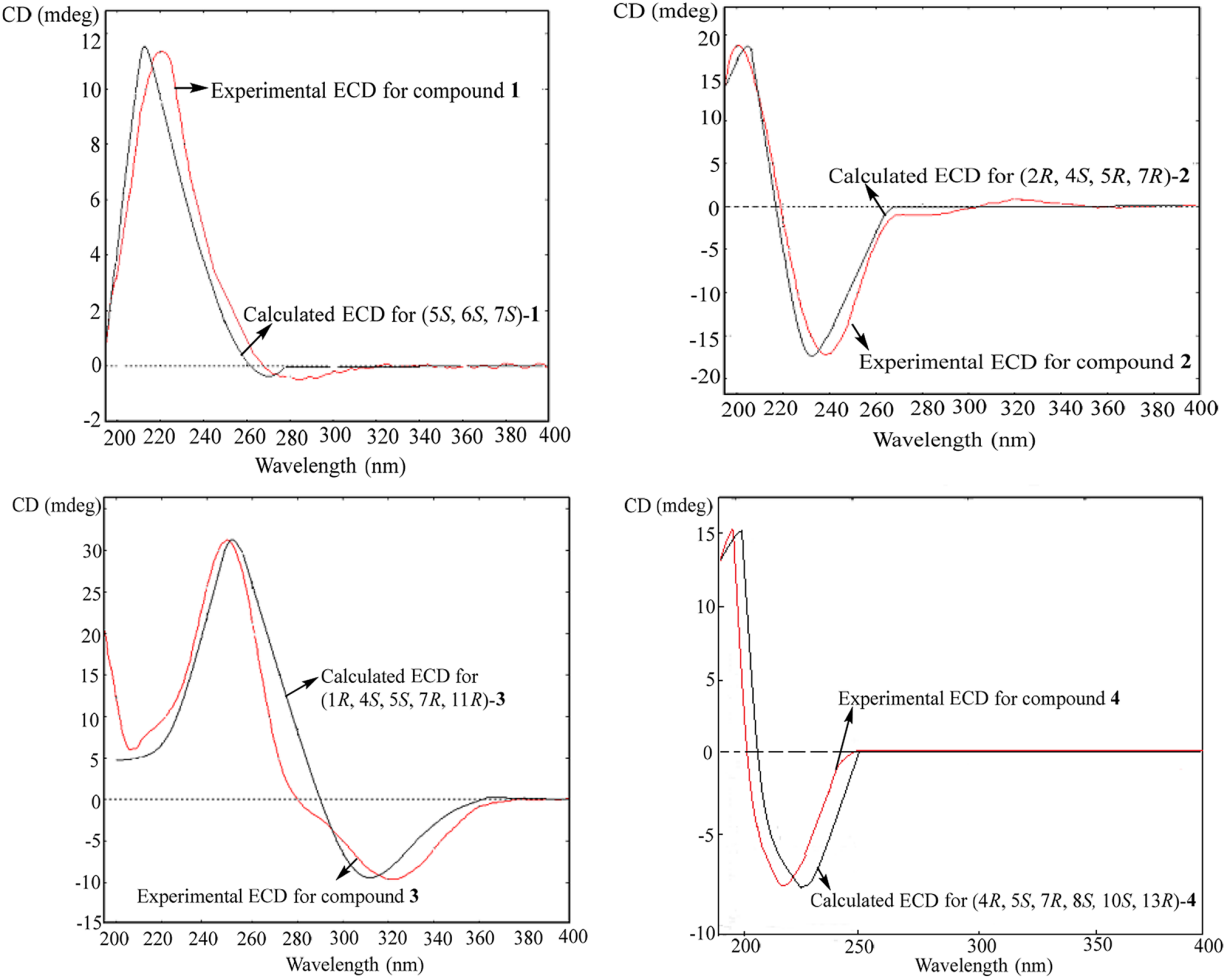
Experimental and computed ECD spectra of **1**–**4**

The absolute configurations of **1**–**4** were determined to be 5*S*,6*S*,7*S*-**1**, 2*R*,4*S*,5*R*,7*R*-**2**, 1*R*,4*S*,5*S*,7*R*,11*R*-**3**, and 4*R*,5*S*,7*R*,8*S*,10*S*,13*R*-**4** (Fig. 1), by comparison of the experimental electronic circular dichroism (ECD) spectra with the theoretical results (Fig. 3).

5,6-Dihydroxy-2-(2-phenylethyl)chromone (**5**) was recently reported with NMR data measured in CDCl_3_ [8]. Its NMR data in methanol-*d*_4_ are shown in Table 3. The structure of 6,7-dimethoxy-2-[2-(3-methoxyphenyl)ethyl]chromone (**12**) was found in the SciFinder database. However, no literature was provided in the database. The NMR data of **12** are presented in this paper (Table 3). The relative configuration of compound **15** has been reported [9]. Its absolute configuration was determined to be (5*S*,6*S*,7*S*,8*R*)-8-chloro-2-(2-phenylethyl)-5,6,7-trihydroxy-5,6,7,8-tetrahydrochromone by ECD calculations (Supplementary Material, Fig. S39). Daphnauranol A (**6**) [7], daphnauranol B (**7**) [7], 2-(2-phenylethyl)chromone (**8**) [10], 6-methoxy-2-(2-phenylethyl)chromone (**9**) [11], 6-methoxy-2-[2-(3-methyoxyphenyl)ethyl]chromone (**10**) [11], 6,7-dimethoxy-2-(2-phenylethyl)chromone (**11**) [11], 6,7-dimethoxy-2-[2-(4-methoxyphenyl)ethyl]chromone (**13**) [12], isoagarotetrol (**14**) [13], 1-hydroxy-1,5-diphenylpentan-3-one(**16**) [14], and 3-hydroxy-1,5-diphenylpentan-1-one (**17**) [14] were determined by comparing their obtained spectroscopic data with those reported in the literature.

**Table 3:** ^1^H and ^13^C NMR data of **5** and **12** in methanol-*d*_4_ (*δ* in ppm, *J* in Hz)

No.	**5**		**12**	
	*δ*_H_ (500 MHz)	*δ*_C_ (126 MHz)	*δ*_H_ (800 MHz)	*δ*_C_ (201 MHz)
2		172.1		170.7
3	6.05, s	109.5	6.11, s	110.1
4		185.1		179.9
5		146.3	7.45, s	104.9
6		138.8		149.4
7	7.13, d (8.5)	123.0		156.7
8	6.59, d (8.5)	111.1	7.14, s	101.1
9		153.4		154.5
10		111.7		117.4
1′		141.3		142.9
2′	7.23, m	129.5	6.78, br s	115.1
3′	7.25, m	129.6		161.3
4′	7.16, m	127.5	6.75, dd (8.1, 2.3)	112.9
5′	7.25, m	129.6	7.17, dd (8.1, 7.6)	130.6
6′	7.23, m	129.5	6.79, br d (7.6)	121.7
7′	3.09, br t (7.2)	33.8	3.06, t (7.5)	34.1
8′	3.00, br t (7.2)	37.0	3.00, t (7.5)	36.9
6-OMe			3.91, s	56.6
7-OMe			3.97, s	57.0
3′-OMe			3.72, s	55.5

### Neuroprotective Activities

CORT-induced PC12 cell damage is used as an in vitro experimental model for depression studies [15, 16], while MPP^+^ has been widely used as a neurotoxin to induce Parkinson’s disease (PD) symptoms in cells or rodents’ models [17, 18]. Additionally, BACE1 plays a critical role in Alzheimer’s disease (AD) pathophysiology and thus, BACE1 inhibition is considered to be a therapeutic approach for AD [19].

Compounds **1**–**12** and **14**–**17** were evaluated for their protective activities against PC12 cell injury induced by CORT and MPP^+^ and inhibitory activities against BACE1. Compound **13** was not evaluated for all activities due to an insufficient amount. The results are presented in Table 4. Compared with the negative control, compounds **4**, **5**, **7**, **10**, **14**, and **16** showed significant protective effects on CORT-induced injury in PC12 cells at a concentration of 20 μM (*P* < 0.001), while compounds **3** and **11** showed weak protective activities (*P* < 0.05). Other tested compounds were inactive (*P* > 0.05). Compared with the negative control, compound **14** showed a significant protective effect on MPP^+^-induced injury in PC12 cells at a concentration of 20 μM (*P* < 0.001); compound **4** showed a moderate effect on MPP^+^-induced injury in PC12 cells at a concentration of 20 μM (*P* < 0.01); and compound **16** showed a weak protective activity (*P* < 0.05). Other tested compounds were inactive (*P* > 0.05). The BACE1-inhibitory activities of all tested compounds were very weak with inhibition less than 30% at a concentration of 20 μM, compared with the positive control LY2886721 with 75.39% inhibition at a concentration of 0.2 μM.

**Table 4:** The effects of compounds from *A. sinensis* on PC12 cell injury induced by corticosterone and MPP^+^ and on BACE1 inhibition^a^

Compound	PC12 cell injury induced by corticosterone	PC12 cell injury induced by MPP^+^	BACE1 inhibition
	Survival rate ± SD (%)^b^	Survival rate ± SD (%)^b,c^	Inhibition rate ± SD (%)
**1**	59.71 ± 1.52	70.63 ± 1.85	20.33 ± 0.32
**2**	59.15 ± 1.26	69.77 ± 0.60	6.64 ± 0.40
**3**	62.40 ± 0.90*	69.42 ± 2.73	21.62 ± 1.03
**4**	80.20 ± 1.97***	73.52 ± 1.25**	20.45 ± 0.48
**5**	78.71 ± 2.03***	72.06 ± 3.22	16.25 ± 0.37
**6**	59.74 ± 1.02	69.53 ± 1.17	9.43 ± 0.12
**7**	72.56 ± 2.05***	72.66 ± 1.11	6.84 ± 0.56
**8**	59.62 ± 2.12	71.09 ± 0.65	18.65 ± 0.85
**9**	60.35 ± 2.64	72.68 ± 3.00	0.19 ± 0.29
**10**	73.73 ± 1.11***	72.51 ± 2.12	26.55 ± 1.11
**11**	64.94 ± 2.27*	68.62 ± 2.96	26.86 ± 0.17
**12**	63.80 ± 2.55	67.58 ± 1.53	7.19 ± 0.56
**14**	79.45 ± 0.95***	74.89 ± 0.57***	16.55 ± 1.38
**15**	59.69 ± 1.07	71.88 ± 2.79	18.84 ± 0.66
**16**	71.76 ± 2.27***	74.83 ± 2.15*	9.78 ± 0.25
**17**	60.70 ± 2.51	71.22 ± 1.42	18.67 ± 0.45
desipramine (positive control)	89.66 ± 0.78***	–	–
negative control	59.92 ± 0.33	69.72 ± 1.77	–
blank	100.00 ± 0.22	100 ± 1.04	–
vitamin E (positive control)	–	75.70 ± 0.64***	
LY2886721 (positive control)	–	–	75.39 ± 0.82

^a^The concentration of the tested compounds was 20 μM; the concentrations of desipramine, vitamin E, and LY2886721 were 10 μM, 0.2 μM, and 0.2 μM, respectively

^b^Compared with the negative control, *P < 0.05, **P < 0.01, ***P < 0.001

^c^For compounds **5**, **7**–**10**, and **16**, the negative control was 69.05 ± 0.49% and the blank was 100 ± 0.98%; For compound **12**, the negative control was 67.66 ± 1.26% and the blank was 100 ± 0.98%

## Experimental Section

### General Experimental Procedures

Optical rotations were recorded using a JASCO P-1020 polarimeter (Jasco Corp., Tokyo, Japan). UV spectra were recorded on a Shimadzu UV-2401 PC spectrophotometer (Shimadzu, Kyoto, Japan). Electronic circular dichroism (ECD) spectra were recorded on a Chirascan CD spectrometer (Applied Photophysics Ltd., Leatherhead, UK). IR spectra were measured on a Bruker Tensor 27 FTIR Spectrometer (Bruker Corp., Ettlingen, Germany) with KBr disks. ^1^H and ^13^C NMR spectra were collected on Bruker DRX-500, Avance III-600, and Ascend™ 800 MHz NMR spectrometers (Bruker Corporation, Karlsruhe, Germany), with TMS as an internal standard. ESIMS and HRESIMS analyses were performed on an API QSTAR Pulsar 1 spectrometer (Applied Biosystems/MDS Sciex, Foster City, CA, USA). Silica gel G (80–100 and 300–400 mesh, Qingdao Meigao Chemical Co., Ltd., Qingdao, China), C_18_ silica gel (40–75 μm, Fuji Silysia Chemical Ltd., Aichi, Japan), and Sephadex LH-20 (GE Healthcare Bio-Sciences AB, Uppsala, Sweden) were used for column chromatography. Thin-layer chromatography (TLC) spots were visualized under UV light at 254 nm and by dipping in 5% H_2_SO_4_ in alcohol followed by heating. Semipreparative high-performance liquid chromatography (HPLC) was performed on an Agilent 1200 series pump (Agilent Technologies, Santa Clara, USA) equipped with a diode array detector, a Welch Ultimate AQ-C_18_ column (5 μm, *ϕ* 7.8 × 250 mm, Welch Materials Inc., Shanghai, China), an Agilent Zorbax SB-C_18_ column (5.0 μm, *ϕ* 9.4 × 250 mm), and a chiral-phase CD-Ph column (5.0 μm, *ϕ* 4.6 × 250 mm; Shiseido, Japan). The absorbance from the MTS assay was measured by a Thermo Multiskan FC microplate reader (Waltham, MA, USA). The fluorescence values in the BACE1-inhibitory activity assay were read by a FlexStation 3 Multi-Mode microplate reader (Molecular Devices, San Jose, CA, USA). Desipramine was purchased from Beijing Pujing Kangli Technology Co., Ltd. (Beijing, China); corticosterone, penicillin, streptomycin, MTS, MPP^+^, and the BACE1 kit were purchased from Sigma; MTS was purchased from Promega; DMEM, FBS, and PBS were obtained from Biofluids Inc. (Rockville, MD, USA); and LY2886721 (CAS No. 1262036–50-9) was from Shanghai Lanmu Chemical Co., Ltd. (Shanghai, China).

### Plant Material

The resinous heartwood of *A. sinensis* with the origin in Hainan, China was purchased from Xiamen Yanlaifu Pharmaceutical Co., Ltd., China (production lot number 140303), in December, 2017. The plant material was also identified by Prof. Shu-De Yang, at Yunnan University of Traditional Chinese Medicine, China. A voucher specimen (no. HN140303) was deposited at the Yunnan Key Laboratory for Wild Plant Resources, Kunming Institute of Botany, Chinese Academy of Sciences.

### Extraction and Isolation

The resinous heartwood of *A. sinensis* (0.5 kg) was ground into a powder and ultrasonically extracted with 95% EtOH at 60 ºC for half an hour. The extract was subjected to reduced pressure evaporation to yield a gum (53.8 g). The gum was dissolved in water and extracted successively with petroleum ether, EtOAc, and *n*-BuOH to yield fractions A (0.6 g), B (36.4 g), and C (14.7 g), respectively.

Fraction B was subjected to silica gel column chromatography (CC, petroleum ether-EtOAc, 50:1 → 0:1, v/v) to yield four main fractions (B1–B4). Fraction B1 was subjected to reversed-phase C_18_ (RP-C_18_) silica gel CC with MeOH-H_2_O (10% → 100%) to afford four main subfractions B1-1 to B1-4. Subfraction B1-1 (121.8 mg) was subjected to SephadexLH-20 CC (MeOH) to yield subfractions B1-1–1 and B1-1–2. Subfraction B1-1–1 (87.1 mg) was purified by silica gel CC (petroleum ether-EtOAc, 15:1) to yield **7** (17.6 mg). Subfraction B1-1–2 (22.9 mg) was purified by semipreparative HPLC (Agilent Zorbax SB-C_18_, CH_3_CN-H_2_O, 30:70, 2 mL/min) to yield **1** (1 mg, *t*_R_ = 40.197 min). Subfraction B1-2 (113.7 mg) was separated by silica gel CC (petroleum ether-EtOAc, 50:1) and semipreparative HPLC (Agilent Zorbax SB-C_18_, MeOH-H_2_O, 60:40, 2 mL/min) to yield **16** (4.2 mg, *t*_R_ = 33.431 min) and **17** (2.6 mg, *t*_R_ = 39.728 min). Subfraction B1-3 (658.2 mg) was subjected to Sephadex LH-20 CC (MeOH) to yield **8** (192.6 mg). Subfraction B1-4 (1.1 g) was subjected to Sephadex LH-20 CC (MeOH) to yield **5** (6.0 mg) and a mixture (953.4 mg). The mixture was purified by silica gel CC (petroleum ether-EtOAc, 50:1 → 10:1) to yield **9** (25.2 mg) and **10** (44.0 mg).

Fraction B2 was subjected to RP-C_18_ silica gel CC with MeOH-H_2_O (10% → 100%) to afford **11** (15.6 mg) recrystallized from MeOH and two other subfractions, B2-1 and B2-2. Subfraction B2-1 (436.3 mg) was purified by Sephadex LH-20 CC (MeOH) and semipreparative HPLC (Agilent Zorbax SB-C_18_, CH_3_CN-H_2_O, 30:70, 2 mL/min) to yield **3** (1.5 mg, *t*_R_ = 47.901 min), **2** (2.4 mg, *t*_R_ = 40.414 min), and **6** (1.8 mg, t_R_ = 27.272 min). Subfraction B2-2 (3.7 g) was separated by silica gel CC (CH_2_Cl_2_-acetone, 5:1) and semipreparative HPLC (Chiral CD-Ph, MeOH-H_2_O, 80:20, 1 mL/min) to obtain **12** (0.6 mg, *t*_R_ = 43.207 min) and **13** (1.2 mg, *t*_R_ = 35.603 min).

Fraction B3 was subjected to RP-C18 silica gel CC with MeOH-H_2_O (10% → 100%) to afford a main fraction (199.6 mg), which was purified by silica CC (petroleum ether-EtOAc, 2:1) and semipreparative HPLC (Welch Ultimate AQ-C_18_, MeOH-H_2_O, 17:83, 2 mL/min) to yield **4** (8.3 mg, *t*_R_ = 50.308 min).

Fraction B4 was subjected to RP-C_18_ silica gel CC with MeOH-H_2_O (10% → 100%) to afford two main subfractions B4-1 and B4-2. Subfraction B4-1 (872.5 mg) was subjected to Sephadex LH-20 CC (MeOH) and was further purified by silica gel CC (CH_2_Cl_2_-acetone, 8:1) to yield **14** (8.3 mg). Subfraction B4-2 (696.4 mg) was subjected to Sephadex LH-20 CC (MeOH) and was further purified by silica gel CC (CH_2_Cl_2_-acetone, 8:1) and semipreparative HPLC (Welch Ultimate AQ-C_18_, MeOH-H_2_O, 45:55, 2 mL/min) to yield **15** (24.4 mg, *t*_R_ = 27.000 min).

### Spectroscopic Data of Compounds

#### Aquilarisinolide (1)

White solid; [*α*]_D_^27^ + 71 (*c* 0.08, MeOH); UV (MeOH) *λ*_max_ (log*ε*) 215 (4.10) nm; ECD (*c* 0.016, MeOH): ∆*ε*_219 nm_ + 5.06; ^1^H and ^13^C NMR data, see Table 1; ESIMS *m/z* 259 [M + Na]^+^; HRESIMS *m/z* 259.1307 [M + Na]^+^ (calcd for C_14_H_20_NaO_3_, 259.1310).

#### (2*R*,4*S*,5*R*,7*R*)-2-Hydroxyeremophila-9, 11-dien-8-one (2)

White solid; [*α*]_D_^23^ − 79 (*c* 0.12, MeOH); UV (MeOH) *λ*_max_ (log*ε*) 240 (3.08) nm; ECD (*c* 0.018, MeOH): ∆*ε*_239 nm_ − 6.83, ∆*ε*_201 nm_ + 7.36; ^1^H and ^13^C NMR data, see Table 1; ESIMS *m/z* 257 [M + Na]^+^; HRESIMS *m/z* 257.1512 [M + Na]^+^ (calcd for C_15_H_22_NaO_2_, 257.1518).

#### (1*R*,4*S*,5*S*,7*R*,11*R*)-13-Hydroxydaphnauran-9-en-8-one (3)

White solid; [*α*]_D_^26^ − 37 (*c* 0.07, MeOH); UV(MeOH) *λ*_max_ (log*ε*) 390 (1.85), 246 (3.97), 201 (3.70) nm; ECD (*c* 0.018, MeOH): ∆*ε*_322 nm_ − 3.78, ∆*ε*_249 nm_ + 12.15; ^1^H and ^13^C NMR data, see Table 2; ESIMS *m/z* 257 [M + Na]^+^; HRESIMS *m/z* 257.1516 [M + Na]^+^ (calcd for C_15_H_22_NaO_2_, 257.1518).

#### (4*R*,5*S*,7*R*,8*S*,10*S*,13*R*)-8,13-Dihydroxyrotunda-1,11-dien-3-one (4)

Yellow oil; [*α*]_D_^27^ − 78 (*c* 0.09, MeOH); UV (MeOH) *λ*_max_ (log*ε*) 237 (3.87), 196 (3.73) nm; ECD (*c* 0.009, MeOH): ∆*ε*_226 nm_ − 6.46, ∆*ε*_197 nm_ + 12.42; IR *ν*_max_ (KBr) 3424, 3071, 2970, 2936, 2877, 1687, 1597, 1457, 1187, 1043, 1027, 975 cm^−1^; ^1^H and ^13^C NMR data, see Table 2; ESIMS *m/z* 271 [M + Na]^+^; HRESIMS *m/z* 271.1314 [M + Na]^+^ (calcd for C_15_H_20_NaO_3_, 271.1310).

#### (5*S*,6*S*,7*S*,8*R*)-8-Chloro-2-(2-phenylethyl)-5,6,7-trihydroxy-5,6,7,8-tetrahydrochromone (15)

Yellow solid; [*α*]_D_^26^ + 12 (*c* 0.12, MeOH); ECD (*c* 0.009, MeOH): ∆*ε*_305 nm_ + 0.32, ∆*ε*_232 nm_ + 1.33; ESIMS *m/z* 359 [M + Na]^+^, 695 [2 M + Na]^+^.

### Computational Methods

All DFT and TD-DFT calculations were carried out at 298 K in the gas phase with Gaussian 09 [21]. Conformational searches were carried out at the molecular mechanics level of theory employing MMFF force fields. The conformers with relative energy within 10 kcal/mol of the lowest-energy conformer were selected and further geometry optimized at the B3LYP/6–311 +  + G (2d, p) level. All the lowest-energy conformers, which correspond to 99% of the total Boltzmann distribution, were selected for ECD spectra calculation. The Boltzmann factor for each conformer was calculated based on Gibbs free energy. Vibrational analysis at the B3LYP/6–311 +  + G (2d, p) level of theory resulted in no imaginary frequencies, confirming the considered conformers as real minima. TDDFT was employed to calculate excitation energy (in nm) and rotatory strength R in dipole velocity form, at the B3LYP/6–311 +  + G (2d, p) level [22].

### Biological Assays

#### Corticosterone-Induced Damage in PC12 Cellular Assay

Poorly differentiated PC12 cells (Cell Bank of Kunming Institute of Zoology, Chinese Academy of Sciences, Kunming, China) were maintained in DMEM supplemented with 10% fetal bovine serum (FBS), penicillin (100 U/mL), and streptomycin (100 μg/mL) and incubated with 5% CO_2_ at 37 ºC. The poorly differentiated PC12 cells were divided into the following groups: blank (untreated), negative control (150 μM CORT), positive control (150 μM CORT plus 10 μM desipramine), and compounds (20 μM of each tested compound plus 150 μM CORT). Briefly, the poorly differentiated PC12 cells were seeded into 96-well culture plates at a density of 1 × 10^4^ cells/well. After culturing for 24 h, compounds were added to the wells. After 48 h, MTS solution was added to each well. The absorbance was measured at 492 nm using a Thermo Multiskan FC [15].

#### MPP^+^-Induced Damage in PC12 Cellular Assay

Poorly differentiated PC12 cells were maintained in DMEM medium supplemented with 10% fetal bovine serum (FBS), penicillin (100 U/mL), streptomycin (100 μg/mL), and incubated at 5% CO_2_ and 37 ºC. The cells were divided into the following groups: blank (untreated), negative control (750 µM MPP^+^), positive control (0.2 µM vitamin E plus 750 μM MPP^+^), and compounds (20 μM of each tested compound plus 750 μM MPP^+^). Briefly, poorly differentiated PC12 cells were seeded into 96-well culture plates at a density of 1 × 10^4^ cells/well. After 23 h of culture, each compound or vitamin E was added to the wells. One hour later, MPP^+^ was added. At 24 h after MPP^+^ exposure, MTS was added; and two hours later, absorbance at 492 nm was read using a Thermo Multiskan FC.

#### BACE1-Inhibitory Activity Assay

The BACE-inhibitory assay was carried out according to the manufacturer described protocol available from Sigma-Aldrich [20]. Detection was based on fluorescence resonance energy transfer (FRET) technology, by which the enhanced fluorescence signal can be observed after the substrate is cleaved by BACE1. In the positive control group, 76 μL of buffer, 20 μL of 50 μM BACE1 substrate solution, 2 μL of 0.3 units/μL BACE1 enzyme solution, and 2 μL of LY2886721 were added. The final concentration of LY2886721 was 0.2 μM. In the compound groups, 76 μL of buffer, 20 μL of 50 μM BACE1 substrate solution, 2 μL of 0.3 units/μL BACE1 enzyme solution, and 2 μL of tested sample solution were added. The final concentration of compounds was 20 μM. After adding the enzyme, the zero point of the fluorescence value (excitation 320 nm, absorption 405 nm) was measured immediately with FlexStation 3. Then, the plate was incubated for 2 h at 37 ºC, and the fluorescence value was measured again by FlexStation 3.

## Conclusion

Four new and 13 known compounds were isolated from the resinous heartwood of *A. sinensis*. Six compounds showed significant protective effects on CORT-induced injury in PC12 cells, while one compound exhibited a significant protective effect on MPP^+^-induced injury in PC12 cells. These active compounds are worth further evaluation for neuroprotective activities.

## Supplementary Information

Below is the link to the electronic supplementary material.

Supplementary file1 (PDF 2390 kb)
