# Supplementary material for: Sesquiterpenoids and 2-(2-Phenylethyl)chromone Derivatives from the Resinous Heartwood of Aquilaria sinensis
Source: Nat Prod Bioprospect. 2021 Jun 1;11(5):545–55. doi: 10.1007/s13659-021-00313-0 (PMC8390629; doi:10.1007/s13659-021-00313-0)
Supplement: Supplementary file 1 — Supplementary file1 (PDF 2390 kb) [file 13659_2021_313_MOESM1_ESM.pdf]

Supplementary Material for

Sesquiterpenoids and 2-(2-Phenylethyl)chromone Derivatives from the Resinous Heartwood of *Aquilaria sinensis*

Shu-Ya Wei[1,2] · Dong-Bao Hu[3] · Meng-Yuan Xia[2] · Ji-Feng Luo[2] · Hui Yan[2] · Jing-Hua Yang[1] · Yun-Song Wang[1] · Yue-Hu Wang[2]

Shu-Ya Wei and Dong-Bao Hu contributed equally to this work.

✉ Yun-Song Wang
wangys@ynu.edu.cn

✉ Yue-Hu Wang
wangyuehu@mail.kib.ac.cn

[1] Key Laboratory of Medicinal Chemistry for Natural Resource, Ministry of Education, School of Chemical Science and Technology, School of Pharmacy, Yunnan University, Kunming 650091, People's Republic of China

[2] Key Laboratory of Economic Plants and Biotechnology, Yunnan Key Laboratory for Wild Plant Resources, and State Key Laboratory of Phytochemistry and Plant Resources in West China, Chinese Academy of Sciences, Kunming 650201, People's Republic of China

[3] School of Chemical Biology and Environment, Yuxi Normal University, Yuxi 653100, People's Republic of China

Contents

Computational methods for ECD of compounds **1**–**4** and **15**.
**Figure S1.** ^{1}H NMR spectrum of **1** ($CDCl_3$, 800 MHz).
**Figure S2.** ^{13}C NMR spectrum of **1** ($CDCl_3$, 201 MHz).
**Figure S3.** HSQC spectrum of **1**.
**Figure S4.** ^{1}H–^{1}H COSY spectrum of **1**.
**Figure S5.** HMBC spectrum of **1**.
**Figure S6.** ROESY spectrum of **1**.
**Figure S7.** HRESIMS spectrum of **1**.
**Figure S8.** ECD spectrum of **1**.
**Figure S9.** ^{1}H NMR spectrum of **2** (methanol-d_4, 600 MHz).
**Figure S10.** ^{13}C NMR spectrum of **2** (methanol-d_4, 151 MHz).
**Figure S11.** HSQC spectrum of **2**.
**Figure S12.** ^{1}H–^{1}H COSY spectrum of **2**.
**Figure S13.** HMBC spectrum of **2**.
**Figure S14.** ROESY spectrum of **2**.
**Figure S15.** HRESIMS spectrum of **2**.
**Figure S16.** ECD spectrum of **2**.
**Figure S17.** ^{1}H NMR spectrum of **3** (methanol-d_4, 800 MHz).
**Figure S18.** ^{13}C NMR spectrum of **3** (methanol-d_4, 201 MHz).
**Figure S19.** HSQC spectrum of **3**.
**Figure S20.** ^{1}H–^{1}H COSY spectrum of **3**.
**Figure S21.** HMBC spectrum of **3**.
**Figure S22.** ROESY spectrum of **3**.
**Figure S23.** HRESIMS spectrum of **3**.
**Figure S24.** ECD spectrum of **3**.
**Figure S25.** ^{1}H NMR spectrum of **4** (DMSO-d_6, 600 MHz).
**Figure S26.** ^{13}C NMR spectrum of **4** (DMSO-d_6, 151 MHz).
**Figure S27.** HSQC spectrum of **4** in DMSO-d_6.
**Figure S28.** ^{1}H–^{1}H COSY spectrum of **4** in DMSO-d_6.
**Figure S29.** HMBC spectrum of **4** in DMSO-d_6.
**Figure S30.** ROESY spectrum of **4** in DMSO-d_6.
**Figure S31.** HRESIMS spectrum of **4**.
**Figure S32.** ECD spectrum of **4**.
**Figure S33.** ^{1}H NMR spectrum of **4** ($CDCl_3$, 500 MHz).
**Figure S34.** ^{13}C NMR spectrum of **4** ($CDCl_3$, 126 MHz).
**Figure S35.** HSQC spectrum of **4** in $CDCl_3$.
**Figure S36.** ^{1}H–^{1}H COSY spectrum of **4** in $CDCl_3$.
**Figure S37.** HMBC spectrum of **4** in $CDCl_3$.
**Figure S38.** ROESY spectrum of **4** in $CDCl_3$.
**Figure S39.** Experimental and computed ECD spectra of **15**.

Computational methods

All DFT and TD-DFT calculations were carried out at 298 K in the gas phase with Gaussian 09 [1]. Conformational searches were carried out at the molecular mechanics level of theory employing MMFF force fields [2-7]. The conformers with relative energy within 10 kcal/mol of the lowest-energy conformer were selected and further geometry optimized at the B3LYP/6-311++G (2d, p) level. All the lowest-energy conformers, which correspond to 99% of the total Boltzmann distribution, were selected for ECD spectra calculation. The Boltzmann factor for each conformer was calculated based on Gibbs free energy. Vibrational analysis at the B3LYP/6-311++G (2d, p) level of theory resulted in no imaginary frequencies, confirming the considered conformers as real minima. TDDFT was employed to calculate excitation energy (in nm) and rotatory strength R in dipole velocity form, at the B3LYP/6-311++G (2d, p) level.

References

1. M.J. Frisch, Gaussian 09. Rev.Cl (M.J. Frisch, et al. Gaussian, Inc., Pittsburgh PA, 2009)
2. J.-B. He, Y.-N. Ji, D.-B. Hu, S. Zhang, H. Yan, X.-C. Liu, H.-R. Luo, H.-J. Zhu, Tetrahedron Lett. **55**, 2684–2686 (2014)
3. C.-N. Wen, D.-B. Hu, X. Bai, F. Wang, Z.-H. Li, T. Feng, J.-K. Liu, Fitoterapia **109**, 179–184 (2016)
4. S. Zhang, D.-B. Hu, J.-B. He, K.-Y. Guan, H.-J. Zhu, Tetrahedron **70**, 869–873 (2014)
5. D.-B. Hu, S. Zhang, J.-B. He, Z.-J. Dong, Z.-H. Li, T. Feng, J.-K. Liu, Fitoterapia **104**, 50–54 (2015)
6. D.-B. Hu, W.-X. Li, Z.-Z. Zhao, T. Feng, R.-H. Yin, Z.-H. Li, J.-K. Liu, H.-J. Zhu, Tetrahedron Lett. **55**, 6530–6533 (2014)

Compound **1**:

Standard orientation:

Center Number	Atomic Number	Atomic Type	Coordinates (Angstroms) X	Y	Z
1	8	0	0.309554	1.223716	-1.146953
2	6	0	0.893036	2.190766	-0.356114
3	6	0	0.127372	2.239594	0.901699
4	6	0	-0.864652	1.338694	0.867377
5	6	0	-0.813399	0.616663	-0.473258
6	1	0	0.371110	2.928590	1.700392
7	6	0	-2.091542	0.790490	-1.327396
8	1	0	-2.556232	1.772961	-1.194098
9	1	0	-1.787482	0.711397	-2.377228
10	6	0	-2.996101	-0.383730	-0.932033
11	1	0	-3.721827	-0.627644	-1.715216
12	1	0	-3.578828	-0.134383	-0.036936
13	6	0	-2.045940	-1.575570	-0.621936
14	1	0	-2.009748	-2.231469	-1.500628
15	6	0	-0.613421	-0.934583	-0.478794
16	1	0	-0.101307	-1.107172	-1.432141
17	6	0	-2.557526	-2.432214	0.543631
18	1	0	-2.624828	-1.866120	1.479774
19	1	0	-1.924017	-3.306890	0.725842
20	1	0	-3.564733	-2.800873	0.315543
21	6	0	0.293398	-1.539140	0.621102
22	1	0	-0.116384	-1.335880	1.616752
23	1	0	0.295740	-2.628841	0.491634
24	6	0	1.754959	-1.120594	0.604085
25	6	0	2.656810	-1.522674	-0.550586
26	1	0	2.545446	-0.792453	-1.371258
27	1	0	2.287541	-2.487216	-0.921352
28	6	0	4.140985	-1.625230	-0.169699
29	1	0	4.290141	-2.349702	0.638026
30	1	0	4.527234	-0.657986	0.165217
31	1	0	4.731566	-1.945825	-1.035363
32	8	0	1.833751	2.846594	-0.727828
33	8	0	2.051150	0.016906	1.186172
34	6	0	-1.898787	1.119104	1.927747
35	1	0	-1.687196	1.739391	2.803368
36	1	0	-1.940706	0.074312	2.254727
37	1	0	-2.902273	1.378613	1.566858

Excitation energies and oscillator strengths:

Excited State 1: Singlet-A 3.1104 eV 398.62 nm f=0.0021 <S**2>=0.000
62 -> 65 -0.17416
64 -> 65 0.67383
64 -> 66 -0.10733

Excited State 2: Singlet-A 4.4794 eV 276.79 nm f=0.0003 <S**2>=0.000
63 -> 65 0.62043
63 -> 66 0.31744

Excited State 3: Singlet-A 4.7918 eV 258.74 nm f=0.0057 <S**2>=0.000
62 -> 65 0.67302
64 -> 65 0.17714

Excited State 4: Singlet-A 4.8321 eV 256.59 nm f=0.0100 <S**2>=0.000
62 -> 66 -0.14948
63 -> 65 0.12951
64 -> 66 0.66557

Excited State 5: Singlet-A 4.9320 eV 251.39 nm f=0.0004 <S**2>=0.000
63 -> 65 -0.30944
63 -> 66 0.61426
64 -> 66 0.12287

Excited State 6: Singlet-A 5.3193 eV 233.08 nm f=0.0072 <S**2>=0.000
61 -> 65 0.70458

Excited State 7: Singlet-A 5.9852 eV 207.15 nm f=0.1300 <S**2>=0.000
60 -> 65 0.14247
61 -> 66 -0.38268
62 -> 66 0.54872
64 -> 66 0.10438

Compound **2**:

Standard orientation:

Center Number	Atomic Number	Atomic Type	Coordinates (Angstroms) X	Y	Z
1	1	0	1.982202	-2.525753	0.536486
2	6	0	1.900460	-1.436779	0.625696
3	6	0	2.943986	0.748536	-0.027734
4	6	0	0.364679	0.550148	-0.029468
5	6	0	1.633895	1.213657	-0.690284
6	6	0	0.595879	-0.949742	0.061822
7	6	0	3.103034	-0.772686	-0.050956
8	1	0	2.997742	1.085593	1.016304
9	1	0	1.654329	0.839476	-1.725555
10	1	0	3.146731	-1.112161	-1.099972
11	1	0	1.983819	-1.208813	1.703438
12	1	0	3.792719	1.217135	-0.548544
13	6	0	-0.850472	0.827034	-0.953231
14	1	0	-1.145768	1.879423	-0.879910
15	1	0	-0.536787	0.664369	-1.991364
16	6	0	-2.117750	-0.038888	-0.734589
17	1	0	-2.740102	0.091401	-1.631995
18	6	0	-0.385724	-1.866702	-0.312343
19	1	0	-0.159427	-2.929204	-0.274644
20	6	0	-1.666710	-1.517582	-0.721954
21	8	0	-2.604951	-2.319804	-1.107900
22	6	0	-3.011802	0.330607	0.451733
23	6	0	-3.536568	1.749347	0.434004
24	1	0	-4.299381	1.891246	1.205007
25	1	0	-3.984487	1.996678	-0.538085
26	1	0	-2.741360	2.483537	0.613434
27	6	0	-3.369277	-0.531520	1.407236
28	1	0	-4.042268	-0.230423	2.205792
29	1	0	-3.019191	-1.557905	1.426861
30	6	0	0.112376	1.130379	1.389086
31	1	0	0.977609	0.981867	2.042639
32	1	0	-0.743525	0.639191	1.857407
33	1	0	-0.094354	2.206576	1.350210
34	6	0	1.590822	2.749127	-0.761932
35	1	0	1.643419	3.206113	0.232648
36	1	0	0.685874	3.119338	-1.255167
37	1	0	2.448078	3.120423	-1.335993
38	8	0	4.269444	-1.201927	0.651335

39 1 0 5.037211 -0.793867 0.221009

Excitation energies and oscillator strengths:

Excited State 1: Singlet-A 2.7678 eV 447.96 nm f=0.0001 <S**2>=0.000
64 -> 65 -0.70161

Excited State 2: Singlet-A 3.9046 eV 317.53 nm f=0.0119 <S**2>=0.000
62 -> 65 0.21545
63 -> 65 0.67221

Excited State 3: Singlet-A 4.9490 eV 250.52 nm f=0.0337 <S**2>=0.000
61 -> 65 0.66727
62 -> 65 0.21156

Excited State 4: Singlet-A 5.1849 eV 239.13 nm f=0.3706 <S**2>=0.000
60 -> 65 0.17801
61 -> 65 -0.22606
62 -> 65 0.60205
63 -> 65 -0.18995

Excited State 5: Singlet-A 5.6854 eV 218.07 nm f=0.0204 <S**2>=0.000
56 -> 65 -0.17914
57 -> 65 -0.13813
58 -> 65 -0.28387
59 -> 65 0.11062
60 -> 65 -0.56934
62 -> 65 0.11512

Excited State 6: Singlet-A 5.7628 eV 215.14 nm f=0.0016 <S**2>=0.000
64 -> 66 -0.69966

Excited State 7: Singlet-A 5.7898 eV 214.14 nm f=0.0055 <S**2>=0.000
58 -> 65 0.25702
59 -> 65 0.64341

Excited State 8: Singlet-A 6.0494 eV 204.95 nm f=0.0265 <S**2>=0.000
55 -> 65 0.11776
58 -> 65 0.55482
59 -> 65 -0.23281
60 -> 65 -0.29441
62 -> 65 0.13880

Excited State 9: Singlet-A 6.3385 eV 195.61 nm f=0.0077 <S**2>=0.000
56 -> 65 -0.19086
57 -> 65 -0.58188

58 -> 65	0.15410
60 -> 65	0.14168
64 -> 68	-0.18899
64 -> 69	0.12622

Compound **3**:

Standard orientation:

Center Number	Atomic Number	Atomic Type	Coordinates (Angstroms) X	Y	Z
1	6	0	-0.670648	0.579583	-0.592764
2	6	0	-1.110745	0.188152	0.876099
3	6	0	-2.566911	-0.400187	0.776260
4	6	0	-2.865400	-0.488135	-0.736639
5	6	0	-2.020687	0.631997	-1.354161
6	1	0	-1.171316	1.122110	1.442207
7	1	0	-3.237984	0.362287	1.194480
8	1	0	-3.934562	-0.381728	-0.954817
9	1	0	-2.560444	-1.467215	-1.129347
10	1	0	-2.515058	1.593518	-1.169376
11	1	0	-1.898458	0.533988	-2.439125
12	6	0	0.210597	-0.559757	-1.186755
13	1	0	0.425988	-0.352836	-2.239674
14	1	0	-0.354324	-1.501940	-1.171233
15	6	0	1.539292	-0.756727	-0.420904
16	1	0	2.306137	-0.117364	-0.869271
17	6	0	1.365109	-0.351530	1.068733
18	1	0	2.105945	-0.873261	1.684698
19	6	0	-0.050127	-0.681075	1.576973
20	1	0	-0.102709	-0.556445	2.664308
21	1	0	-0.210070	-1.749882	1.381478
22	6	0	-2.855690	-1.704693	1.527173
23	1	0	-2.632568	-1.623068	2.597542
24	1	0	-2.279072	-2.547122	1.126669
25	1	0	-3.916427	-1.966293	1.431967
26	6	0	0.104504	1.890378	-0.597611
27	6	0	1.116343	2.124767	0.346138
28	1	0	1.539031	3.124478	0.421672
29	6	0	1.660121	1.174574	1.200471
30	8	0	2.518894	1.369286	2.147110
31	6	0	2.056776	-2.193551	-0.525835
32	1	0	3.016028	-2.278463	0.010899
33	1	0	1.346111	-2.888904	-0.046795
34	8	0	2.214293	-2.496805	-1.906832
35	1	0	2.512167	-3.415914	-1.979820
36	6	0	-0.173472	2.935953	-1.638659
37	1	0	-0.023305	2.554761	-2.662676
38	1	0	0.491885	3.796933	-1.516291

```
    39          1          0        -1.206855     3.314016    -1.610834
 ---------------------------------------------------------------------
Excitation energies and oscillator strengths:
Excited State   1:      Singlet-A      2.7192 eV  455.95 nm  f=0.0003  <S**2>=0.000
      64 -> 65         -0.70101
Excited State   2:      Singlet-A      4.9423 eV  250.86 nm  f=0.2435  <S**2>=0.000
      61 -> 65         -0.15574
      63 -> 65          0.67496

 Excited State   3:      Singlet-A      5.0098 eV  247.48 nm  f=0.0016  <S**2>=0.000
      62 -> 65         -0.70381

 Excited State   4:      Singlet-A      5.4436 eV  227.76 nm  f=0.0060  <S**2>=0.000
      60 -> 65          0.67788
      61 -> 65         -0.17106

 Excited State   5:      Singlet-A      5.5196 eV  224.63 nm  f=0.0421  <S**2>=0.000
      60 -> 65          0.17290
      61 -> 65          0.65486
      63 -> 65          0.14377

 Excited State   6:      Singlet-A      5.7384 eV  216.06 nm  f=0.0026  <S**2>=0.000
      59 -> 65         -0.67899

 Excited State   7:      Singlet-A      6.1156 eV  202.73 nm  f=0.0043  <S**2>=0.000
      53 -> 65          0.21648
      54 -> 65         -0.18146
      55 -> 65          0.35358
      56 -> 65         -0.44040
      57 -> 65         -0.25325
```

Compound **4**:

Standard orientation:

Center Number	Atomic Number	Atomic Type	Coordinates (Angstroms) X	Y	Z
1	6	0	1.496844	1.634854	-0.042364
2	6	0	0.514064	0.739230	-0.508811
3	6	0	1.088503	-0.655556	-0.576685
4	6	0	2.636932	-0.456143	-0.489007
5	6	0	2.732956	1.020877	0.064864
6	1	0	1.358690	2.683327	0.193744
7	1	0	0.875513	-1.137725	-1.540106
8	1	0	3.044855	-0.375563	-1.506554
9	6	0	-0.955098	1.031325	-0.707959
10	6	0	-1.833337	-1.424171	-0.618398
11	6	0	-1.044120	-1.408217	0.704491
12	1	0	-1.376385	-2.272864	1.291976
13	6	0	0.489175	-1.578169	0.514828
14	1	0	0.699885	-2.623704	0.264211
15	1	0	0.957065	-1.367143	1.482529
16	6	0	3.465400	-1.483329	0.278985
17	1	0	3.198677	-1.522085	1.338687
18	1	0	4.531589	-1.250406	0.207542
19	1	0	3.305649	-2.480453	-0.147068
20	8	0	3.890052	1.411606	0.420063
21	6	0	-1.169511	2.349155	-1.479938
22	1	0	-0.668389	3.187264	-0.982845
23	1	0	-2.235960	2.585286	-1.544907
24	1	0	-0.760403	2.270287	-2.493388
25	1	0	-2.897700	-1.499339	-0.351750
26	6	0	-1.640001	-0.136568	-1.468985
27	1	0	-2.615981	0.209990	-1.820487
28	1	0	-1.043048	-0.381818	-2.355052
29	6	0	-1.625567	1.122463	0.689270
30	6	0	-1.370099	-0.145719	1.489090
31	8	0	-1.437919	-2.606393	-1.323252
32	1	0	-1.972864	-2.654823	-2.130675
33	6	0	-1.459092	-0.135569	2.824202
34	1	0	-1.629092	0.785861	3.378608
35	1	0	-1.349486	-1.041086	3.417110
36	1	0	-1.215678	1.989025	1.229593
37	8	0	-3.039901	1.308341	0.488676
38	1	0	-3.467065	1.140753	1.343437

Excitation energies and oscillator strengths:

```
Excited State   1:      Singlet-A      2.8408 eV  436.44 nm  f=0.0002  <S**2>=0.000
      67 -> 68         0.70003
Excited State   2:      Singlet-A      4.7296 eV  262.15 nm  f=0.0415  <S**2>=0.000
      65 -> 68         0.56786
      66 -> 68         0.41078

Excited State   3:      Singlet-A      5.0071 eV  247.62 nm  f=0.0495  <S**2>=0.000
      64 -> 68         0.58209
      65 -> 68        -0.23652
      66 -> 68         0.28743
      67 -> 69         0.12632

Excited State   4:      Singlet-A      5.1321 eV  241.59 nm  f=0.0592  <S**2>=0.000
      63 -> 68         0.18798
      64 -> 68        -0.31127
      65 -> 68        -0.19619
      66 -> 68         0.23399
      67 -> 69         0.51289

Excited State   5:      Singlet-A      5.1853 eV  239.11 nm  f=0.0684  <S**2>=0.000
      63 -> 68         0.49703
      65 -> 68        -0.18232
      66 -> 68         0.21321
      67 -> 69        -0.39987

Excited State   6:      Singlet-A      5.2791 eV  234.86 nm  f=0.1418  <S**2>=0.000
      63 -> 68         0.45697
      64 -> 68         0.22823
      65 -> 68         0.19824
      66 -> 68        -0.36100
      67 -> 69         0.22761

Excited State   7:      Singlet-A      5.7985 eV  213.82 nm  f=0.0182  <S**2>=0.000
      62 -> 68         0.69084

Excited State   8:      Singlet-A      5.9598 eV  208.03 nm  f=0.0050  <S**2>=0.000
      59 -> 68        -0.17656
      60 -> 68         0.19106
      61 -> 68         0.57891
      66 -> 69         0.24958

Excited State   9:      Singlet-A      6.0014 eV  206.59 nm  f=0.0197  <S**2>=0.000
      60 -> 68         0.16801
```

61 -> 68	0.20485
65 -> 69	0.17832
66 -> 69	-0.61279

Excited State 10:	Singlet-A	6.1303 eV	202.25 nm	f=0.0019	<S**2>=0.000
57 -> 68	0.16933				
58 -> 68	0.18265				
60 -> 68	-0.51924				
61 -> 68	0.10834				
67 -> 70	-0.24553				
67 -> 71	0.23862				

Compound **15**:

Calculated (5*S*,6*S*,7*S*,8*R*)-15

Optimized Parameters !

! (Angstroms and Degrees) !

! Name	Definition	Value	Derivative Info.		!
! R1	R(1,2)	1.3827	-DE/DX =	0.0	!
! R2	R(1,3)	1.3701	-DE/DX =	0.0	!
! R3	R(1,8)	1.4071	-DE/DX =	0.0	!
! R4	R(2,4)	1.4012	-DE/DX =	0.0	!
! R5	R(2,14)	1.5075	-DE/DX =	0.0	!
! R6	R(3,5)	1.3431	-DE/DX =	0.0	!
! R7	R(4,6)	1.4448	-DE/DX =	0.0	!
! R8	R(4,23)	1.277	-DE/DX =	0.0	!
! R9	R(5,6)	1.3618	-DE/DX =	0.0	!
! R10	R(5,24)	1.4984	-DE/DX =	0.0	!
! R11	R(6,7)	1.0813	-DE/DX =	0.0	!
! R12	R(8,9)	1.0808	-DE/DX =	0.0	!
! R13	R(8,10)	1.5208	-DE/DX =	0.0	!
! R14	R(8,16)	2.4767	-DE/DX =	0.0	!
! R15	R(10,11)	1.0926	-DE/DX =	0.0	!
! R16	R(10,12)	1.5508	-DE/DX =	0.0	!
! R17	R(10,17)	1.42	-DE/DX =	0.0	!
! R18	R(12,13)	1.0981	-DE/DX =	0.0	!
! R19	R(12,14)	1.5354	-DE/DX =	0.0	!
! R20	R(12,19)	1.4242	-DE/DX =	0.0	!
! R21	R(14,15)	1.0989	-DE/DX =	0.0	!
! R22	R(14,21)	1.4353	-DE/DX =	0.0	!
! R23	R(17,18)	0.9735	-DE/DX =	0.0	!
! R24	R(19,20)	0.9703	-DE/DX =	0.0	!
! R25	R(21,22)	0.9698	-DE/DX =	0.0	!
! R26	R(24,25)	1.0978	-DE/DX =	0.0	!
! R27	R(24,26)	1.0941	-DE/DX =	0.0	!
! R28	R(24,27)	1.5542	-DE/DX =	0.0	!
! R29	R(27,28)	1.0965	-DE/DX =	0.0	!
! R30	R(27,29)	1.0946	-DE/DX =	0.0	!
! R31	R(27,30)	1.5144	-DE/DX =	0.0	!
! R32	R(30,32)	1.4022	-DE/DX =	0.0	!
! R33	R(30,33)	1.4019	-DE/DX =	0.0	!
! R34	R(31,34)	1.3956	-DE/DX =	0.0	!
! R35	R(31,35)	1.3968	-DE/DX =	0.0	!

! R36	R(31,40)	1.0869	-DE/DX =	0.0	!
! R37	R(32,35)	1.3949	-DE/DX =	0.0	!
! R38	R(32,36)	1.0889	-DE/DX =	0.0	!
! R39	R(33,34)	1.3966	-DE/DX =	0.0	!
! R40	R(33,37)	1.0885	-DE/DX =	0.0	!
! R41	R(34,38)	1.0865	-DE/DX =	0.0	!
! R42	R(35,39)	1.0871	-DE/DX =	0.0	!
! A1	A(2,1,3)	119.8241	-DE/DX =	0.0	!
! A2	A(2,1,8)	122.5415	-DE/DX =	0.0	!
! A3	A(3,1,8)	117.5207	-DE/DX =	0.0	!
! A4	A(1,2,4)	120.9073	-DE/DX =	0.0	!
! A5	A(1,2,14)	118.4496	-DE/DX =	0.0	!
! A6	A(4,2,14)	120.5981	-DE/DX =	0.0	!
! A7	A(1,3,5)	120.7792	-DE/DX =	0.0	!
! A8	A(2,4,6)	116.9801	-DE/DX =	0.0	!
! A9	A(2,4,23)	123.7264	-DE/DX =	0.0	!
! A10	A(6,4,23)	119.2858	-DE/DX =	0.0	!
! A11	A(3,5,6)	122.1755	-DE/DX =	0.0	!
! A12	A(3,5,24)	112.4517	-DE/DX =	0.0	!
! A13	A(6,5,24)	125.3692	-DE/DX =	0.0	!
! A14	A(4,6,5)	119.1852	-DE/DX =	0.0	!
! A15	A(4,6,7)	119.9174	-DE/DX =	0.0	!
! A16	A(5,6,7)	120.8759	-DE/DX =	0.0	!
! A17	A(1,8,9)	119.3518	-DE/DX =	0.0	!
! A18	A(1,8,10)	119.9189	-DE/DX =	0.0	!
! A19	A(1,8,16)	95.5188	-DE/DX =	0.0	!
! A20	A(9,8,10)	116.8745	-DE/DX =	0.0	!
! A21	A(9,8,16)	93.2129	-DE/DX =	0.0	!
! A22	A(10,8,16)	100.9029	-DE/DX =	0.0	!
! A23	A(8,10,11)	108.1798	-DE/DX =	0.0	!
! A24	A(8,10,12)	113.0193	-DE/DX =	0.0	!
! A25	A(8,10,17)	111.1302	-DE/DX =	0.0	!
! A26	A(11,10,12)	108.8548	-DE/DX =	0.0	!
! A27	A(11,10,17)	106.0401	-DE/DX =	0.0	!
! A28	A(12,10,17)	109.3474	-DE/DX =	0.0	!
! A29	A(10,12,13)	109.0768	-DE/DX =	0.0	!
! A30	A(10,12,14)	111.767	-DE/DX =	0.0	!
! A31	A(10,12,19)	106.6041	-DE/DX =	0.0	!
! A32	A(13,12,14)	109.4375	-DE/DX =	0.0	!
! A33	A(13,12,19)	110.2248	-DE/DX =	0.0	!
! A34	A(14,12,19)	109.6952	-DE/DX =	0.0	!
! A35	A(2,14,12)	109.5376	-DE/DX =	0.0	!
! A36	A(2,14,15)	109.8859	-DE/DX =	0.0	!
! A37	A(2,14,21)	109.033	-DE/DX =	0.0	!

! A38	A(12,14,15)	109.3956	-DE/DX =	0.0	!
! A39	A(12,14,21)	108.4798	-DE/DX =	0.0	!
! A40	A(15,14,21)	110.4844	-DE/DX =	0.0	!
! A41	A(10,17,18)	105.9518	-DE/DX =	0.0	!
! A42	A(12,19,20)	107.7031	-DE/DX =	0.0	!
! A43	A(14,21,22)	109.3631	-DE/DX =	0.0	!
! A44	A(5,24,25)	108.3365	-DE/DX =	0.0	!
! A45	A(5,24,26)	108.6471	-DE/DX =	0.0	!
! A46	A(5,24,27)	112.7715	-DE/DX =	0.0	!
! A47	A(25,24,26)	107.7189	-DE/DX =	0.0	!
! A48	A(25,24,27)	109.0255	-DE/DX =	0.0	!
! A49	A(26,24,27)	110.2001	-DE/DX =	0.0	!
! A50	A(24,27,28)	106.8906	-DE/DX =	0.0	!
! A51	A(24,27,29)	108.6275	-DE/DX =	0.0	!
! A52	A(24,27,30)	113.5788	-DE/DX =	0.0	!
! A53	A(28,27,29)	107.01	-DE/DX =	0.0	!
! A54	A(28,27,30)	110.2335	-DE/DX =	0.0	!
! A55	A(29,27,30)	110.2399	-DE/DX =	0.0	!
! A56	A(27,30,32)	120.3867	-DE/DX =	0.0	!
! A57	A(27,30,33)	120.7954	-DE/DX =	0.0	!
! A58	A(32,30,33)	118.806	-DE/DX =	0.0	!
! A59	A(34,31,35)	119.6578	-DE/DX =	0.0	!
! A60	A(34,31,40)	120.2362	-DE/DX =	0.0	!
! A61	A(35,31,40)	120.1048	-DE/DX =	0.0	!
! A62	A(30,32,35)	120.825	-DE/DX =	0.0	!
! A63	A(30,32,36)	119.5587	-DE/DX =	0.0	!
! A64	A(35,32,36)	119.6128	-DE/DX =	0.0	!
! A65	A(30,33,34)	120.388	-DE/DX =	0.0	!
! A66	A(30,33,37)	120.404	-DE/DX =	0.0	!
! A67	A(34,33,37)	119.2078	-DE/DX =	0.0	!
! A68	A(31,34,33)	120.357	-DE/DX =	0.0	!
! A69	A(31,34,38)	120.3628	-DE/DX =	0.0	!
! A70	A(33,34,38)	119.2797	-DE/DX =	0.0	!
! A71	A(31,35,32)	119.966	-DE/DX =	0.0	!
! A72	A(31,35,39)	120.1802	-DE/DX =	0.0	!
! A73	A(32,35,39)	119.8519	-DE/DX =	0.0	!
! D1	D(3,1,2,4)	-3.1983	-DE/DX =	0.0	!
! D2	D(3,1,2,14)	174.378	-DE/DX =	0.0	!
! D3	D(8,1,2,4)	-179.2235	-DE/DX =	0.0	!
! D4	D(8,1,2,14)	-1.6472	-DE/DX =	0.0	!
! D5	D(2,1,3,5)	-0.2799	-DE/DX =	0.0	!
! D6	D(8,1,3,5)	175.942	-DE/DX =	0.0	!
! D7	D(2,1,8,9)	-177.0249	-DE/DX =	0.0	!
! D8	D(2,1,8,10)	-19.8084	-DE/DX =	0.0	!

! D9	D(2,1,8,16)	86.1444	-DE/DX =	0.0	!
! D10	D(3,1,8,9)	6.8633	-DE/DX =	0.0	!
! D11	D(3,1,8,10)	164.0799	-DE/DX =	0.0	!
! D12	D(3,1,8,16)	-89.9674	-DE/DX =	0.0	!
! D13	D(1,2,4,6)	3.9578	-DE/DX =	0.0	!
! D14	D(1,2,4,23)	-177.0613	-DE/DX =	0.0	!
! D15	D(14,2,4,6)	-173.5664	-DE/DX =	0.0	!
! D16	D(14,2,4,23)	5.4145	-DE/DX =	0.0	!
! D17	D(1,2,14,12)	39.7392	-DE/DX =	0.0	!
! D18	D(1,2,14,15)	-80.4647	-DE/DX =	0.0	!
! D19	D(1,2,14,21)	158.3072	-DE/DX =	0.0	!
! D20	D(4,2,14,12)	-142.6768	-DE/DX =	0.0	!
! D21	D(4,2,14,15)	97.1193	-DE/DX =	0.0	!
! D22	D(4,2,14,21)	-24.1087	-DE/DX =	0.0	!
! D23	D(1,3,5,6)	2.8888	-DE/DX =	0.0	!
! D24	D(1,3,5,24)	-176.4522	-DE/DX =	0.0	!
! D25	D(2,4,6,5)	-1.4353	-DE/DX =	0.0	!
! D26	D(2,4,6,7)	176.8959	-DE/DX =	0.0	!
! D27	D(23,4,6,5)	179.5365	-DE/DX =	0.0	!
! D28	D(23,4,6,7)	-2.1323	-DE/DX =	0.0	!
! D29	D(3,5,6,4)	-1.9595	-DE/DX =	0.0	!
! D30	D(3,5,6,7)	179.7258	-DE/DX =	0.0	!
! D31	D(24,5,6,4)	177.2936	-DE/DX =	0.0	!
! D32	D(24,5,6,7)	-1.0212	-DE/DX =	0.0	!
! D33	D(3,5,24,25)	-58.1678	-DE/DX =	0.0	!
! D34	D(3,5,24,26)	-174.9263	-DE/DX =	0.0	!
! D35	D(3,5,24,27)	62.6014	-DE/DX =	0.0	!
! D36	D(6,5,24,25)	122.5162	-DE/DX =	0.0	!
! D37	D(6,5,24,26)	5.7577	-DE/DX =	0.0	!
! D38	D(6,5,24,27)	-116.7145	-DE/DX =	0.0	!
! D39	D(1,8,10,11)	120.9322	-DE/DX =	0.0	!
! D40	D(1,8,10,12)	0.328	-DE/DX =	0.0	!
! D41	D(1,8,10,17)	-123.0464	-DE/DX =	0.0	!
! D42	D(9,8,10,11)	-81.3028	-DE/DX =	0.0	!
! D43	D(9,8,10,12)	158.093	-DE/DX =	0.0	!
! D44	D(9,8,10,17)	34.7187	-DE/DX =	0.0	!
! D45	D(16,8,10,11)	17.9972	-DE/DX =	0.0	!
! D46	D(16,8,10,12)	-102.607	-DE/DX =	0.0	!
! D47	D(16,8,10,17)	134.0186	-DE/DX =	0.0	!
! D48	D(8,10,12,13)	158.4322	-DE/DX =	0.0	!
! D49	D(8,10,12,14)	37.285	-DE/DX =	0.0	!
! D50	D(8,10,12,19)	-82.5662	-DE/DX =	0.0	!
! D51	D(11,10,12,13)	38.2132	-DE/DX =	0.0	!
! D52	D(11,10,12,14)	-82.934	-DE/DX =	0.0	!

! D53	D(11,10,12,19)	157.2147	-DE/DX =	0.0	!
! D54	D(17,10,12,13)	-77.2138	-DE/DX =	0.0	!
! D55	D(17,10,12,14)	161.6389	-DE/DX =	0.0	!
! D56	D(17,10,12,19)	41.7877	-DE/DX =	0.0	!
! D57	D(8,10,17,18)	83.5101	-DE/DX =	0.0	!
! D58	D(11,10,17,18)	-159.1575	-DE/DX =	0.0	!
! D59	D(12,10,17,18)	-41.942	-DE/DX =	0.0	!
! D60	D(10,12,14,2)	-56.488	-DE/DX =	0.0	!
! D61	D(10,12,14,15)	64.0147	-DE/DX =	0.0	!
! D62	D(10,12,14,21)	-175.3986	-DE/DX =	0.0	!
! D63	D(13,12,14,2)	-177.4259	-DE/DX =	0.0	!
! D64	D(13,12,14,15)	-56.9232	-DE/DX =	0.0	!
! D65	D(13,12,14,21)	63.6635	-DE/DX =	0.0	!
! D66	D(19,12,14,2)	61.5299	-DE/DX =	0.0	!
! D67	D(19,12,14,15)	-177.9673	-DE/DX =	0.0	!
! D68	D(19,12,14,21)	-57.3807	-DE/DX =	0.0	!
! D69	D(10,12,19,20)	179.5968	-DE/DX =	0.0	!
! D70	D(13,12,19,20)	-62.1528	-DE/DX =	0.0	!
! D71	D(14,12,19,20)	58.4152	-DE/DX =	0.0	!
! D72	D(2,14,21,22)	98.7594	-DE/DX =	0.0	!
! D73	D(12,14,21,22)	-142.0124	-DE/DX =	0.0	!
! D74	D(15,14,21,22)	-22.1032	-DE/DX =	0.0	!
! D75	D(5,24,27,28)	-175.0216	-DE/DX =	0.0	!
! D76	D(5,24,27,29)	-59.8723	-DE/DX =	0.0	!
! D77	D(5,24,27,30)	63.1829	-DE/DX =	0.0	!
! D78	D(25,24,27,28)	-54.6467	-DE/DX =	0.0	!
! D79	D(25,24,27,29)	60.5026	-DE/DX =	0.0	!
! D80	D(25,24,27,30)	-176.4422	-DE/DX =	0.0	!
! D81	D(26,24,27,28)	63.3814	-DE/DX =	0.0	!
! D82	D(26,24,27,29)	178.5307	-DE/DX =	0.0	!
! D83	D(26,24,27,30)	-58.4141	-DE/DX =	0.0	!
! D84	D(24,27,30,32)	82.0634	-DE/DX =	0.0	!
! D85	D(24,27,30,33)	-96.6693	-DE/DX =	0.0	!
! D86	D(28,27,30,32)	-37.8529	-DE/DX =	0.0	!
! D87	D(28,27,30,33)	143.4145	-DE/DX =	0.0	!
! D88	D(29,27,30,32)	-155.7706	-DE/DX =	0.0	!
! D89	D(29,27,30,33)	25.4967	-DE/DX =	0.0	!
! D90	D(27,30,32,35)	-178.6227	-DE/DX =	0.0	!
! D91	D(27,30,32,36)	2.0651	-DE/DX =	0.0	!
! D92	D(33,30,32,35)	0.1349	-DE/DX =	0.0	!
! D93	D(33,30,32,36)	-179.1773	-DE/DX =	0.0	!
! D94	D(27,30,33,34)	178.6318	-DE/DX =	0.0	!
! D95	D(27,30,33,37)	-1.5357	-DE/DX =	0.0	!
! D96	D(32,30,33,34)	-0.1206	-DE/DX =	0.0	!

```
! D97    D(32,30,33,37)        179.7119        -DE/DX =    0.0              !
! D98    D(35,31,34,33)          0.0826        -DE/DX =    0.0              !
! D99    D(35,31,34,38)       -179.6509        -DE/DX =    0.0              !
! D100   D(40,31,34,33)        179.6905        -DE/DX =    0.0              !
! D101   D(40,31,34,38)         -0.043         -DE/DX =    0.0              !
! D102   D(34,31,35,32)         -0.0686        -DE/DX =    0.0              !
! D103   D(34,31,35,39)        179.4327        -DE/DX =    0.0              !
! D104   D(40,31,35,32)       -179.6771        -DE/DX =    0.0              !
! D105   D(40,31,35,39)         -0.1758        -DE/DX =    0.0              !
! D106   D(30,32,35,31)         -0.0411        -DE/DX =    0.0              !
! D107   D(30,32,35,39)       -179.544         -DE/DX =    0.0              !
! D108   D(36,32,35,31)        179.2708        -DE/DX =    0.0              !
! D109   D(36,32,35,39)         -0.2321        -DE/DX =    0.0              !
! D110   D(30,33,34,31)          0.013         -DE/DX =    0.0              !
! D111   D(30,33,34,38)        179.7494        -DE/DX =    0.0              !
! D112   D(37,33,34,31)       -179.8215        -DE/DX =    0.0              !
! D113   D(37,33,34,38)         -0.0851        -DE/DX =    0.0              !
Excitation energies and oscillator strengths:

 Excited State   1:      Singlet-A      1.3504 eV  918.10 nm  f=0.0002
<S**2>=0.000
      88 -> 89         0.70228
 This state for optimization and/or second-order correction.
 Total Energy, E(TD-HF/TD-KS) =  -1494.21926985
 Copying the excited state density for this state as the 1-particle RhoCI density.

 Excited State   2:      Singlet-A      2.1303 eV  582.01 nm  f=0.0001
<S**2>=0.000
      87 -> 89         0.70049

 Excited State   3:      Singlet-A      2.2472 eV  551.74 nm  f=0.0013
<S**2>=0.000
      84 -> 89        -0.12574
      85 -> 89         0.11544
      86 -> 89        -0.67694

 Excited State   4:      Singlet-A      2.3550 eV  526.47 nm  f=0.0097
<S**2>=0.000
      82 -> 89         0.35492
      83 -> 89        -0.23373
      84 -> 89         0.42591
      85 -> 89        -0.32247
      86 -> 89        -0.17718
```

```
 Excited State   5:      Singlet-A      2.3912 eV  518.49 nm  f=0.0009
<S**2>=0.000
      82 -> 89         0.10140
      83 -> 89         0.62592
      84 -> 89         0.29617

 Excited State   6:      Singlet-A      2.5295 eV  490.15 nm  f=0.0239
<S**2>=0.000
      83 -> 89        -0.22828
      84 -> 89         0.33812
      85 -> 89         0.57245

 Excited State   7:      Singlet-A      3.3115 eV  374.41 nm  f=0.0025
<S**2>=0.000
      81 -> 89         0.69854

 Excited State   8:      Singlet-A      3.3316 eV  372.15 nm  f=0.0431
<S**2>=0.000
      80 -> 89        -0.56217
      82 -> 89         0.36116
      84 -> 89        -0.16056
      85 -> 89         0.11119

 Excited State   9:      Singlet-A      3.6297 eV  341.58 nm  f=0.1836
<S**2>=0.000
      76 -> 89        -0.11552
      78 -> 89        -0.10390
      80 -> 89        -0.41058
      82 -> 89        -0.45177
      84 -> 89         0.26341
      85 -> 89        -0.16977

 Excited State  10:      Singlet-A      3.8443 eV  322.51 nm  f=0.0077
<S**2>=0.000
      79 -> 89        -0.42684
      88 -> 90         0.53799

 Excited State  11:      Singlet-A      3.9006 eV  317.86 nm  f=0.0087
<S**2>=0.000
      79 -> 89         0.55724
      88 -> 90         0.42237

 Excited State  12:      Singlet-A      4.6311 eV  267.72 nm  f=0.0097
<S**2>=0.000
```

```
      76 -> 89         -0.24746
      77 -> 89          0.13239
      78 -> 89          0.64072

 Excited State  13:      Singlet-A      4.6932 eV  264.18 nm  f=0.0016
<S**2>=0.000
      74 -> 89          0.11048
      77 -> 89          0.68306
      78 -> 89         -0.12510

 Excited State  14:      Singlet-A      4.7689 eV  259.99 nm  f=0.0083
<S**2>=0.000
      74 -> 89          0.59863
      75 -> 89          0.23686
      76 -> 89         -0.21036
      77 -> 89         -0.11595

 Excited State  15:      Singlet-A      4.8370 eV  256.33 nm  f=0.0032
<S**2>=0.000
      87 -> 90          0.69499

 Excited State  16:      Singlet-A      4.9203 eV  251.98 nm  f=0.0019
<S**2>=0.000
      88 -> 91         -0.68885

 Excited State  17:      Singlet-A      5.0079 eV  247.58 nm  f=0.0048
<S**2>=0.000
      76 -> 89         -0.17040
      85 -> 90          0.13876
      86 -> 90         -0.65248

 Excited State  18:      Singlet-A      5.0304 eV  246.47 nm  f=0.0736
<S**2>=0.000
      72 -> 89         -0.19295
      73 -> 89          0.15628
      74 -> 89         -0.23143
      76 -> 89         -0.50345
      78 -> 89         -0.17364
      82 -> 89          0.11555
      86 -> 90          0.20334

 Excited State  19:      Singlet-A      5.1030 eV  242.96 nm  f=0.0013
<S**2>=0.000
      71 -> 89         -0.11674
```

72 -> 89 0.14789
74 -> 89 -0.24855
75 -> 89 0.62205

Excited State 20: Singlet-A 5.2259 eV 237.25 nm f=0.0501
<S**2>=0.000
69 -> 89 0.18516
73 -> 89 0.30727
76 -> 89 0.12862
82 -> 90 -0.10080
84 -> 90 0.15619
85 -> 90 -0.51254
85 -> 91 -0.14998
86 -> 90 -0.10440

Calculated (5*R*,6*R*,7*R*,8*S*)-15

R1 2.61288 0.00000 -0.00004 0.00005 0.00001 2.61289
R2 2.58917 -0.00004 -0.00001 -0.00003 -0.00004
2.58913
R3 2.65899 -0.00001 0.00008 -0.00006 0.00002
2.65901
R4 2.64786 0.00001 0.00008 0.00002 0.00009
2.64795
R5 2.84904 -0.00003 -0.00014 -0.00006 -0.00019
2.84885
R6 2.53796 0.00001 0.00005 -0.00002 0.00003
2.53799
R7 2.73038 -0.00002 -0.00001 -0.00001 -0.00002
2.73036
R8 2.41311 -0.00005 0.00001 -0.00005 -0.00004
2.41308
R9 2.57352 0.00000 -0.00004 0.00001 -0.00003
2.57349
R10 2.83144 0.00002 0.00005 0.00003 0.00008
2.83152
R11 2.04344 0.00000 0.00000 0.00000 0.00000
2.04344
R12 2.04249 -0.00001 0.00001 -0.00004 -0.00002
2.04247
R13 2.87419 -0.00005 -0.00012 -0.00008 -0.00019
2.87400
R14 4.68244 -0.00005 -0.00043 -0.00088 -0.00131
4.68113
R15 2.06463 0.00002 0.00002 0.00001 0.00004
2.06467

R16	2.93059	0.00003	0.00005	0.00002	0.00007	2.93066
R17	2.68315	0.00004	0.00006	0.00006	0.00012	2.68327
R18	2.07521	-0.00001	-0.00005	0.00001	-0.00004	2.07517
R19	2.90174	-0.00004	-0.00003	-0.00006	-0.00009	2.90165
R20	2.69147	0.00000	-0.00002	0.00003	0.00001	2.69149
R21	2.07651	0.00002	-0.00001	0.00004	0.00003	2.07655
R22	2.71197	0.00008	0.00013	0.00016	0.00029	2.71226
R23	1.83959	-0.00001	0.00000	-0.00001	-0.00001	1.83957
R24	1.83371	-0.00001	0.00001	-0.00001	0.00000	1.83371
R25	1.83261	-0.00001	0.00001	-0.00005	-0.00003	1.83258
R26	2.07453	0.00000	0.00003	0.00000	0.00003	2.07457
R27	2.06752	0.00000	0.00000	0.00000	0.00000	2.06753
R28	2.93714	-0.00002	-0.00006	-0.00008	-0.00014	2.93700
R29	2.07214	0.00000	-0.00001	0.00002	0.00001	2.07216
R30	2.06845	0.00000	0.00001	-0.00001	0.00000	2.06845
R31	2.86172	0.00002	0.00005	0.00004	0.00009	2.86181
R32	2.64970	0.00000	0.00000	-0.00001	-0.00001	2.64969
R33	2.64920	0.00000	-0.00001	0.00001	0.00000	2.64920
R34	2.63729	0.00001	0.00000	0.00001	0.00001	2.63730
R35	2.63953	0.00000	-0.00001	0.00000	0.00000	2.63953
R36	2.05391	0.00000	0.00000	0.00000	0.00000	2.05391
R37	2.63596	0.00001	0.00001	0.00001	0.00001	2.63597

R38	2.05774	0.00000	0.00000	0.00000	0.00000	2.05774
R39	2.63917	0.00000	0.00000	-0.00001	0.00000	2.63917
R40	2.05693	0.00000	0.00001	-0.00001	0.00000	2.05693
R41	2.05324	0.00000	0.00000	0.00000	0.00000	2.05324
R42	2.05425	0.00000	0.00000	0.00000	0.00000	2.05425
A1	2.09124	0.00001	0.00012	-0.00004	0.00008	2.09132
A2	2.13881	0.00001	-0.00022	0.00014	-0.00009	2.13873
A3	2.05118	-0.00002	0.00010	-0.00012	-0.00002	2.05116
A4	2.11030	-0.00001	0.00001	-0.00006	-0.00005	2.11025
A5	2.06743	-0.00001	-0.00003	0.00000	-0.00003	2.06740
A6	2.10472	0.00002	0.00000	0.00005	0.00005	2.10477
A7	2.10804	0.00000	-0.00016	0.00009	-0.00007	2.10797
A8	2.04162	0.00001	-0.00006	0.00009	0.00003	2.04165
A9	2.15960	-0.00002	-0.00016	0.00004	-0.00012	2.15948
A10	2.08182	0.00001	0.00022	-0.00013	0.00009	2.08192
A11	2.13232	0.00000	0.00010	-0.00003	0.00007	2.13239
A12	1.96265	0.00000	-0.00002	0.00002	0.00000	1.96265
A13	2.18815	0.00000	-0.00008	0.00001	-0.00007	2.18808
A14	2.08020	-0.00001	0.00003	-0.00005	-0.00002	2.08017
A15	2.09301	-0.00001	0.00000	-0.00004	-0.00004	2.09298
A16	2.10960	0.00001	-0.00003	0.00010	0.00006	2.10966
A17	2.08292	0.00002	0.00003	0.00006	0.00009	2.08302

A18	2.09310	-0.00002	0.00006	-0.00009	-0.00004	2.09306
A19	1.66609	0.00000	0.00029	0.00035	0.00064	1.66673
A20	2.03995	0.00000	-0.00001	-0.00004	-0.00005	2.03990
A21	1.62864	-0.00004	-0.00089	-0.00033	-0.00122	1.62742
A22	1.76022	0.00003	0.00036	0.00017	0.00053	1.76075
A23	1.88799	0.00000	-0.00003	0.00003	0.00001	1.88800
A24	1.97241	0.00001	0.00008	0.00005	0.00013	1.97254
A25	1.93967	-0.00002	-0.00001	-0.00002	-0.00003	1.93964
A26	1.89999	-0.00001	-0.00004	-0.00001	-0.00005	1.89994
A27	1.85089	-0.00001	-0.00003	-0.00013	-0.00016	1.85073
A28	1.90841	0.00002	0.00002	0.00006	0.00008	1.90849
A29	1.90374	0.00000	0.00001	-0.00005	-0.00004	1.90370
A30	1.95095	-0.00001	-0.00033	0.00011	-0.00022	1.95073
A31	1.86049	0.00002	0.00005	0.00013	0.00017	1.86066
A32	1.90997	0.00000	0.00020	-0.00014	0.00006	1.91004
A33	1.92383	-0.00001	0.00000	-0.00008	-0.00007	1.92376
A34	1.91444	0.00000	0.00006	0.00004	0.00010	1.91454
A35	1.91164	0.00001	0.00001	0.00011	0.00013	1.91177
A36	1.91781	0.00000	-0.00001	0.00004	0.00003	1.91783
A37	1.90313	0.00000	-0.00015	-0.00003	-0.00018	1.90295
A38	1.90945	-0.00001	-0.00006	-0.00003	-0.00009	1.90936
A39	1.89305	0.00001	0.00016	0.00003	0.00019	1.89324

A40	1.92852	-0.00001	0.00005	-0.00012	-0.00007	1.92845
A41	1.84943	-0.00004	0.00000	-0.00016	-0.00016	1.84927
A42	1.87968	0.00002	-0.00004	0.00013	0.00009	1.87977
A43	1.90889	0.00000	-0.00005	0.00001	-0.00004	1.90885
A44	1.89092	0.00000	0.00000	-0.00004	-0.00004	1.89088
A45	1.89635	-0.00001	0.00001	-0.00006	-0.00006	1.89630
A46	1.96816	-0.00001	0.00013	-0.00007	0.00006	1.96822
A47	1.88012	0.00000	-0.00011	0.00002	-0.00008	1.88004
A48	1.90276	0.00000	0.00005	0.00003	0.00008	1.90284
A49	1.92327	0.00001	-0.00009	0.00012	0.00002	1.92329
A50	1.86553	0.00000	0.00004	0.00001	0.00005	1.86558
A51	1.89579	0.00000	0.00014	-0.00003	0.00012	1.89591
A52	1.98226	0.00002	-0.00006	0.00005	-0.00001	1.98226
A53	1.86775	0.00000	-0.00008	0.00001	-0.00006	1.86768
A54	1.92406	-0.00001	-0.00004	-0.00003	-0.00007	1.92399
A55	1.92410	-0.00001	-0.00001	-0.00002	-0.00002	1.92407
A56	2.10118	0.00000	-0.00002	-0.00001	-0.00003	2.10115
A57	2.10826	0.00000	0.00002	0.00000	0.00002	2.10828
A58	2.07353	0.00000	0.00000	0.00001	0.00001	2.07354
A59	2.08842	0.00000	0.00001	0.00000	0.00000	2.08843
A60	2.09852	0.00000	0.00001	-0.00001	0.00000	2.09852
A61	2.09622	0.00000	-0.00002	0.00001	-0.00001	2.09622

A62	2.10880	0.00000	-0.00001	0.00000	0.00000	2.10879
A63	2.08667	0.00000	0.00003	-0.00001	0.00002	2.08669
A64	2.08765	0.00000	-0.00002	0.00000	-0.00002	2.08764
A65	2.10119	0.00000	0.00001	-0.00002	-0.00001	2.10118
A66	2.10143	0.00000	0.00000	0.00002	0.00002	2.10145
A67	2.08056	0.00000	-0.00002	0.00000	-0.00002	2.08055
A68	2.10061	0.00000	-0.00002	0.00002	0.00000	2.10062
A69	2.10074	0.00000	0.00000	0.00000	0.00000	2.10074
A70	2.08182	0.00000	0.00001	-0.00001	0.00000	2.08182
A71	2.09381	0.00000	0.00001	-0.00001	-0.00001	2.09381
A72	2.09753	0.00000	-0.00001	0.00001	0.00000	2.09753
A73	2.09181	0.00000	0.00001	0.00000	0.00001	2.09181
D1	-0.05692	0.00001	0.00021	0.00035	0.00056	-0.05635
D2	3.04369	0.00000	-0.00036	0.00009	-0.00027	3.04342
D3	-3.12964	0.00003	0.00031	0.00066	0.00096	-3.12868
D4	-0.02903	0.00001	-0.00026	0.00040	0.00013	-0.02890
D5	-0.00437	-0.00001	0.00020	-0.00043	-0.00023	-0.00459
D6	3.07177	-0.00002	0.00010	-0.00071	-0.00061	3.07116
D7	-3.08940	-0.00002	-0.00040	0.00014	-0.00026	-3.08966
D8	-0.34525	-0.00001	-0.00016	-0.00009	-0.00025	-0.34549
D9	1.50234	0.00002	0.00046	0.00031	0.00077	1.50312
D10	0.11957	-0.00001	-0.00031	0.00044	0.00013	0.11970

D11	2.86372	0.00000	-0.00007	0.00021	0.00014	2.86386
D12	-1.57187	0.00003	0.00055	0.00060	0.00116	-1.57072
D13	0.07012	-0.00001	-0.00051	-0.00009	-0.00060	0.06952
D14	-3.08959	-0.00001	-0.00049	-0.00022	-0.00071	-3.09030
D15	-3.02961	0.00000	0.00008	0.00017	0.00025	-3.02936
D16	0.09386	0.00001	0.00010	0.00005	0.00014	0.09401
D17	0.69343	0.00001	0.00063	-0.00051	0.00012	0.69355
D18	-1.40457	0.00001	0.00070	-0.00056	0.00014	-1.40443
D19	2.76249	0.00002	0.00074	-0.00042	0.00032	2.76281
D20	-2.48901	-0.00001	0.00006	-0.00077	-0.00071	-2.48972
D21	1.69618	0.00000	0.00013	-0.00082	-0.00069	1.69549
D22	-0.41995	0.00000	0.00018	-0.00068	-0.00051	-0.42046
D23	0.05049	0.00000	-0.00030	0.00026	-0.00004	0.05045
D24	-3.07935	0.00000	-0.00043	0.00015	-0.00028	-3.07963
D25	-0.02550	0.00000	0.00041	-0.00008	0.00033	-0.02517
D26	3.08682	0.00000	0.00038	0.00000	0.00038	3.08720
D27	3.13337	0.00000	0.00039	0.00004	0.00043	3.13380
D28	-0.03750	0.00000	0.00036	0.00012	0.00048	-0.03702
D29	-0.03429	0.00000	-0.00002	0.00000	-0.00002	-0.03431
D30	3.13686	0.00000	0.00001	-0.00008	-0.00007	3.13679
D31	3.09397	0.00000	0.00013	0.00012	0.00025	3.09423
D32	-0.01806	0.00000	0.00016	0.00004	0.00020	-0.01786

D33	-1.01479	0.00000	-0.00085	0.00019	-0.00067	-1.01545
D34	-3.05280	0.00000	-0.00073	0.00021	-0.00052	-3.05332
D35	1.09292	0.00000	-0.00071	0.00016	-0.00055	1.09237
D36	2.13901	0.00000	-0.00099	0.00008	-0.00092	2.13809
D37	0.10100	0.00000	-0.00087	0.00010	-0.00077	0.10023
D38	-2.03647	0.00000	-0.00085	0.00005	-0.00080	-2.03727
D39	2.11032	0.00001	0.00022	-0.00001	0.00020	2.11052
D40	0.00540	0.00001	0.00024	-0.00006	0.00018	0.00558
D41	-2.14776	-0.00001	0.00016	-0.00016	0.00000	-2.14776
D42	-1.41920	0.00002	0.00046	-0.00021	0.00025	-1.41896
D43	2.75906	0.00002	0.00048	-0.00026	0.00022	2.75928
D44	0.60591	0.00000	0.00040	-0.00036	0.00004	0.60595
D45	0.31550	-0.00001	-0.00037	-0.00051	-0.00089	0.31461
D46	-1.78942	-0.00001	-0.00035	-0.00056	-0.00091	-1.79033
D47	2.34061	-0.00003	-0.00043	-0.00066	-0.00109	2.33952
D48	2.76533	0.00000	0.00004	-0.00022	-0.00018	2.76515
D49	0.65085	0.00000	-0.00001	-0.00008	-0.00009	0.65076
D50	-1.44090	0.00000	0.00007	-0.00027	-0.00020	-1.44109
D51	0.66726	-0.00001	0.00005	-0.00029	-0.00024	0.66702
D52	-1.44722	0.00000	0.00000	-0.00015	-0.00015	-1.44737
D53	2.74422	-0.00001	0.00009	-0.00034	-0.00026	2.74396
D54	-1.34752	0.00000	0.00010	-0.00017	-0.00007	-1.34758

D55	2.82119	0.00000	0.00005	-0.00003	0.00002	2.82121
D56	0.72944	0.00000	0.00014	-0.00022	-0.00008	0.72936
D57	1.45646	0.00002	-0.00017	0.00055	0.00037	1.45683
D58	-2.77889	0.00001	-0.00023	0.00050	0.00027	-2.77862
D59	-0.73291	0.00000	-0.00028	0.00045	0.00017	-0.73275
D60	-0.98574	-0.00001	-0.00041	0.00034	-0.00006	-0.98580
D61	1.11735	0.00000	-0.00045	0.00044	-0.00001	1.11734
D62	-3.06104	-0.00001	-0.00033	0.00030	-0.00003	-3.06107
D63	-3.09660	0.00000	-0.00034	0.00043	0.00009	-3.09651
D64	-0.99352	0.00000	-0.00039	0.00053	0.00014	-0.99338
D65	1.11128	-0.00001	-0.00027	0.00039	0.00012	1.11140
D66	1.07402	0.00001	-0.00051	0.00059	0.00008	1.07410
D67	-3.10608	0.00001	-0.00056	0.00069	0.00013	-3.10595
D68	-1.00129	0.00000	-0.00044	0.00055	0.00011	-1.00118
D69	3.13249	0.00001	0.00030	0.00041	0.00071	3.13320
D70	-1.08688	0.00002	0.00035	0.00038	0.00072	-1.08616
D71	1.01730	0.00002	0.00064	0.00018	0.00082	1.01812
D72	1.72044	0.00000	0.00107	0.00171	0.00279	1.72323
D73	-2.48208	0.00001	0.00110	0.00185	0.00294	-2.47913
D74	-0.38915	0.00000	0.00115	0.00176	0.00291	-0.38623
D75	-3.05453	0.00000	-0.00011	-0.00006	-0.00017	-3.05470
D76	-1.04480	-0.00001	-0.00011	-0.00005	-0.00016	-1.04496

D77 1.10285 0.00000 -0.00006 -0.00005 -0.00011
1.10274
D78 -0.95359 0.00000 0.00001 -0.00013 -0.00012
-0.95372
D79 1.05613 0.00000 0.00001 -0.00012 -0.00011
1.05602
D80 -3.07940 0.00000 0.00006 -0.00012 -0.00006
-3.07946
D81 1.10637 0.00000 -0.00015 -0.00001 -0.00016
1.10621
D82 3.11609 0.00000 -0.00014 0.00000 -0.00015
3.11594
D83 -1.01944 0.00000 -0.00009 -0.00001 -0.00010
-1.01954
D84 1.43268 0.00000 -0.00012 -0.00001 -0.00013
1.43255
D85 -1.68674 0.00000 -0.00012 -0.00006 -0.00018
-1.68691
D86 -0.66022 -0.00001 -0.00011 -0.00004 -0.00014
-0.66036
D87 2.50355 -0.00001 -0.00010 -0.00008 -0.00018
2.50337
D88 -2.71846 0.00000 0.00002 -0.00002 0.00000
-2.71847
D89 0.44530 0.00000 0.00002 -0.00007 -0.00005
0.44526
D90 -3.11751 0.00000 0.00007 -0.00008 -0.00001
-3.11753
D91 0.03610 0.00000 0.00003 -0.00004 -0.00001
0.03609
D92 0.00234 0.00000 0.00006 -0.00003 0.00003
0.00237
D93 -3.12723 0.00000 0.00003 0.00000 0.00003
-3.12720
D94 3.11768 0.00000 -0.00005 0.00004 -0.00001
3.11767
D95 -0.02685 0.00000 -0.00019 0.00017 -0.00002
-0.02687
D96 -0.00209 0.00000 -0.00005 0.00000 -0.00005
-0.00214
D97 3.13657 0.00000 -0.00019 0.00013 -0.00006
3.13651
D98 0.00144 0.00000 0.00004 -0.00004 0.00000
0.00144

D99 -3.13550 0.00000 0.00005 -0.00003 0.00002
-3.13548
D100 3.13619 0.00000 0.00003 -0.00004 -0.00001
3.13618
D101 -0.00075 0.00000 0.00003 -0.00002 0.00001
-0.00074
D102 -0.00119 0.00000 -0.00003 0.00001 -0.00002
-0.00121
D103 3.13170 0.00000 -0.00005 0.00002 -0.00003
3.13167
D104 -3.13595 0.00000 -0.00001 0.00000 -0.00001
-3.13596
D105 -0.00306 0.00000 -0.00003 0.00001 -0.00002
-0.00307
D106 -0.00072 0.00000 -0.00003 0.00003 0.00000
-0.00071
D107 -3.13364 0.00000 -0.00001 0.00002 0.00001
-3.13362
D108 3.12885 0.00000 0.00001 -0.00001 0.00000
3.12885
D109 -0.00407 0.00000 0.00003 -0.00002 0.00001
-0.00406
D110 0.00022 0.00000 -0.00001 0.00004 0.00003
0.00025
D111 3.13721 0.00000 -0.00002 0.00003 0.00001
3.13722
D112 -3.13848 0.00000 0.00013 -0.00009 0.00005
-3.13843
D113 -0.00149 0.00000 0.00013 -0.00010 0.00002
-0.00146

Excitation energies and oscillator strengths:

Excited State 1: Singlet-A 1.3493 eV 918.89 nm f=0.0002 <S**2>=0.000
88 -> 89 0.70228
This state for optimization and/or second-order correction.
Total Energy, E(TD-HF/TD-KS) = -1494.21926940
Copying the excited state density for this state as the 1-particle RhoCI density.

Excited State 2: Singlet-A 2.1279 eV 582.67 nm f=0.0001 <S**2>=0.000
87 -> 89 0.70047

Excited State 3: Singlet-A 2.2447 eV 552.34 nm f=0.0013 <S**2>=0.000
84 -> 89 -0.12745

```
      85 -> 89         0.11651
      86 -> 89         0.67630

 Excited State   4:      Singlet-A      2.3508 eV  527.41 nm  f=0.0096  <S**2>=0.000
      82 -> 89         0.35461
      83 -> 89        -0.23178
      84 -> 89         0.42724
      85 -> 89        -0.32119
      86 -> 89         0.17956

 Excited State   5:      Singlet-A      2.3871 eV  519.39 nm  f=0.0009  <S**2>=0.000
      83 -> 89         0.62752
      84 -> 89         0.29361

 Excited State   6:      Singlet-A      2.5274 eV  490.56 nm  f=0.0239  <S**2>=0.000
      83 -> 89        -0.22586
      84 -> 89         0.33899
      85 -> 89         0.57287

 Excited State   7:      Singlet-A      3.3084 eV  374.76 nm  f=0.0021  <S**2>=0.000
      81 -> 89         0.69668

 Excited State   8:      Singlet-A      3.3291 eV  372.42 nm  f=0.0435  <S**2>=0.000
      80 -> 89        -0.55974
      82 -> 89         0.36056
      84 -> 89        -0.15969
      85 -> 89         0.11157

 Excited State   9:      Singlet-A      3.6265 eV  341.88 nm  f=0.1834  <S**2>=0.000
      76 -> 89        -0.11534
      78 -> 89        -0.10421
      80 -> 89        -0.41061
      82 -> 89        -0.45231
      84 -> 89         0.26260
      85 -> 89        -0.17029

 Excited State  10:      Singlet-A      3.8433 eV  322.59 nm  f=0.0083  <S**2>=0.000
      79 -> 89        -0.44686
      88 -> 90         0.52230

 Excited State  11:      Singlet-A      3.8986 eV  318.03 nm  f=0.0083  <S**2>=0.000
      79 -> 89         0.54134
      88 -> 90         0.44182
```

```
Excited State  12:      Singlet-A      4.6290 eV  267.84 nm  f=0.0097  <S**2>=0.000
     76 -> 89        -0.24815
     77 -> 89         0.13302
     78 -> 89         0.64036

Excited State  13:      Singlet-A      4.6908 eV  264.31 nm  f=0.0017  <S**2>=0.000
     74 -> 89         0.11124
     77 -> 89         0.68282
     78 -> 89        -0.12542

Excited State  14:      Singlet-A      4.7662 eV  260.13 nm  f=0.0083  <S**2>=0.000
     74 -> 89         0.59818
     75 -> 89         0.23845
     76 -> 89        -0.21032
     77 -> 89        -0.11658

Excited State  15:      Singlet-A      4.8365 eV  256.35 nm  f=0.0032  <S**2>=0.000
     87 -> 90         0.69506

Excited State  16:      Singlet-A      4.9207 eV  251.96 nm  f=0.0019  <S**2>=0.000
     88 -> 91        -0.68905

Excited State  17:      Singlet-A      5.0072 eV  247.61 nm  f=0.0055  <S**2>=0.000
     76 -> 89        -0.18761
     85 -> 90         0.13582
     86 -> 90         0.64502

Excited State  18:      Singlet-A      5.0277 eV  246.60 nm  f=0.0731  <S**2>=0.000
     72 -> 89        -0.18968
     73 -> 89         0.15394
     74 -> 89        -0.22996
     76 -> 89        -0.49729
     78 -> 89        -0.17271
     82 -> 89         0.11455
     86 -> 90        -0.22579

Excited State  19:      Singlet-A      5.1007 eV  243.07 nm  f=0.0014  <S**2>=0.000
     71 -> 89        -0.11685
     72 -> 89         0.14850
     74 -> 89        -0.24916
     75 -> 89         0.62146

Excited State  20:      Singlet-A      5.2247 eV  237.30 nm  f=0.0495  <S**2>=0.000
     69 -> 89         0.18457
```

73 -> 89	0.31223
76 -> 89	0.12873
82 -> 90	-0.10041
84 -> 90	0.15542
85 -> 90	-0.51068
85 -> 91	-0.14859
86 -> 90	0.10379

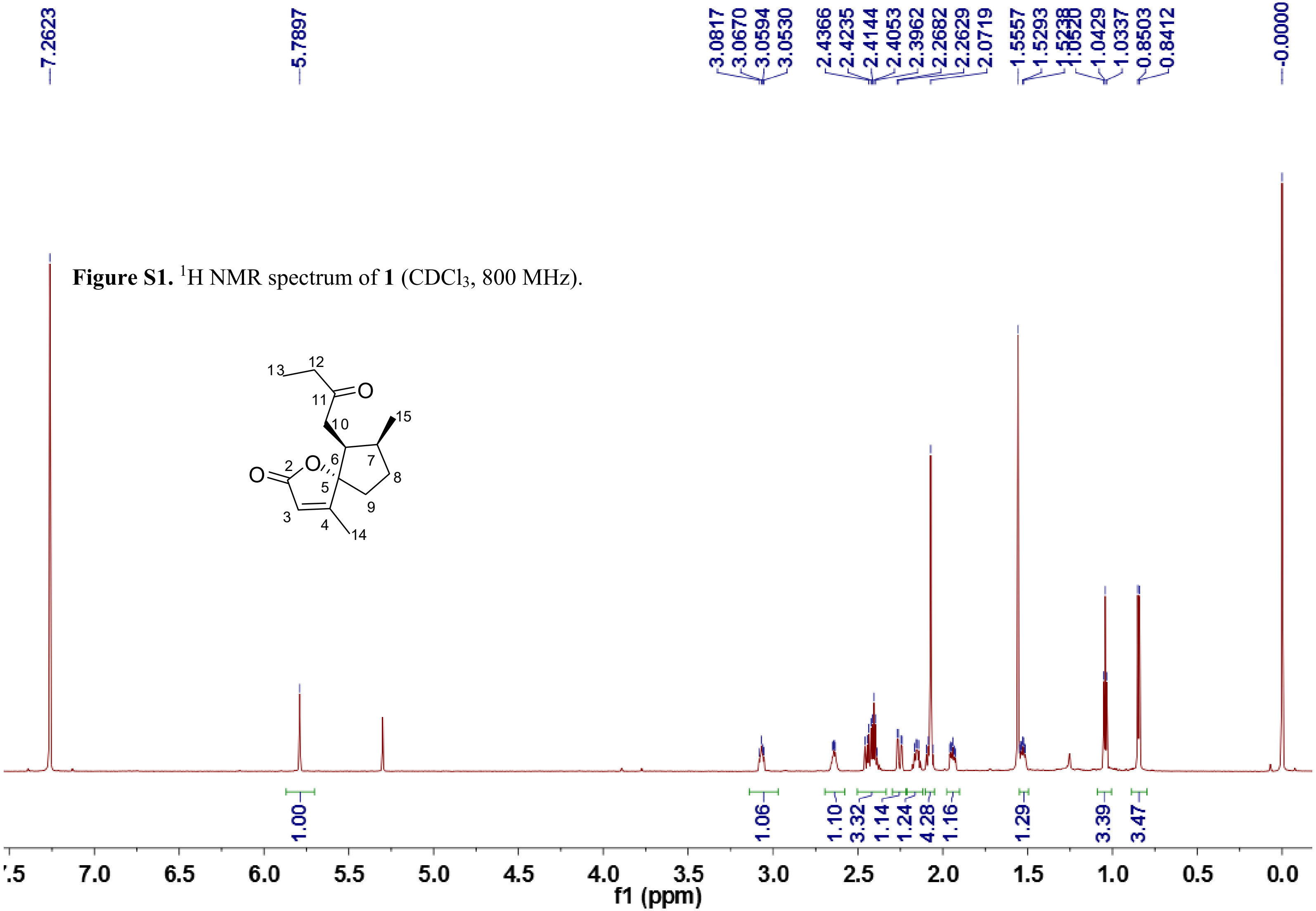

Figure S1. ^{1}H NMR spectrum of **1** ($CDCl_3$, 800 MHz).

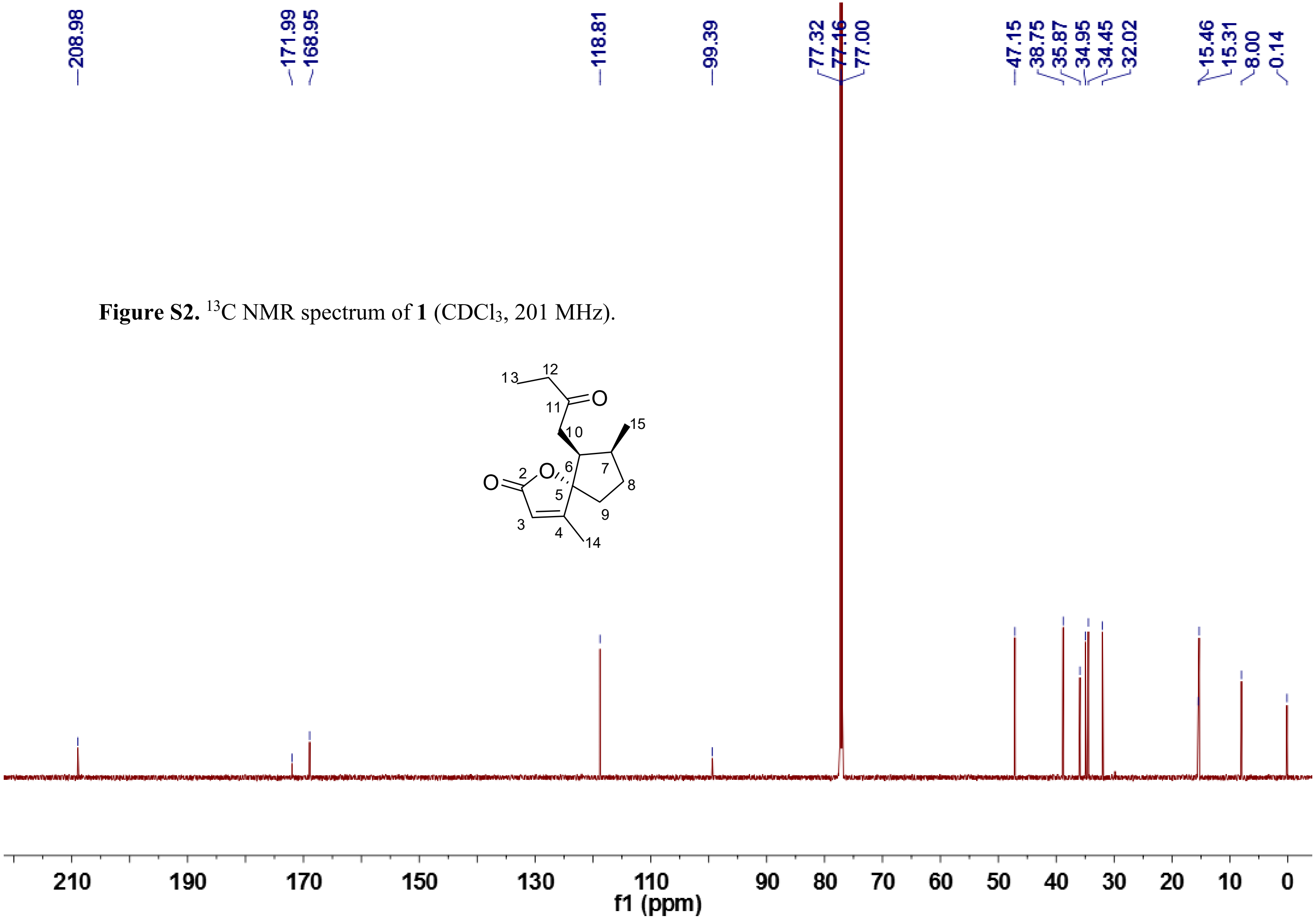

Figure S2. ^{13}C NMR spectrum of **1** ($CDCl_3$, 201 MHz).

Figure S3. HSQC spectrum of **1**.

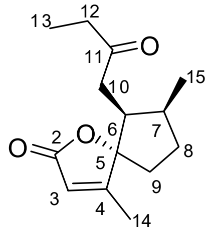

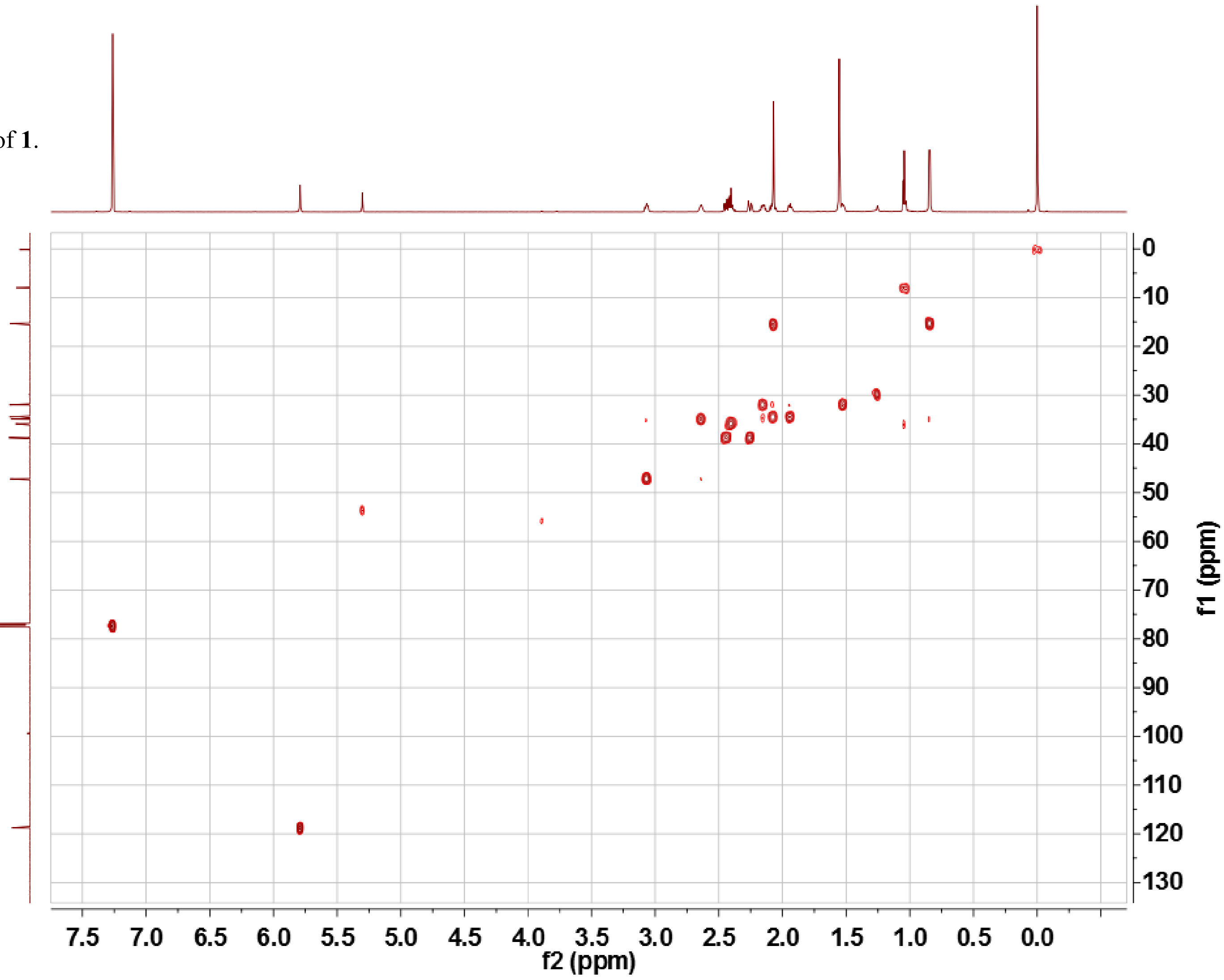

Figure S4. ^{1}H–^{1}H COSY spectrum of **1**.

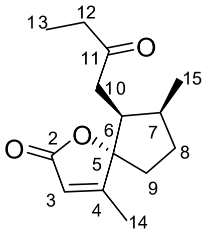

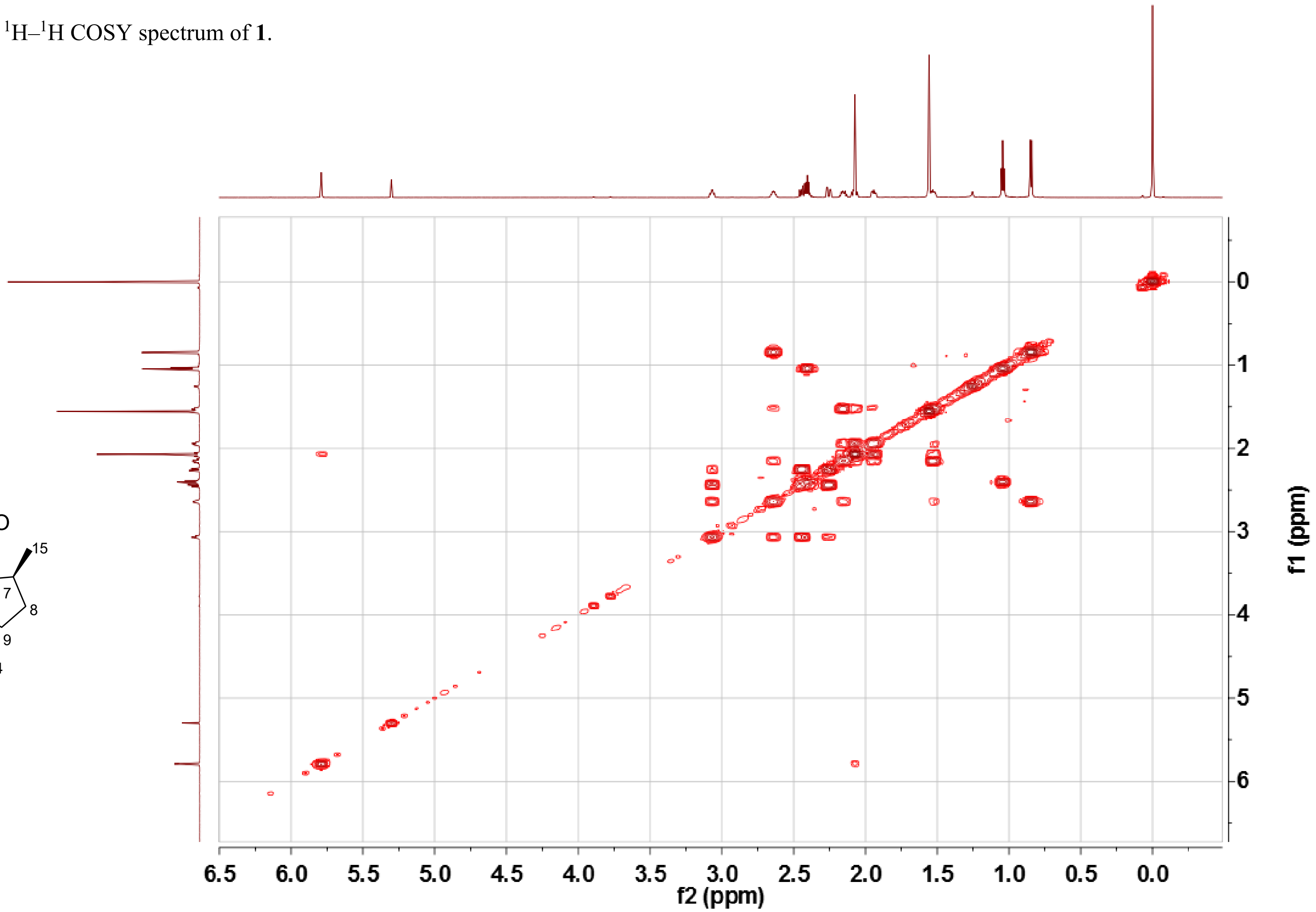

Figure S5. HMBC spectrum of **1**.

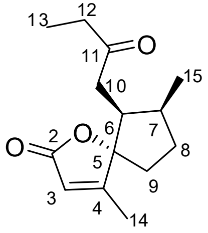

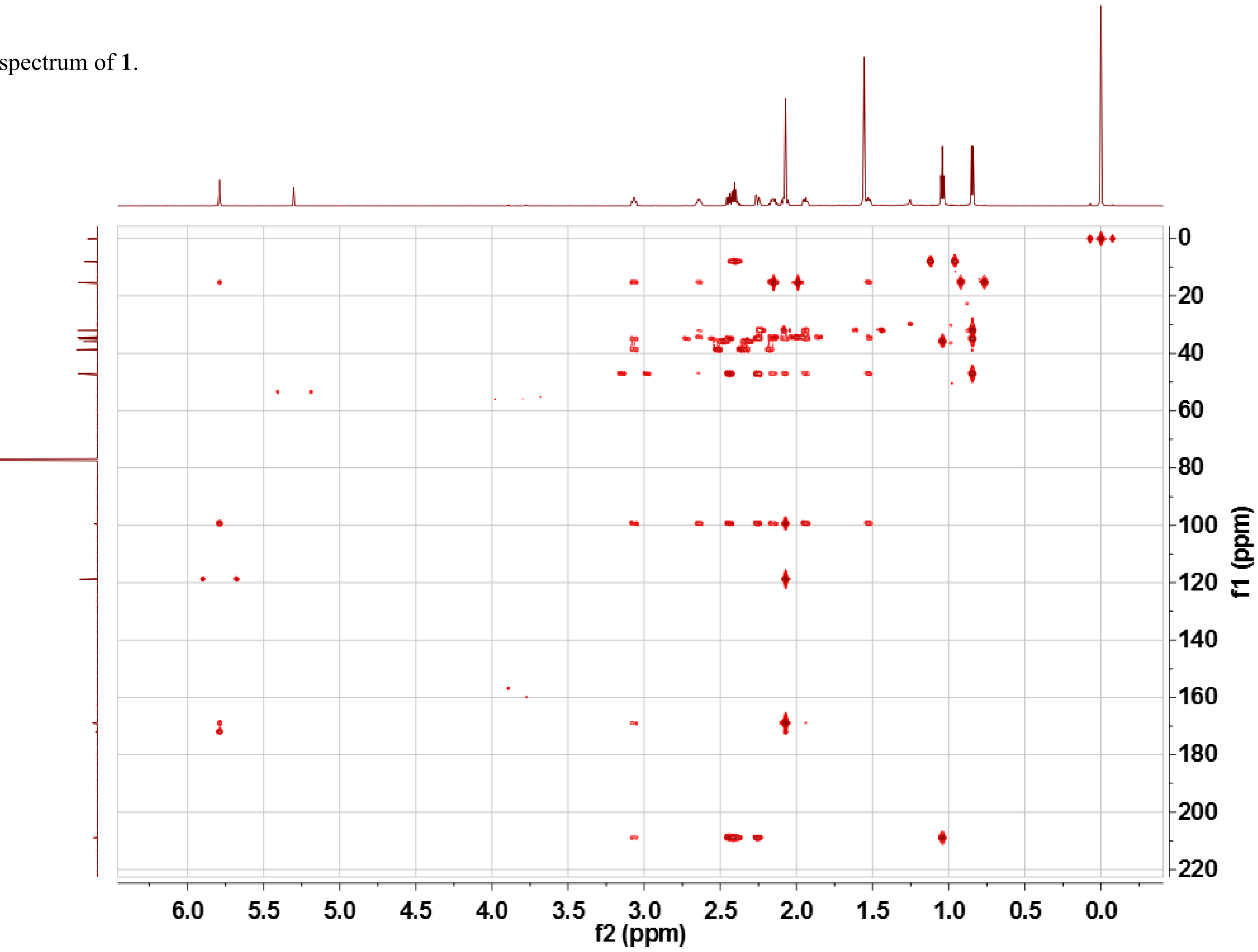

Figure S6. ROESY spectrum of **1**.

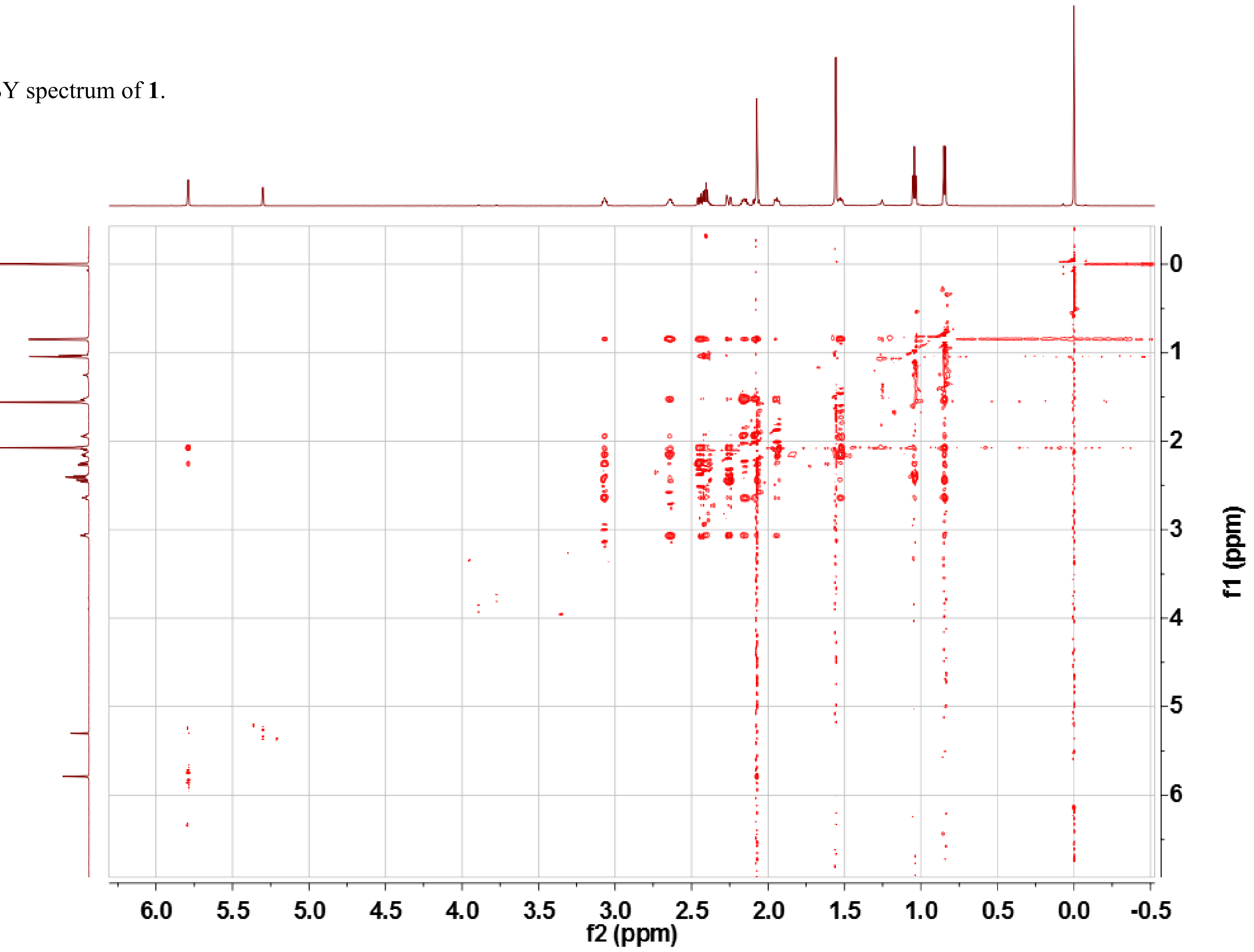

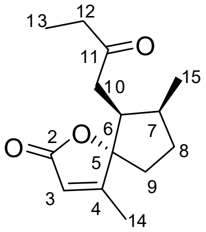

**Data Filename**	190521ESIA1.d	**Sample Name**	pec22
**Sample Type**	Sample	**Position**	
**Instrument Name**	Agilent G6230 TOF MS	**User Name**	KIB
**Acq Method**	ESI.m	**Acquired Time**	5/21/2019 12:21:35 PM
**IRM Calibration Status**	Success	**DA Method**	ESI.m
**Comment**			

**Sample Group**		**Info.**
**Acquisition SW Version**	6200 series TOF/6500 series Q-TOF B.05.01 (B5125.2)	

User Spectra

Fragmentor Voltage	Collision Energy	Ionization Mode
200	0	ESI

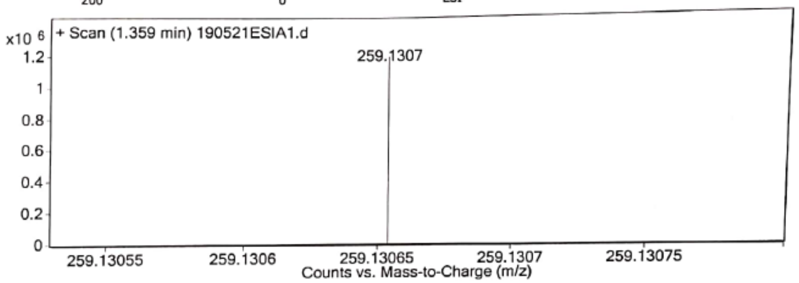

Peak List

m/z	z	Abund	Formula	Ion
102.1282	1	377527.59		
105.0253	1	61774.23		
121.0509	1	222906.16		
219.1373	1	71663.97		
259.1307	1	1198090.5	C14 H20 Na O3	M+
260.1337	1	167781.55	C14 H20 Na O3	M+
275.1042	1	138064.86		
300.1569	1	210484.33		
495.2723	1	191896.08		
496.275	1	56460.4		

Formula Calculator Element Limits

Element	Min	Max
C	0	200
H	0	400
O	0	10
Na	1	1

Formula Calculator Results

Formula	CalculatedMass	Mz	Diff.(mDa)	Diff. (ppm)	DBE
C14 H20 Na O3	259.1310	259.1307	0.3	1.2	4.5

--- End Of Report ---

Figure S7. HRESIMS spectrum of **1**.

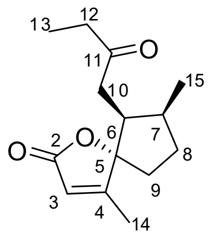

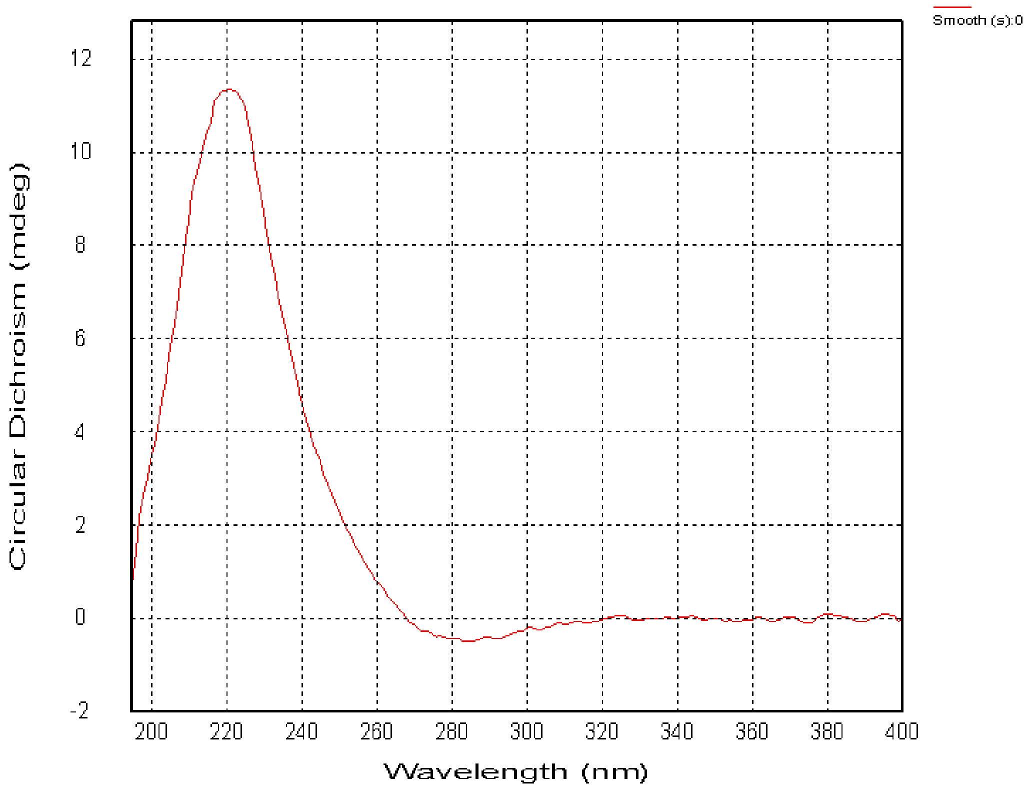

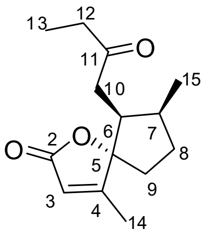

Figure S8. ECD spectrum of **1**.

File: CD **1**-1 mm(195-400).dsx
ProBinary X
Attributes:
- Time Stamp:Sun Jun 02 13:15:12 2019

- File ID: {F35A1C03-F468-406c-A311-534B3BCAE4CE}
- Is CFR Compliant: false
- Original data has not been modified.

Remarks:
- User: APLService
- Date: 2019/06/02
- Instrument: 0218
- DetectorType: PMT
- DichOS Calibration Correction Curve: 0218/1
- HV (CDDC channel): 0 v
- Time per point: 1 s
- Description: **1**
- Concentration: 0.1600mg/mL MeOH
- Pathlength: 1 mm
- Temperature: 20 °C

Settings:
- Time-per-point: 1s (25us x 40000)
- SE
- Wavelength: 195nm - 400nm
- Step Size: 1nm
- Bandwidth: 1nm

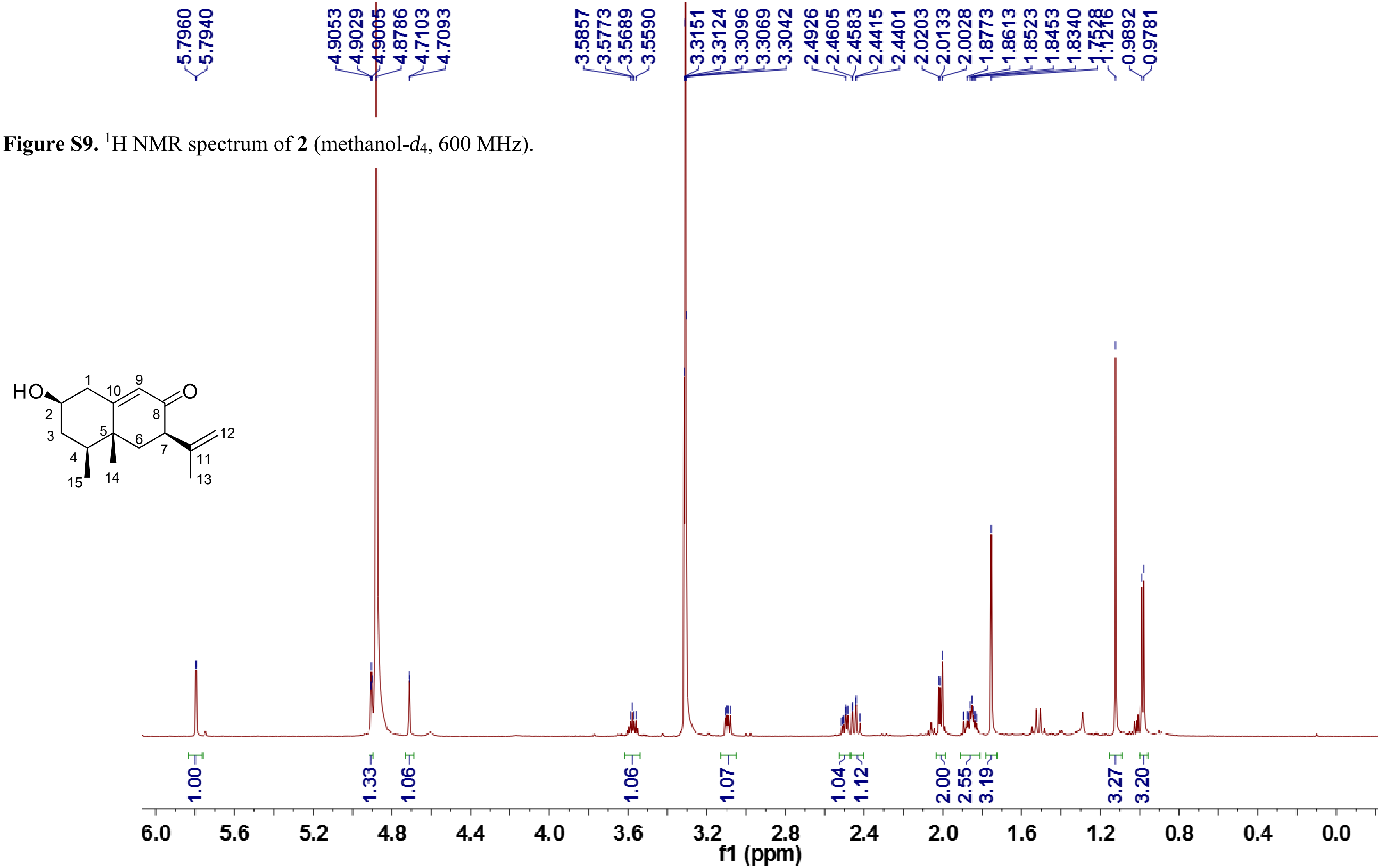

Figure S9. ^{1}H NMR spectrum of **2** (methanol-d_4, 600 MHz).

Figure S10. ^{13}C NMR spectrum of **2** (methanol-d_4, 151 MHz).

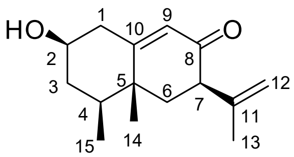

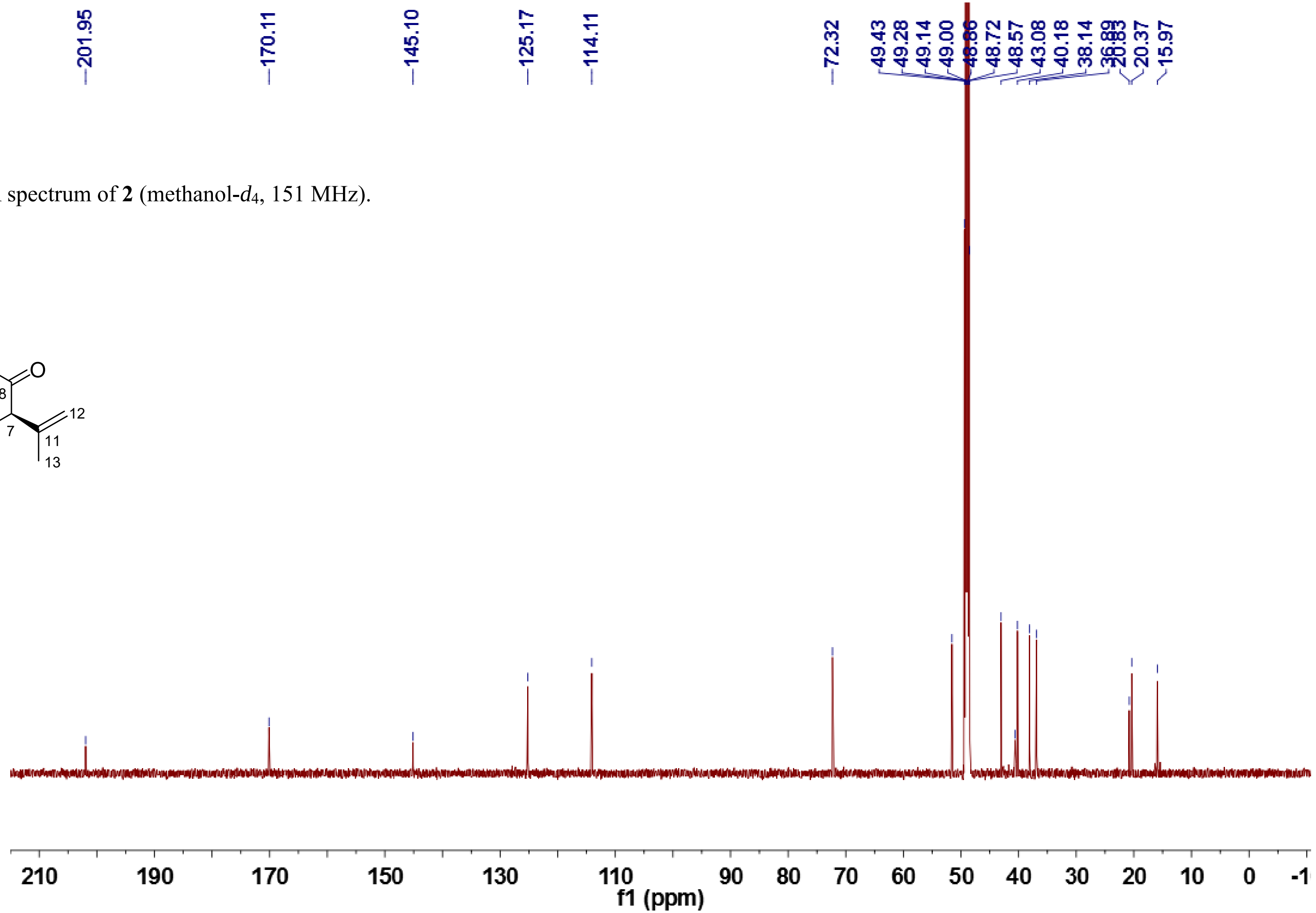

Figure S11. HSQC spectrum of **2**.

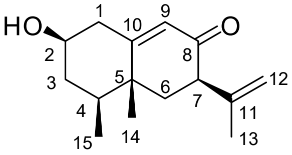

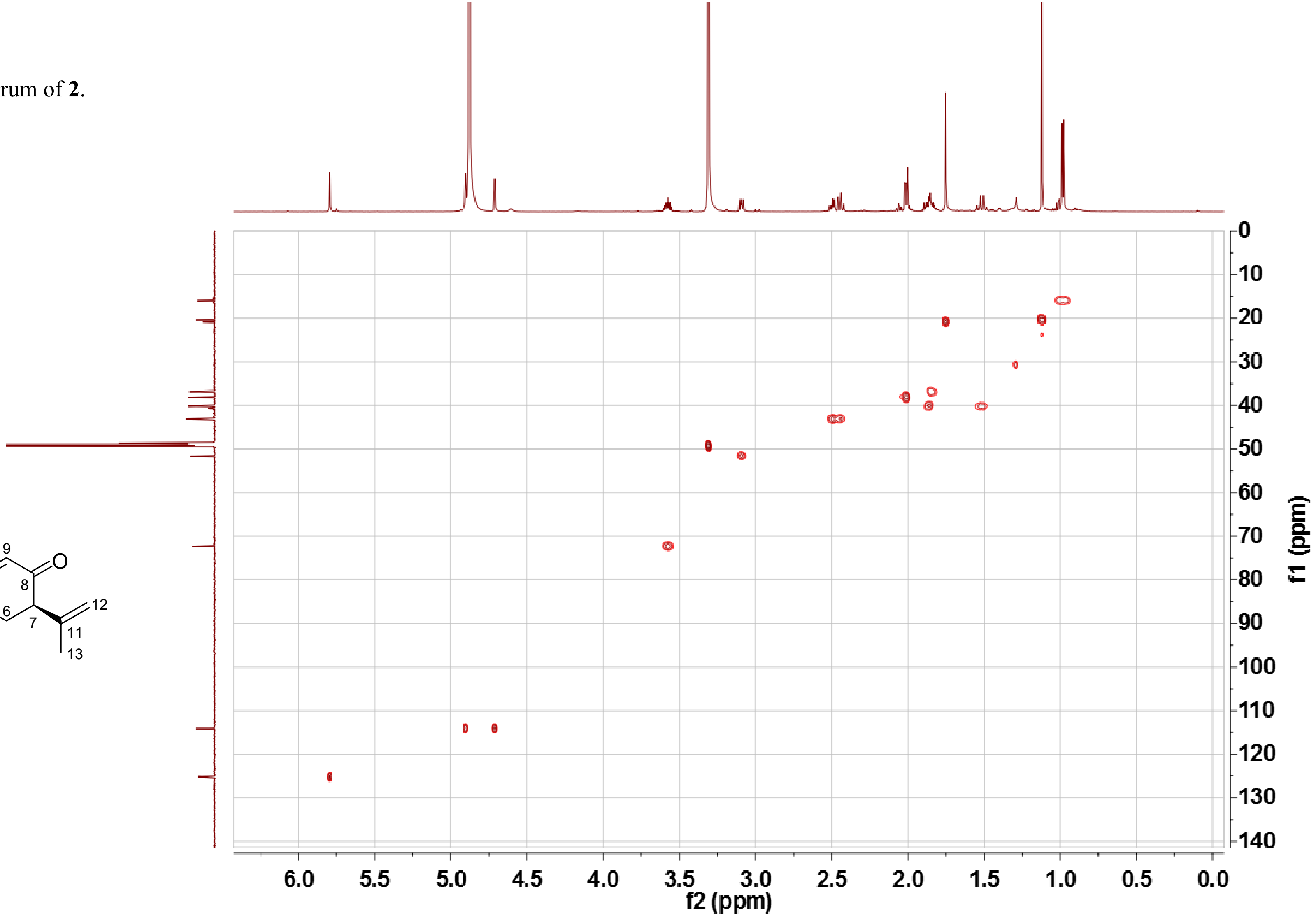

Figure S12. ^1H–^1H COSY spectrum of **2**.

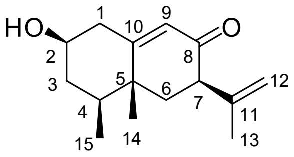

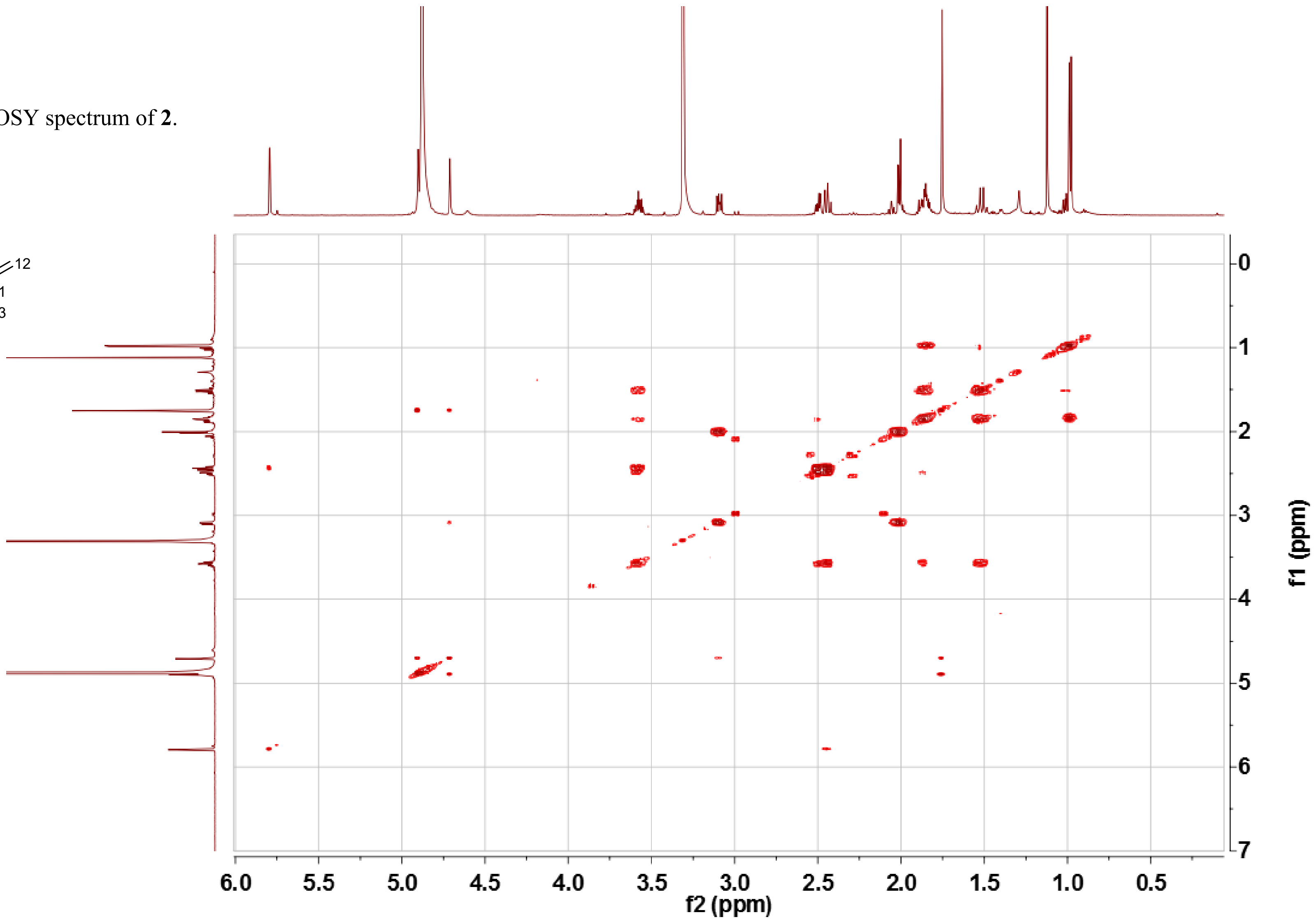

Figure S13. HMBC spectrum of **2**.

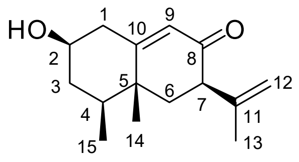

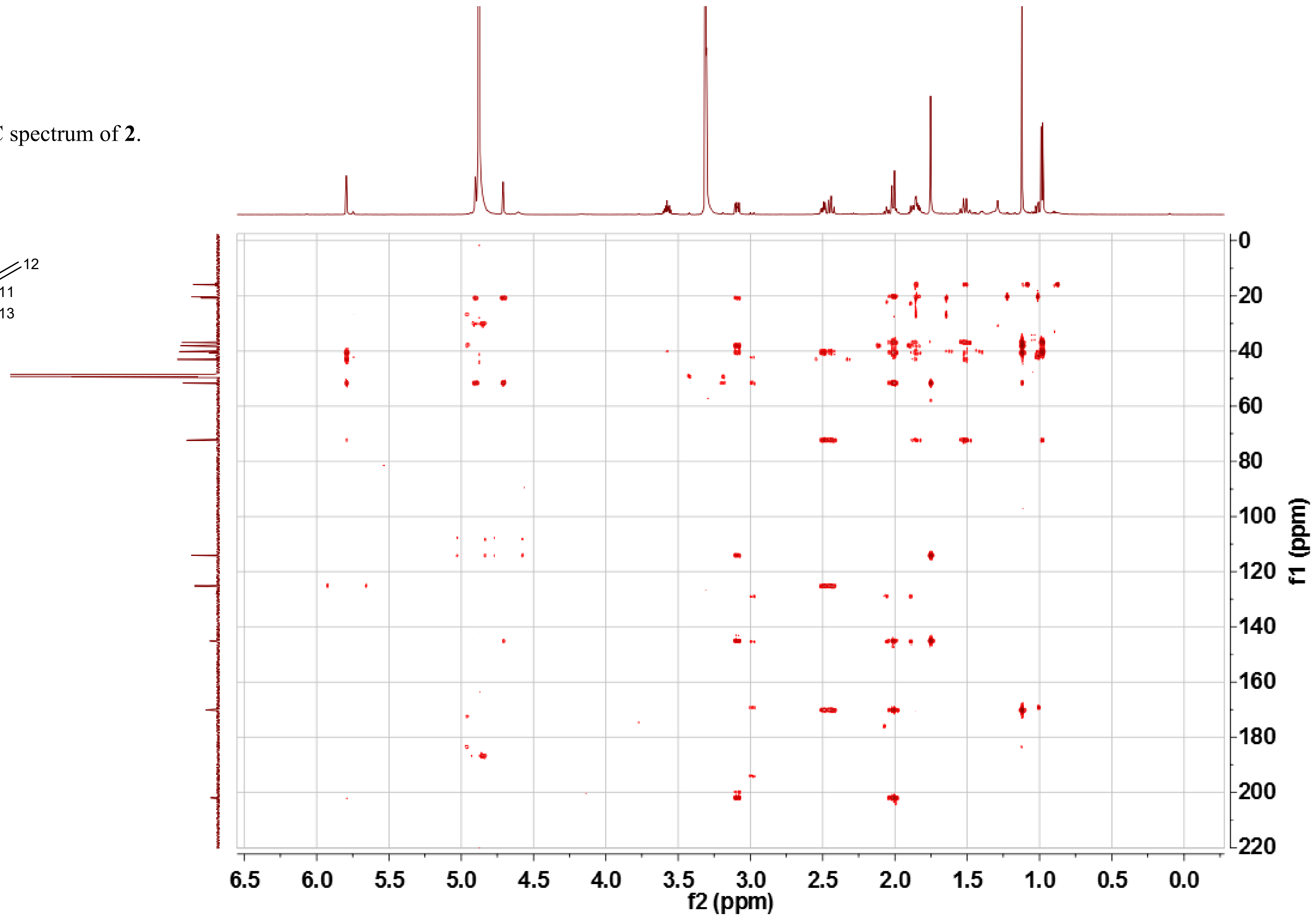

Figure S14. ROESY spectrum of **2**.

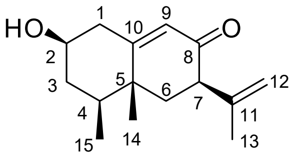

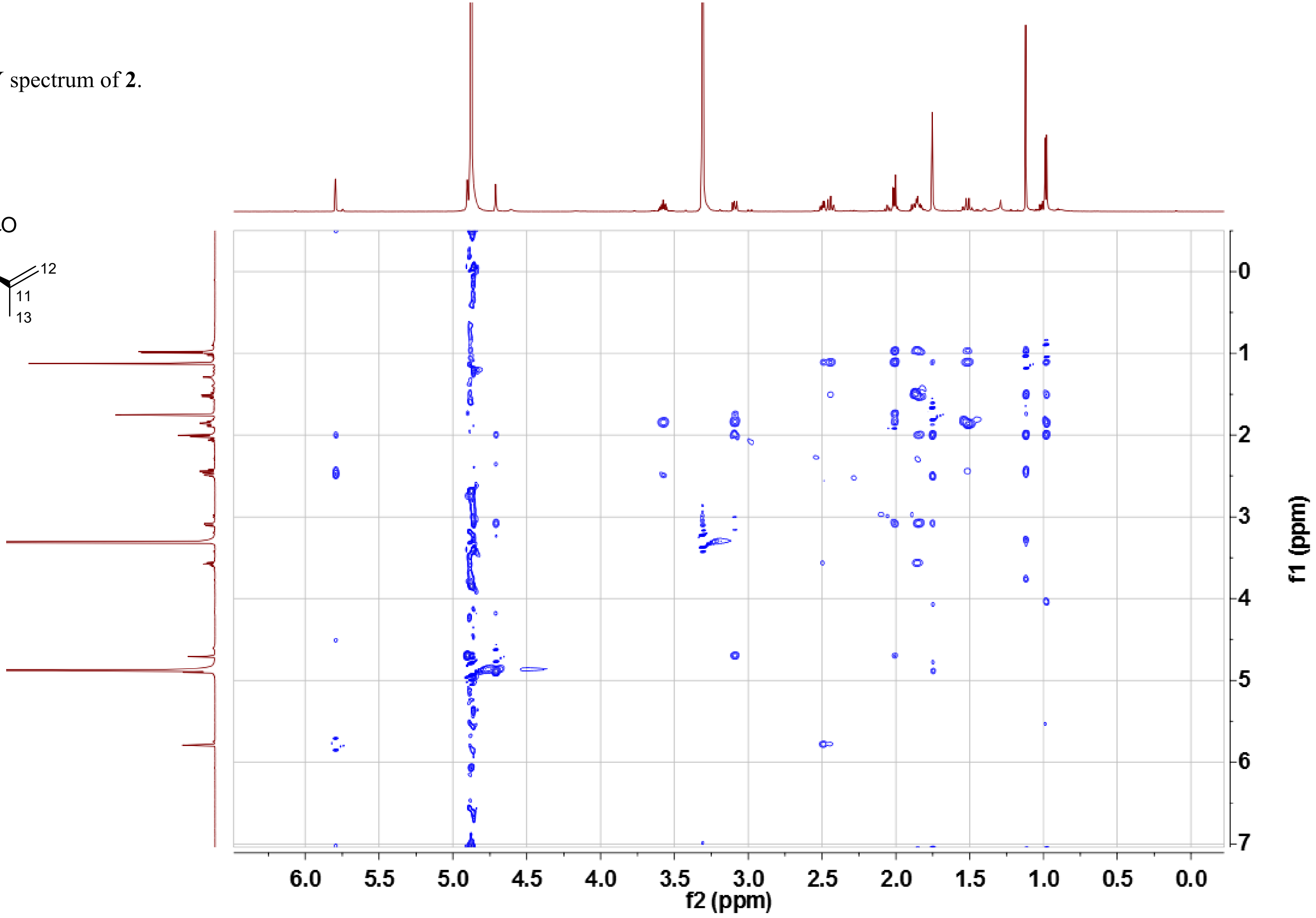

**Data Filename**	191108ESIA5.d	**Sample Name**	pec31
**Sample Type**	Sample	**Position**	
**Instrument Name**	Agilent G6230 TOF MS	**User Name**	KIB
**Acq Method**	ESI.m	**Acquired Time**	11/6/2019 11:02:00 AM
**IRM Calibration Status**	Success	**DA Method**	ESI.m
**Comment**			

Sample Group **Info.**

Acquisition SW Version 6200 series TOF/6500 series Q-TOF B.05.01 (B5125.2)

User Spectra

Fragmentor Voltage	Collision Energy	Ionization Mode
200	0	ESI

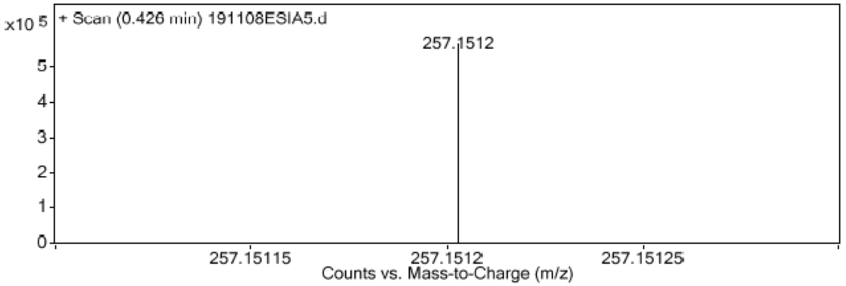

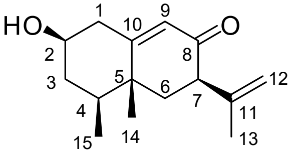

Peak List

m/z	z	Abund	Formula	Ion
121.0509	1	294561.38		
257.1512	1	564974.38	C15 H22 Na O2	M+
271.1304	1	197453.64		
273.1457	1	1222431.5		
275.161	1	275586.31		
301.1411	1	294627.06		
467.2137	1	475393.81		
489.1956	1	495956.91		
490.1969	1	193567		
491.3132	1	446649.56		

Formula Calculator Element Limits

Element	Min	Max
C	0	200
H	0	400
O	0	10
Na	1	1

Formula Calculator Results

Formula	CalculatedMass	Mz	Diff.(mDa)	Diff. (ppm)	DBE
C15 H22 Na O2	257.1518	257.1512	0.5	2.1	4.5

--- End Of Report ---

Figure S15. HRESIMS spectrum of **2**.

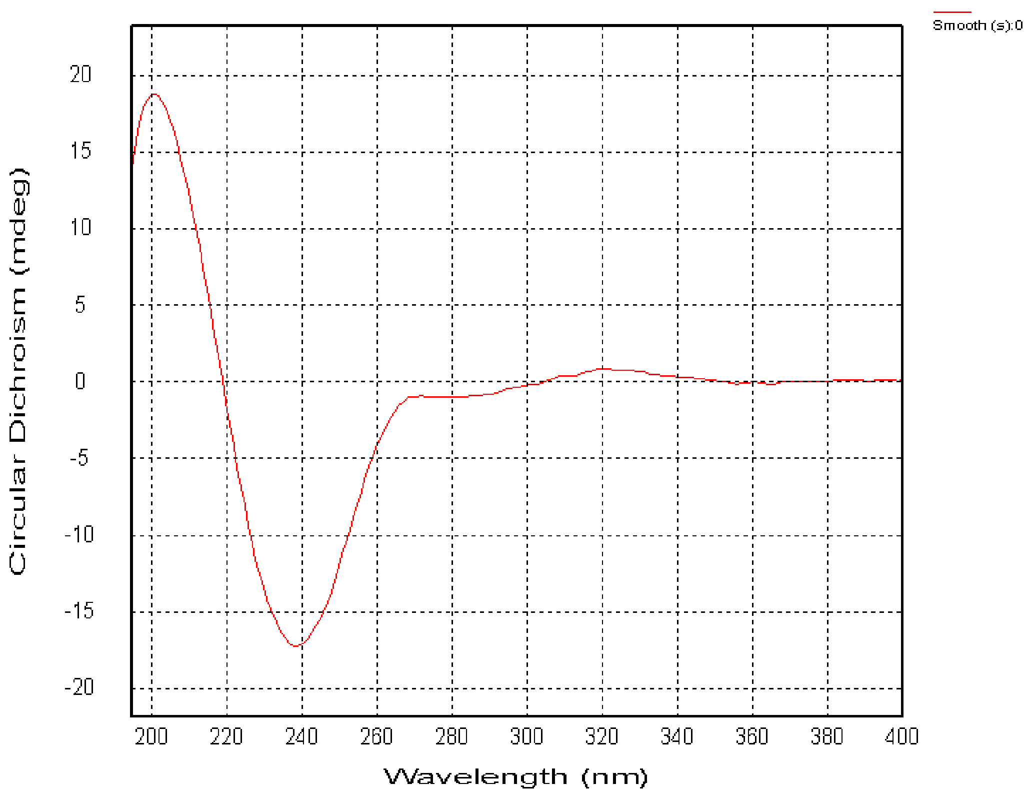

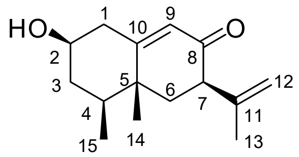

Figure S16. ECD spectrum of **2**.

File: CD **2**-1 mm(195-400).dsx
ProBinaryX
Attributes:
- Time Stamp:Sat Jul 20 16:26:39 2019

- File ID: {E7AE9F70-2EF2-4cb4-9A3A-ADA25586F6E0}
- Is CFR Compliant: false
- Original data has not been modified.

Remarks:
- User: APL Service
- Date: 2019/07/20
- Instrument: 0218
- Detector Type: PMT
- DichOS Calibration Correction Curve: 0218/1
- HV (CDDC channel): 0 v
- Time per point: 1 s
- Description: **2**
- Concentration: 0.1800mg/mL MeOH
- Pathlength: 1 mm
- Temperature: 20 °C

Settings:
- Time-per-point: 1s (25us x 40000)
- SE
- Wavelength: 195nm - 400nm
- Step Size: 1nm
- Bandwidth: 1nm

Figure S17. ^{1}H NMR spectrum of **3** (methanol-d_4, 800 MHz).

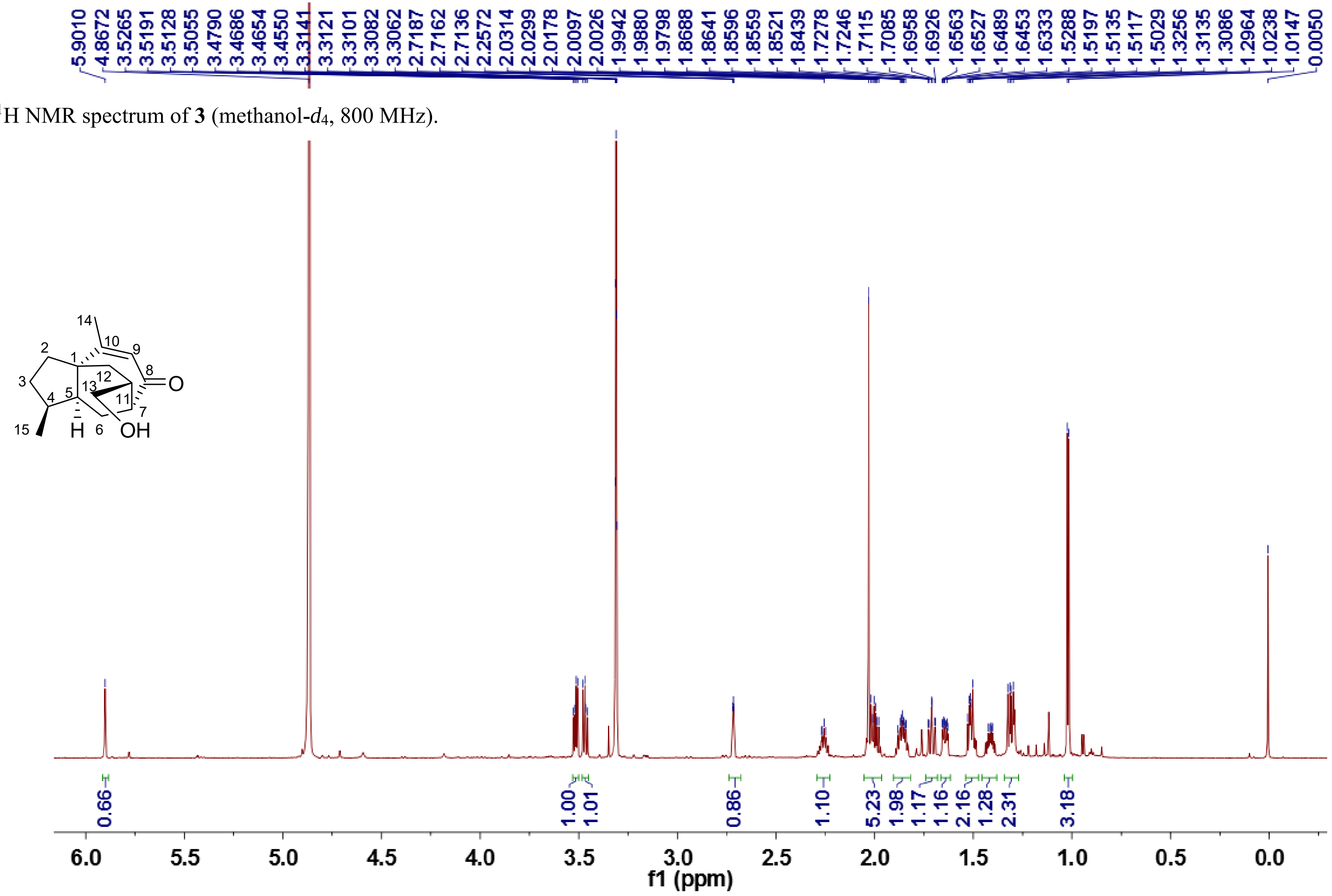

Figure S18. ^{13}C NMR spectrum of **3** (methanol-d_4, 201 MHz).

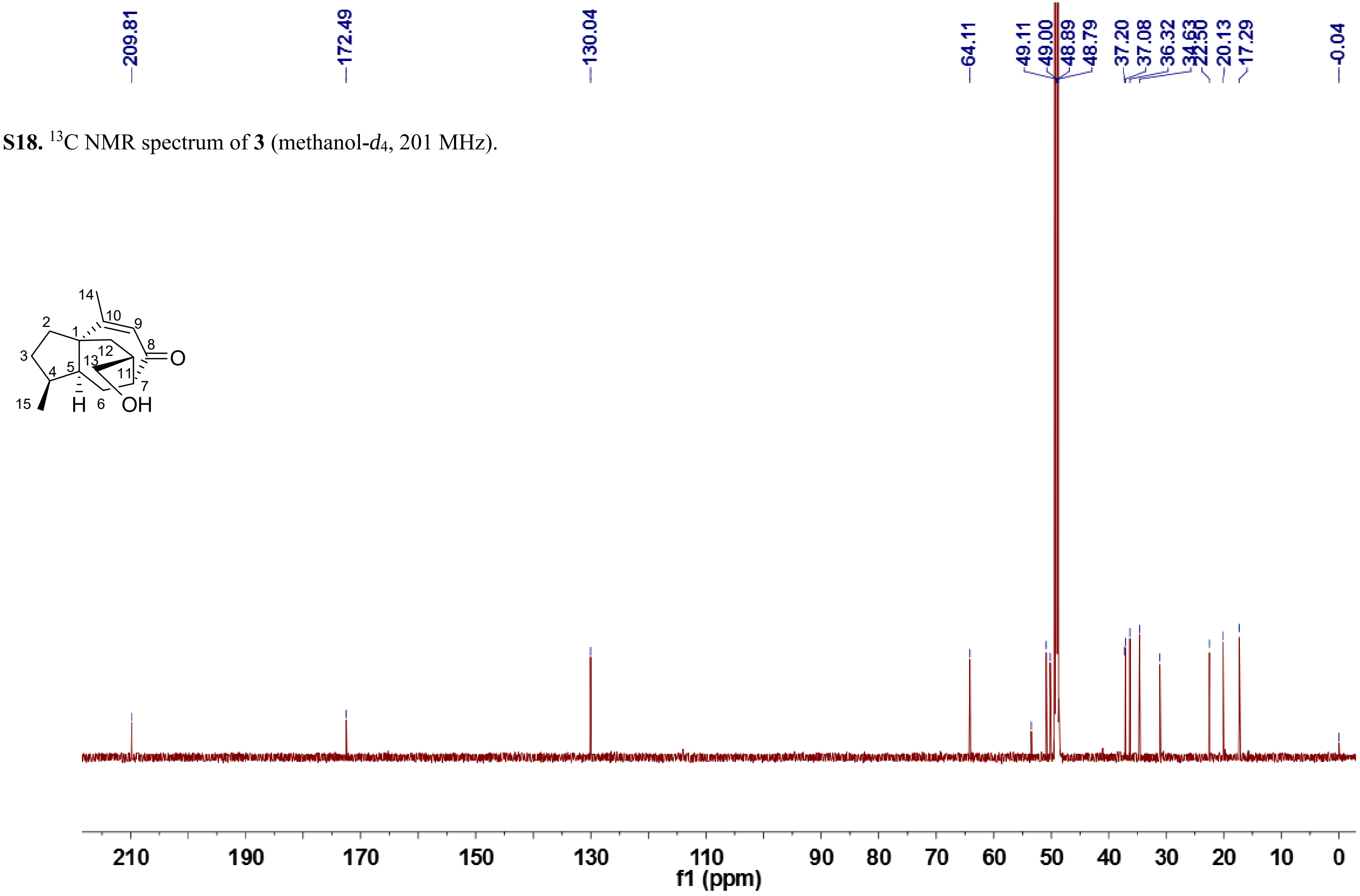

Figure S19. HSQC spectrum of **3**.

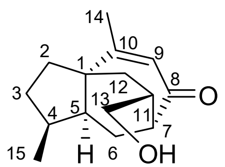

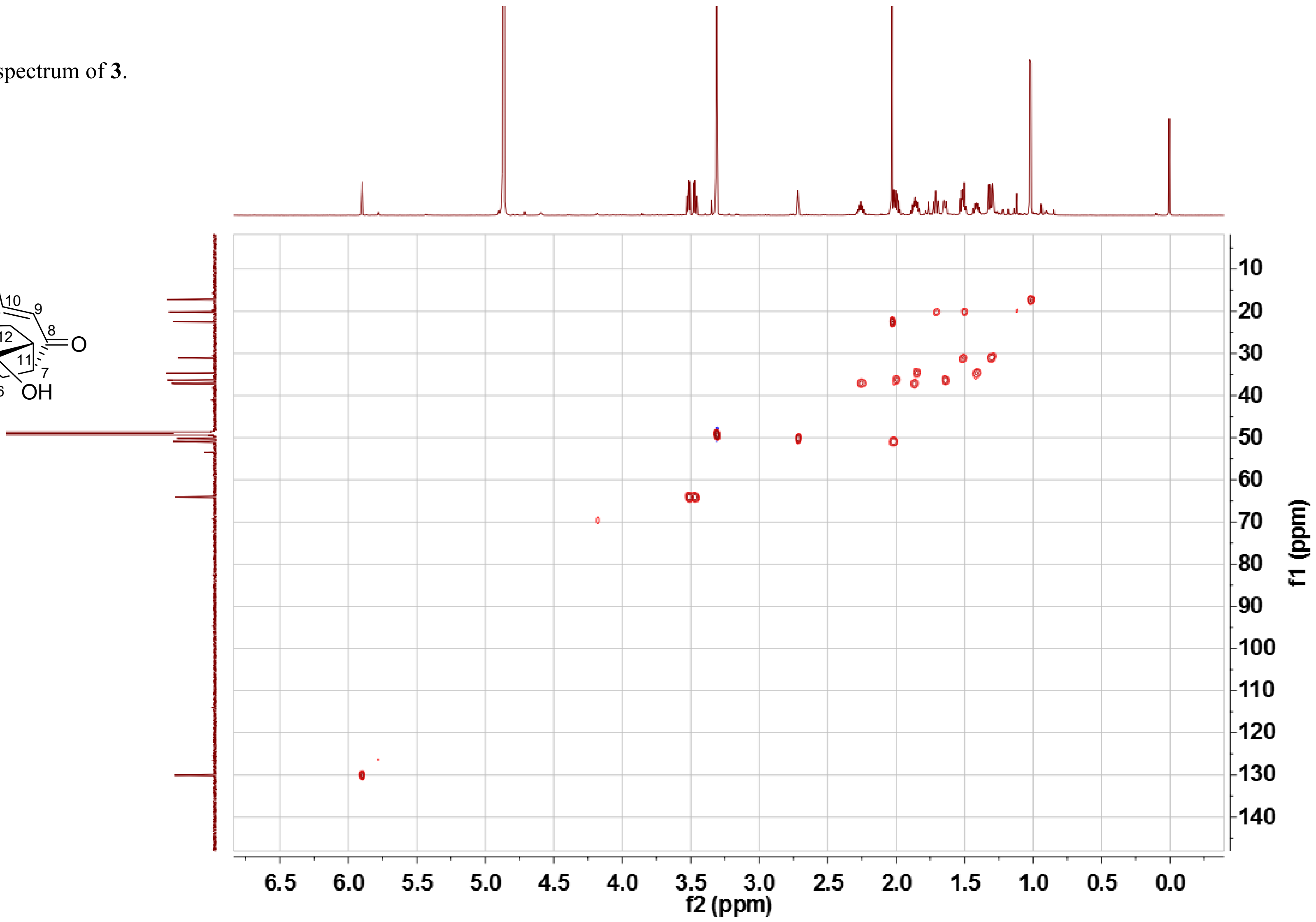

Figure S20. ^{1}H–^{1}H COSY spectrum of **3**.

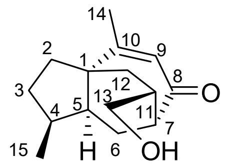

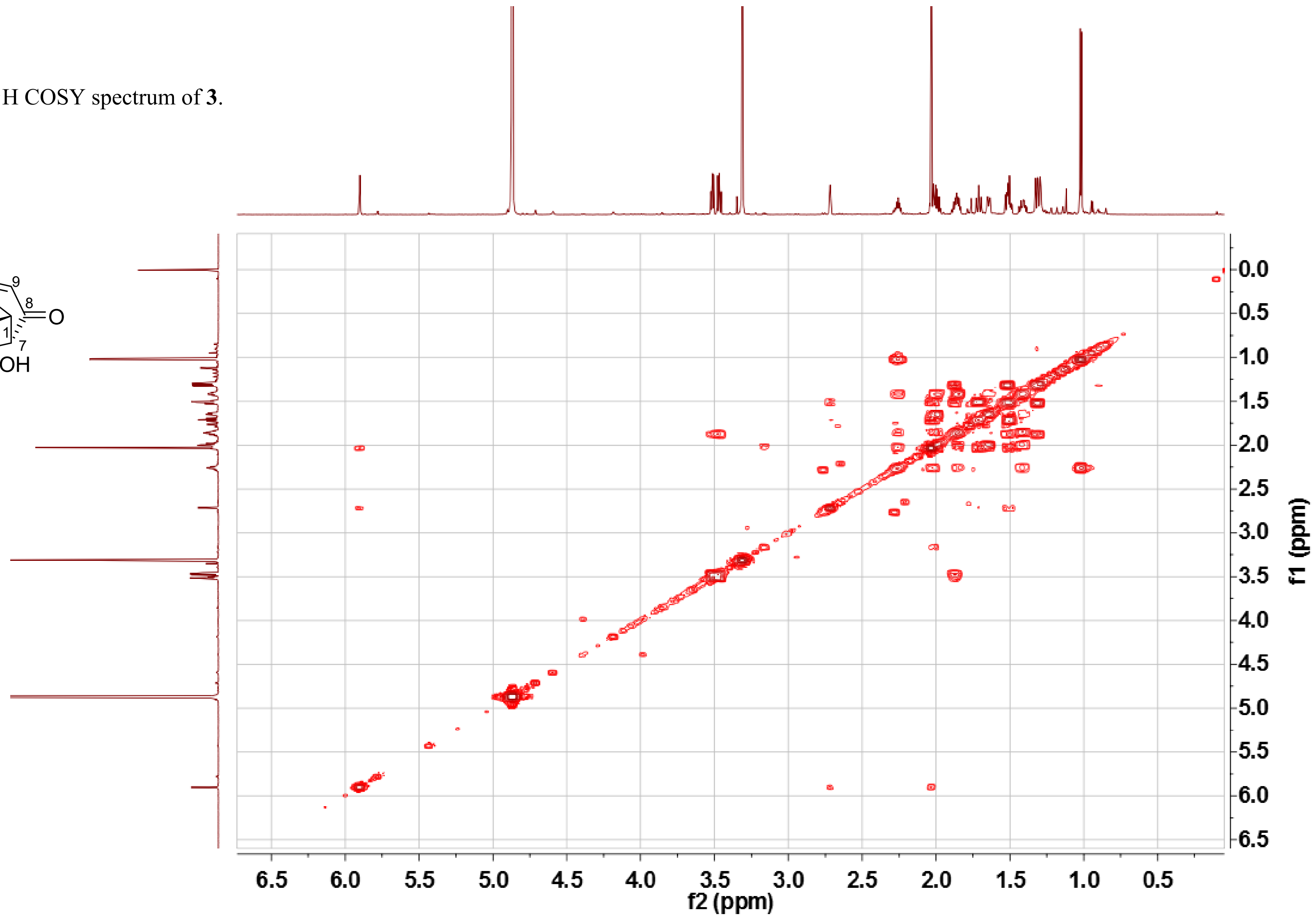

Figure S21. HMBC spectrum of **3**.

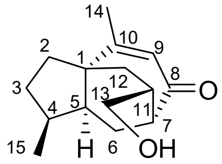

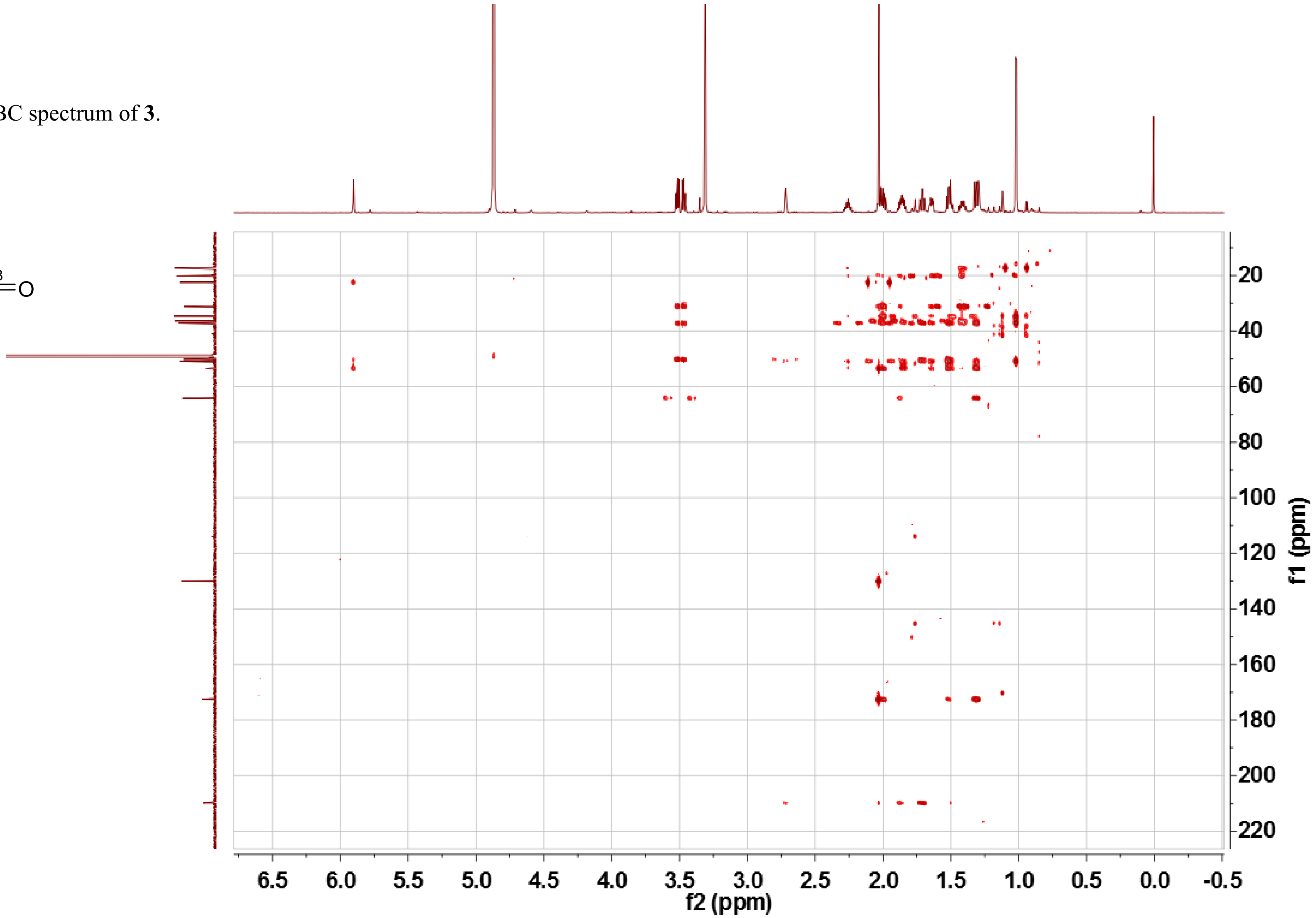

Figure S22. ROESY spectrum of **3**.

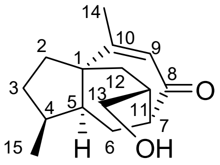

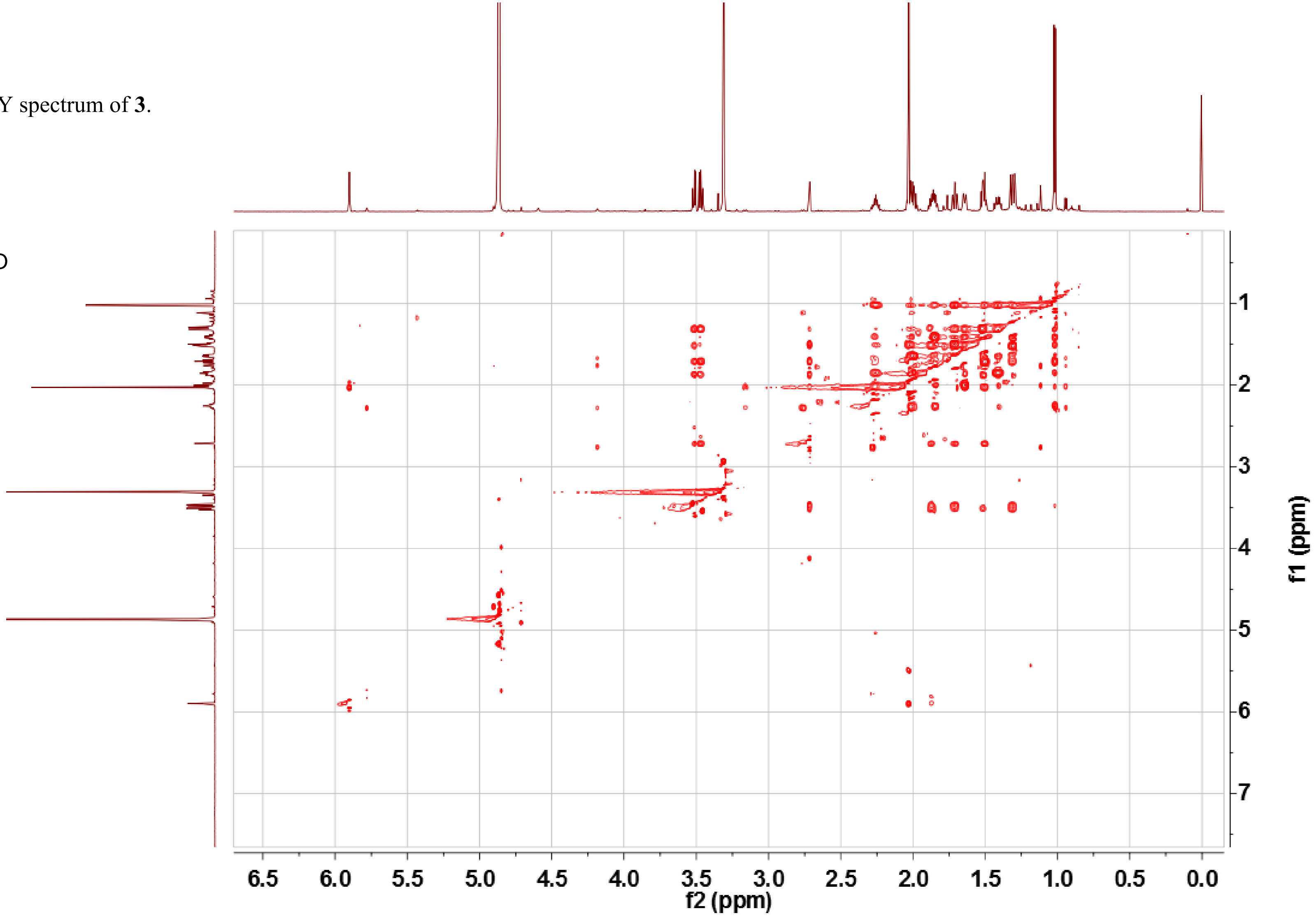

**Data Filename**	190620ESIA1.d	**Sample Name**	pec32
**Sample Type**	Sample	**Position**	
**Instrument Name**	Agilent G6230 TOF MS	**User Name**	KIB
**Acq Method**	ESI.m	**Acquired Time**	6/20/2019 10:40:47 AM
**IRM Calibration Status**	Success	**DA Method**	ESI.m
**Comment**			

**Sample Group**		**Info.**
**Acquisition SW Version**	6200 series TOF/6500 series Q-TOF B.05.01 (B5125.2)	

User Spectra

Fragmentor Voltage	Collision Energy	Ionization Mode
200	0	ESI

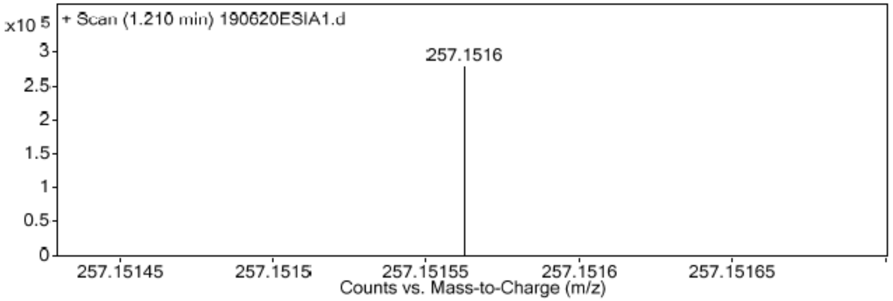

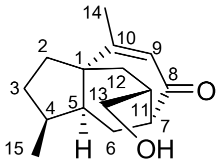

Peak List

m/z	z	Abund	Formula	Ion
102.1283	1	64055.63		
121.0509		48665.35		
257.1516	1	277805.94	C15 H22 Na O2	M+
273.144	1	145908.52		
298.1785	1	415117.88		
299.181	1	76627.3		
314.1731	1	122140.73		
491.3147	1	404511.69		
492.3177	1	127388.12		
507.3083	1	90403.14		

Formula Calculator Element Limits

Element	Min	Max
C	0	200
H	0	400
O	0	10
Na	1	1

Formula Calculator Results

Formula	CalculatedMass	Mz	Diff.(mDa)	Diff. (ppm)	DBE
C15 H22 Na O2	257.1518	257.1516	0.2	0.6	4.5

--- End Of Report ---

Figure S23. HRESIMS spectrum of **3**.

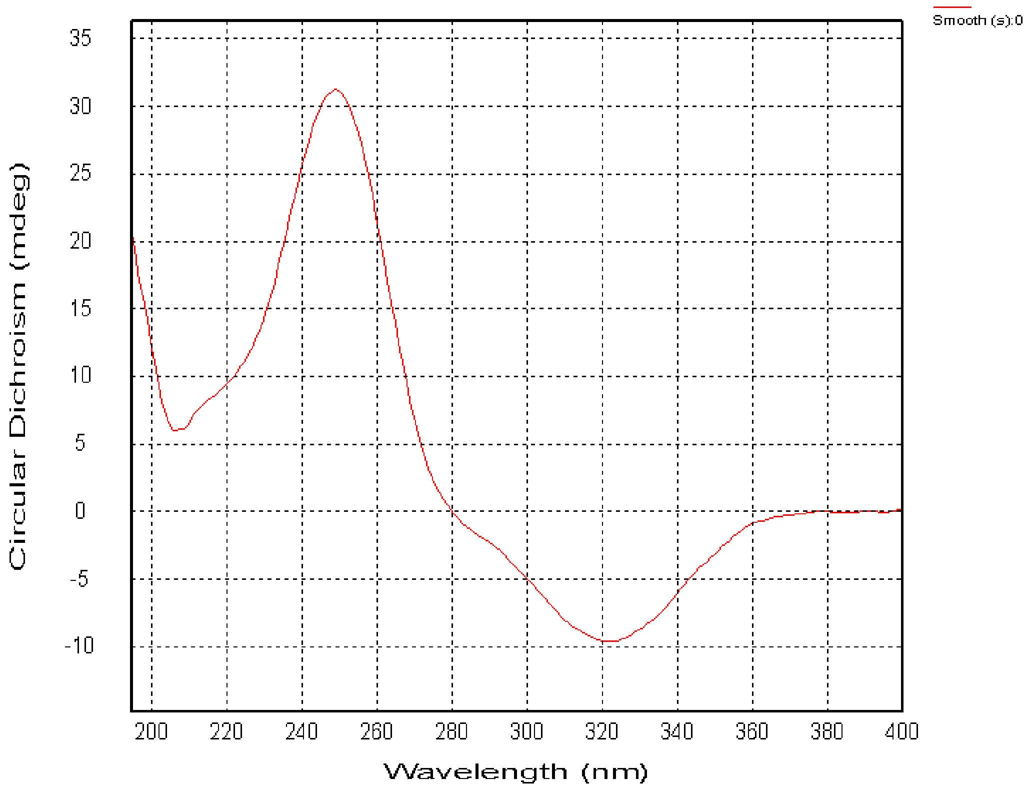

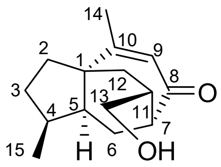

Figure S24. ECD spectrum of **3**.

File: CD **3**-1 mm(195-400).dsx
ProBinary X
Attributes:
- Time Stamp: Wed Jun 26 18:42:02 2019

- File ID: {3B254B2E-5860-4dab-B8A6-C575AD203F79}
- Is CFR Compliant: false
- Original data has not been modified.

Remarks:
- User: APL Service
- Date: 2019/06/26
- Instrument: 0218
- Detector Type: PMT
- DichOS Calibration Correction Curve: 0218/1
- HV (CDDC channel): 0 v
- Time per point: 1 s
- Description: **3**
- Concentration: 0.1820mg/mL MeOH
- Pathlength: 1 mm
- Temperature: 20 °C

Settings:
- Time-per-point: 1s (25us x 40000)
- SE
- Wavelength: 195nm - 400nm
- Step Size: 1nm
- Bandwidth: 1nm

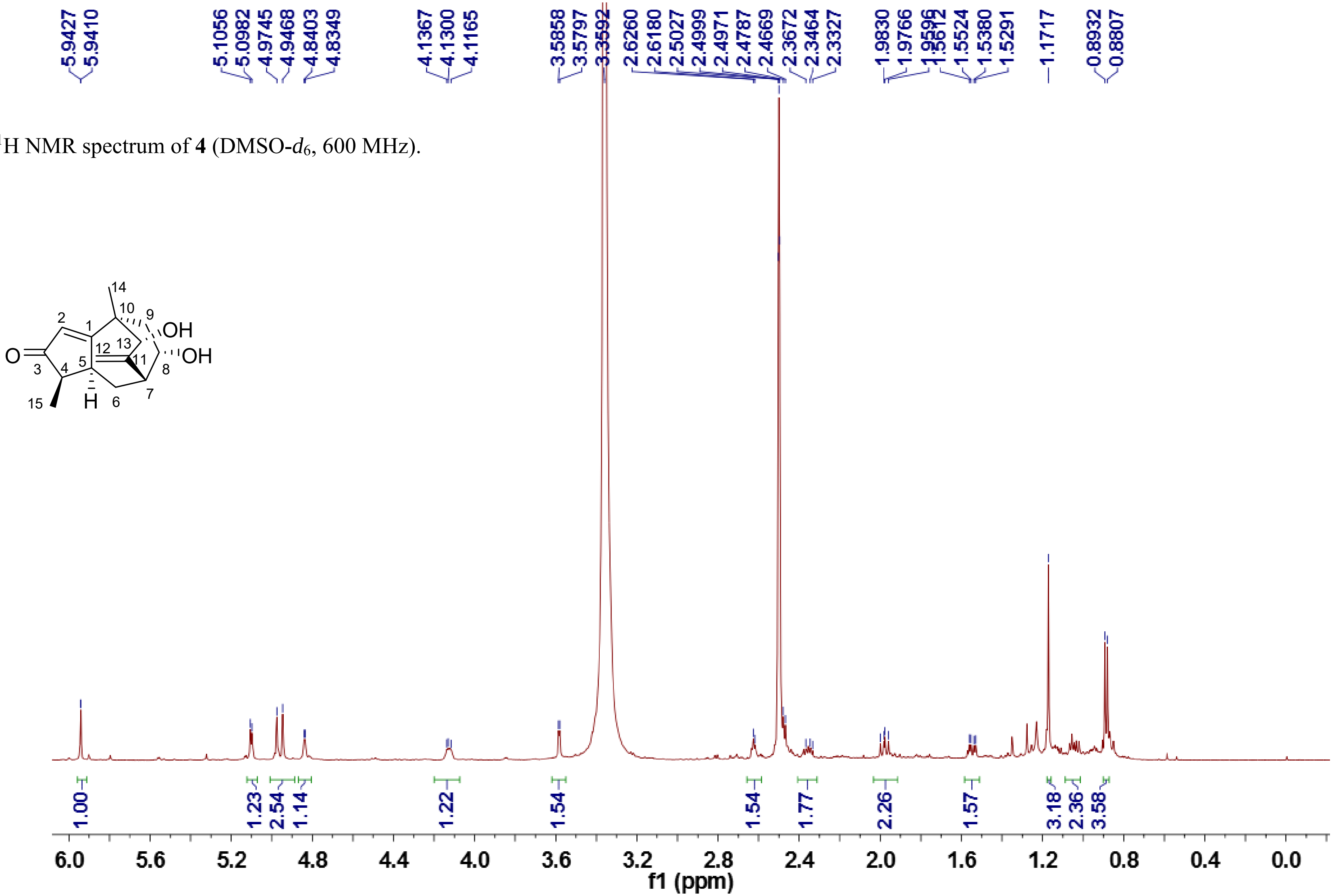

Figure S25. ^{1}H NMR spectrum of **4** (DMSO-d_6, 600 MHz).

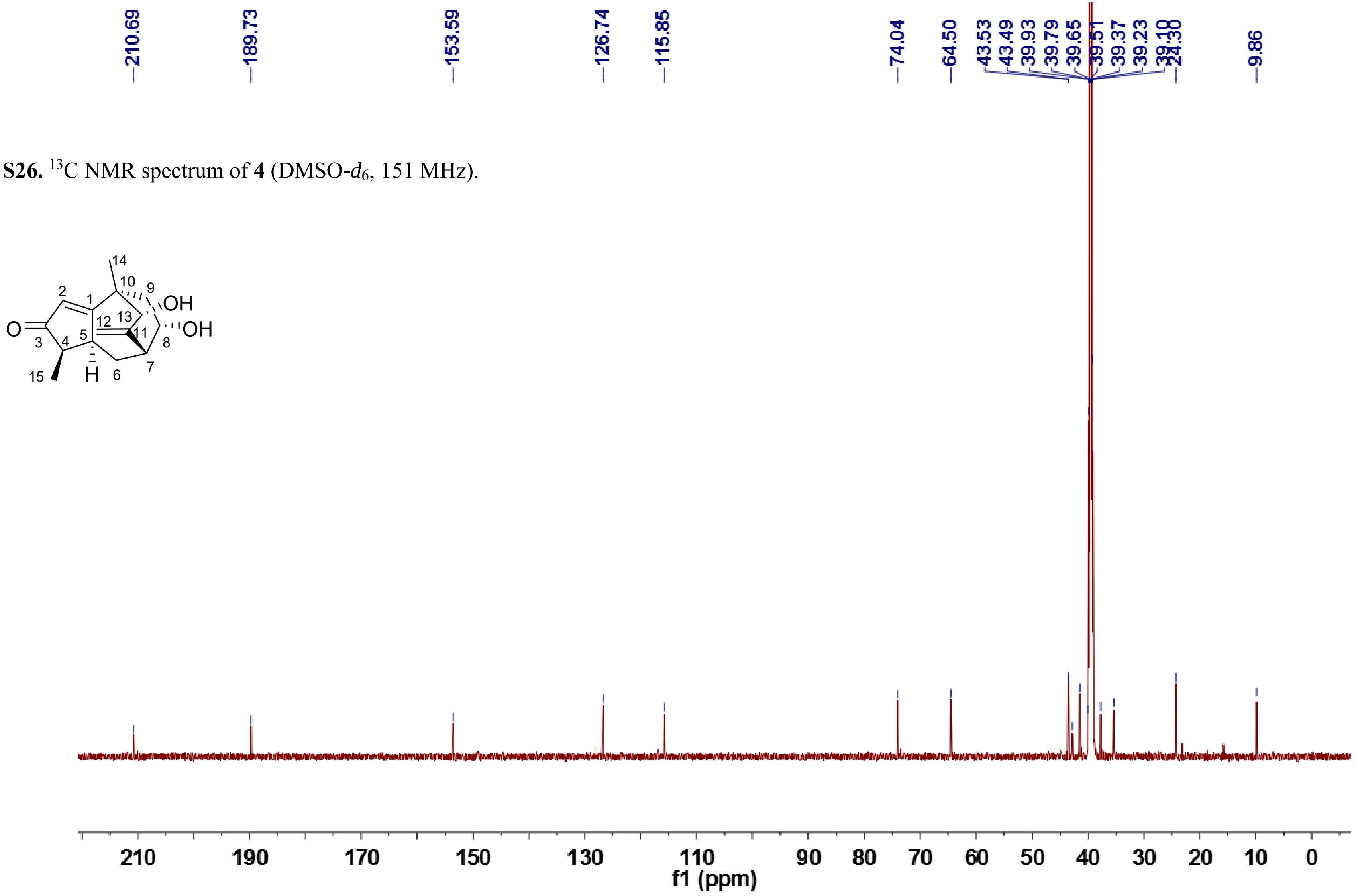

Figure S26. ^{13}C NMR spectrum of **4** (DMSO-d_6, 151 MHz).

Figure S27. HSQC spectrum of **4** in DMSO-d_6.

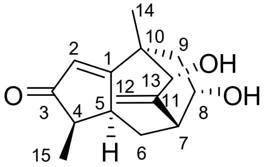

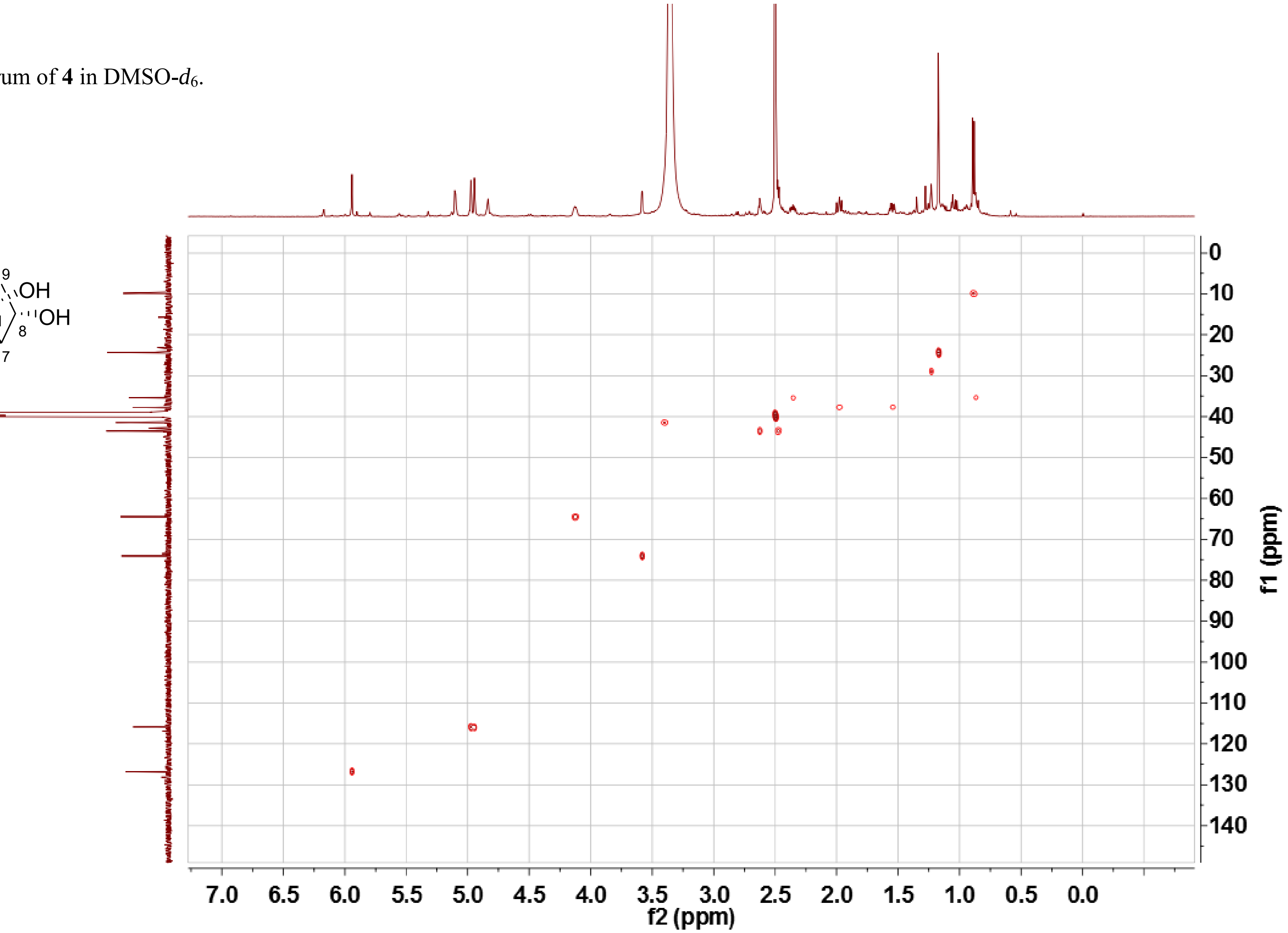

Figure S28. ^{1}H–^{1}H COSY spectrum of **4** in DMSO-d_6.

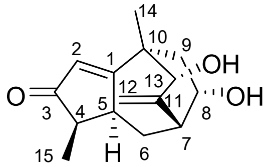

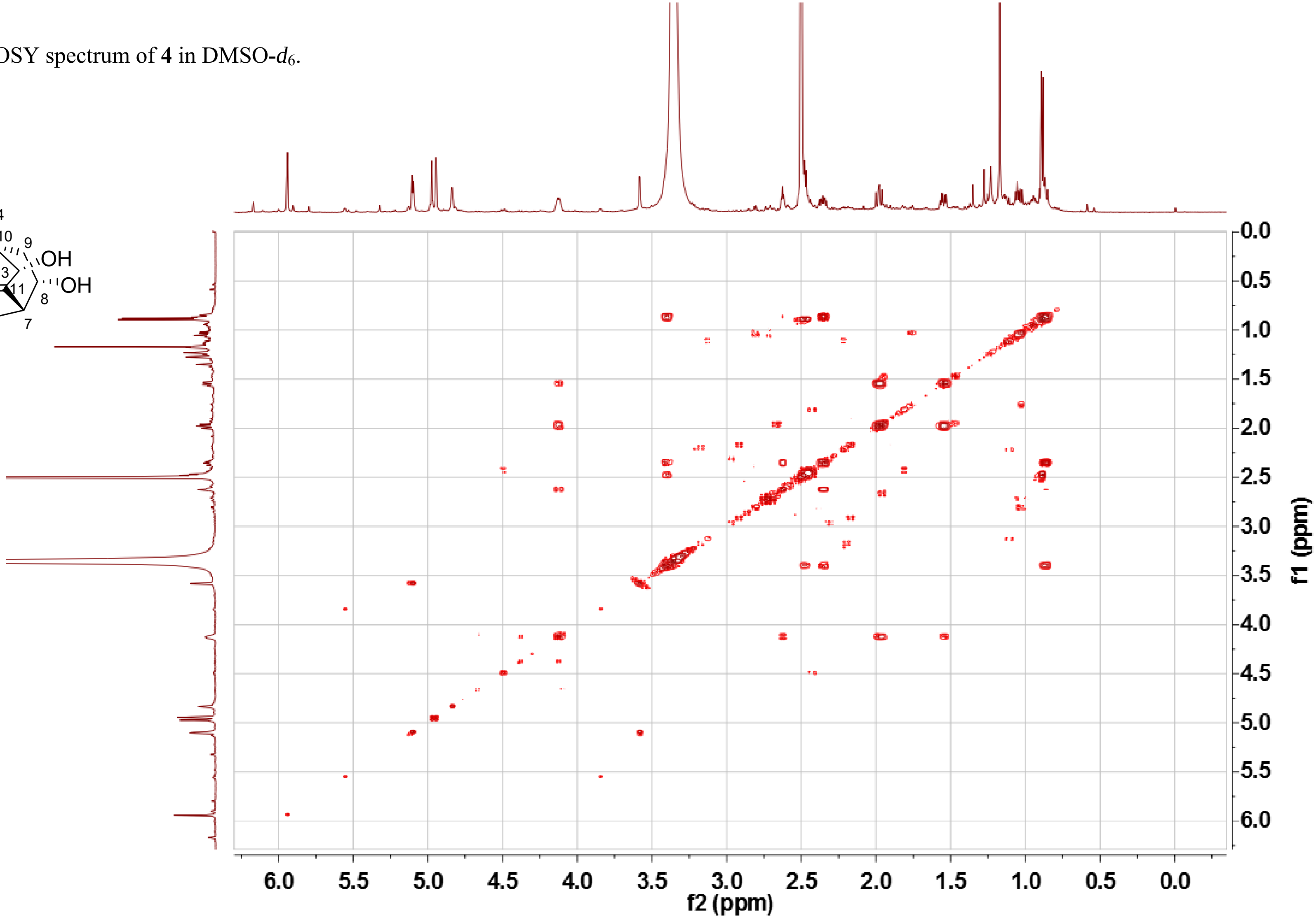

Figure S29. HMBC spectrum of **4** in DMSO-d_6.

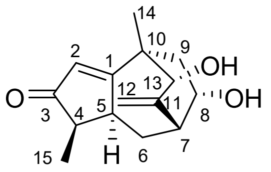

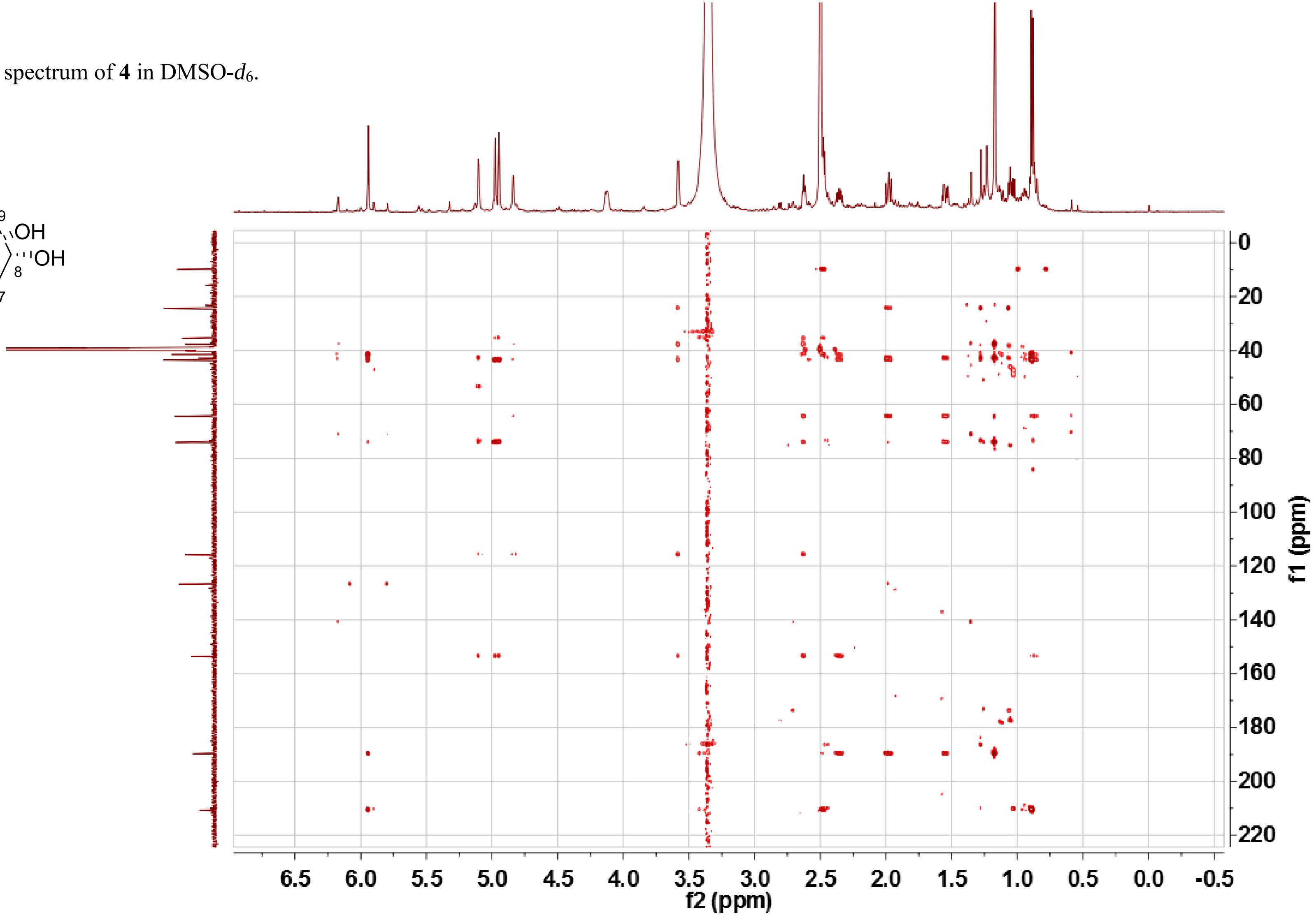

Figure S30. ROESY spectrum of **4** in DMSO-d_6.

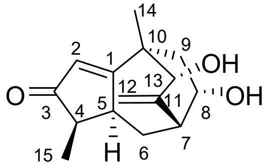

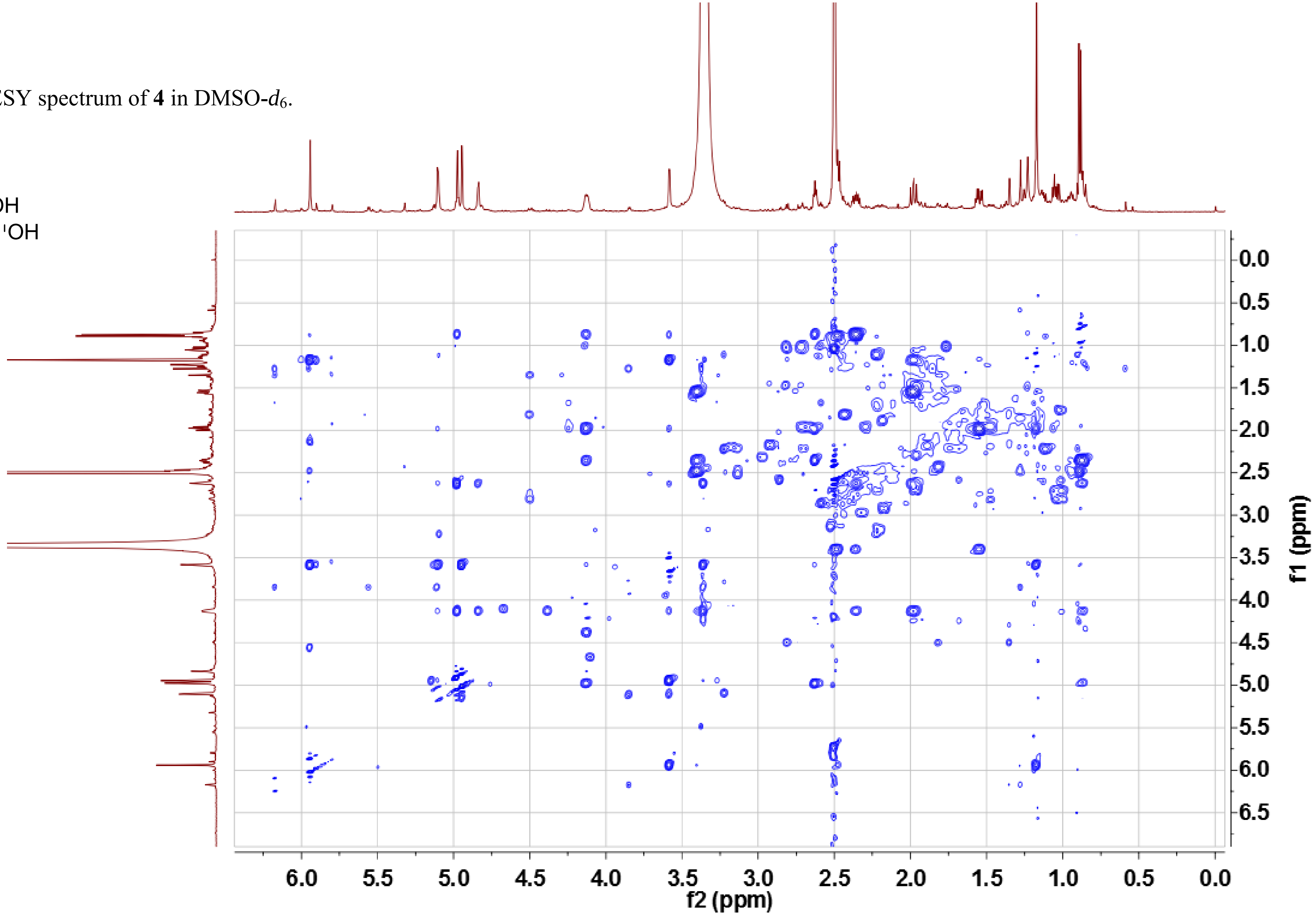

**Data Filename**	190613ESIA1.d	**Sample Name**	pec27
**Sample Type**	Sample	**Position**	
**Instrument Name**	Agilent G6230 TOF MS	**User Name**	KIB
**Acq Method**	ESI.m	**Acquired Time**	6/11/2019 10:21:48 AM
**IRM Calibration Status**	Success	**DA Method**	ESI.m
**Comment**			

**Sample Group**		**Info.**
**Acquisition SW Version**	6200 series TOF/6500 series Q-TOF B.05.01 (B5125.2)	

User Spectra

Fragmentor Voltage	Collision Energy	Ionization Mode
200	0	ESI

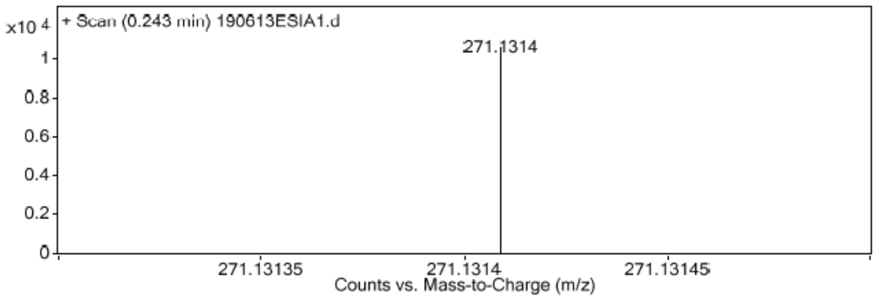

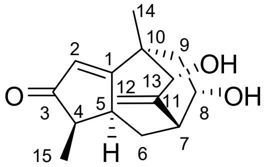

Peak List

m/z	z	Abund
249.1493	1	20031.28
275.1263	1	16395.46
291.1193	1	19286.94
303.121	1	18757.75
305.1377	1	29961.96
312.159	1	13609.95
319.1064	1	14597.78
321.1213	1	12646.91
344.148	1	11883.48
583.2536	1	11185.04

Formula Calculator Element Limits

Element	Min	Max
C	0	200
H	0	400
O	0	10
Na	1	1

Formula Calculator Results

Formula	CalculatedMass	Mz	Diff.(mDa)	Diff. (ppm)	DBE
C15 H20 Na O3	271.1310	271.1314	-0.4	1.4	5.5

--- End Of Report ---

Figure S31. HRESIMS spectrum of **4**.

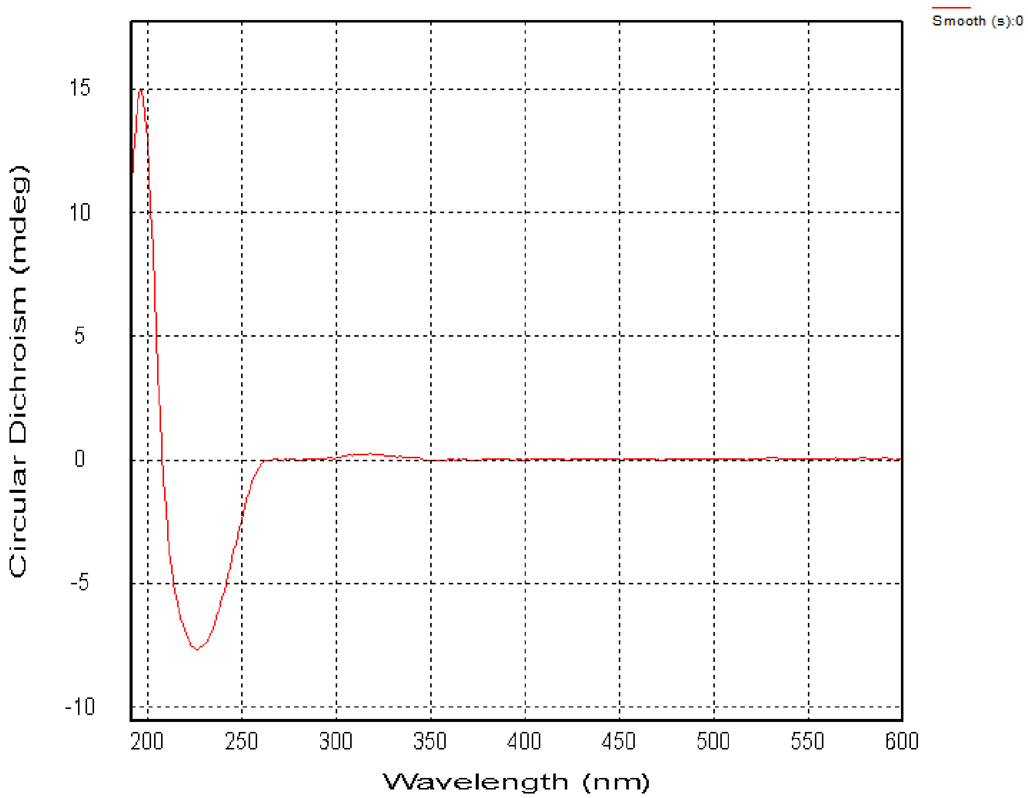

Figure S32. ECD spectrum of **4**.

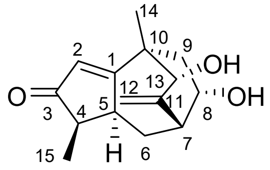

File: CD **4**-1 mm(195-600).dsx
ProBinary X
Attributes:
- Time Stamp: Wed Jun 05 15:02:34 2019

- File ID: {5E6D59A9-45F0-4e16-8239-A2A361109D72}
- Is CFR Compliant: false
- Original data has not been modified.

Remarks:
- User: CD
- Date: 2019/06/05
- Instrument: 0547
- Detector Type: LAAPD
- DichOS Calibration Correction Curve: 0547/2
- HV (CDDC channel): 0 v
- Time per point: 0.25 s
- Description: **4**
- Concentration: 0.0900 mg/ml MeOH
- Pathlength: 1 mm
- Temperature: 20 °C

Settings:
- Time-per-point: 0.25s (25us x 10000)
- SE
- Wavelength: 192nm - 600nm
- Step Size: 1nm
- Bandwidth: 1nm

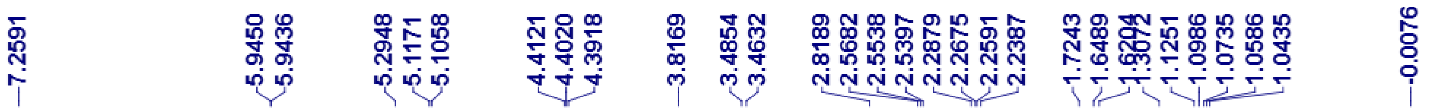

Figure S33. ^{1}H NMR spectrum of **4** ($CDCl_3$, 500 MHz).

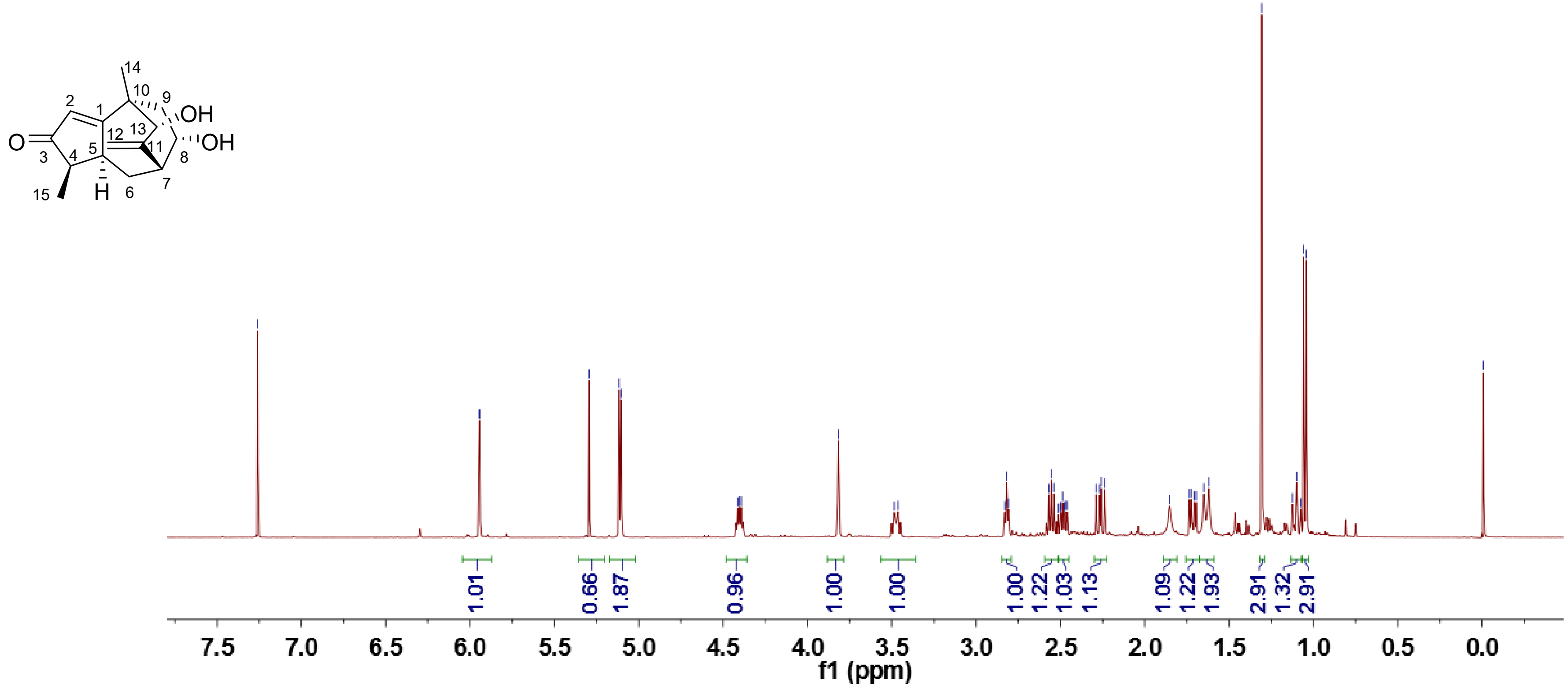

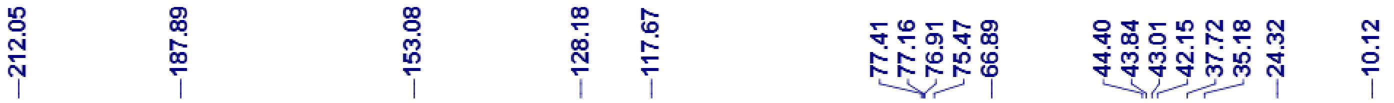

Figure S34. ^{13}C NMR spectrum of **4** ($CDCl_3$, 126 MHz).

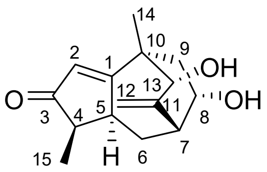

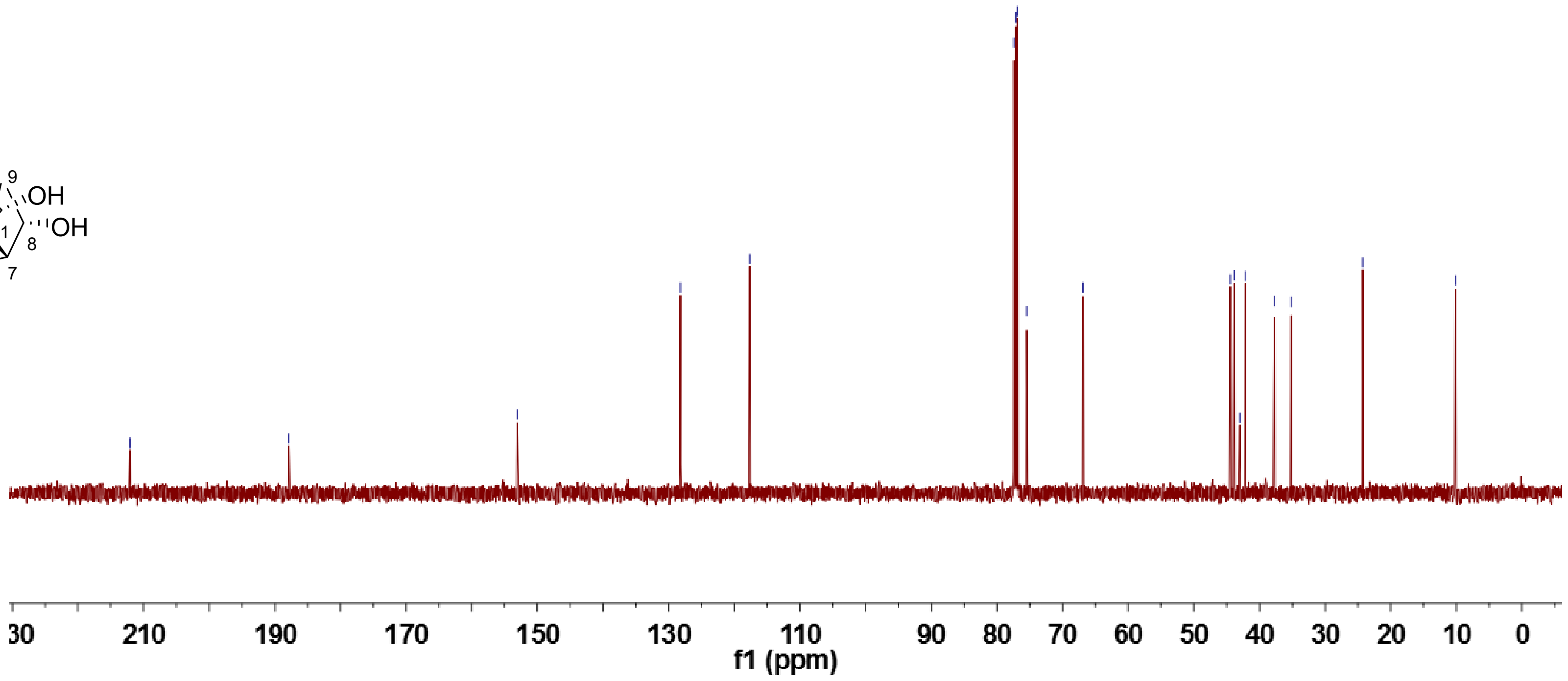

Figure S35. HSQC spectrum of **4** in $CDCl_3$.

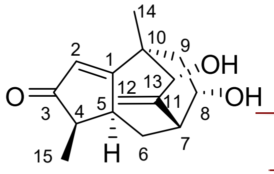

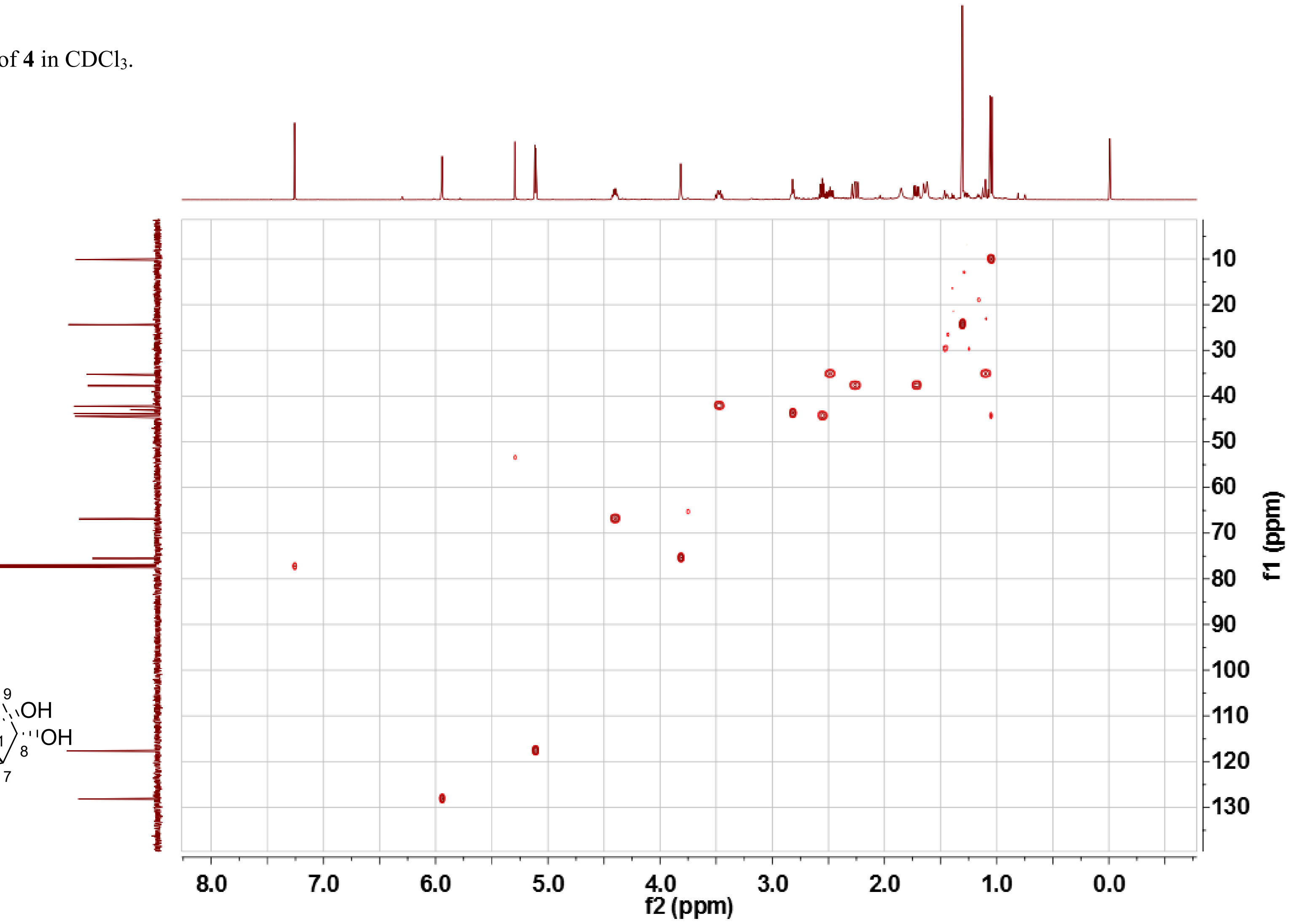

Figure S36. ^{1}H–^{1}H COSY spectrum of **4** in $CDCl_3$.

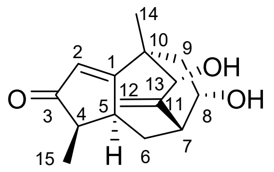

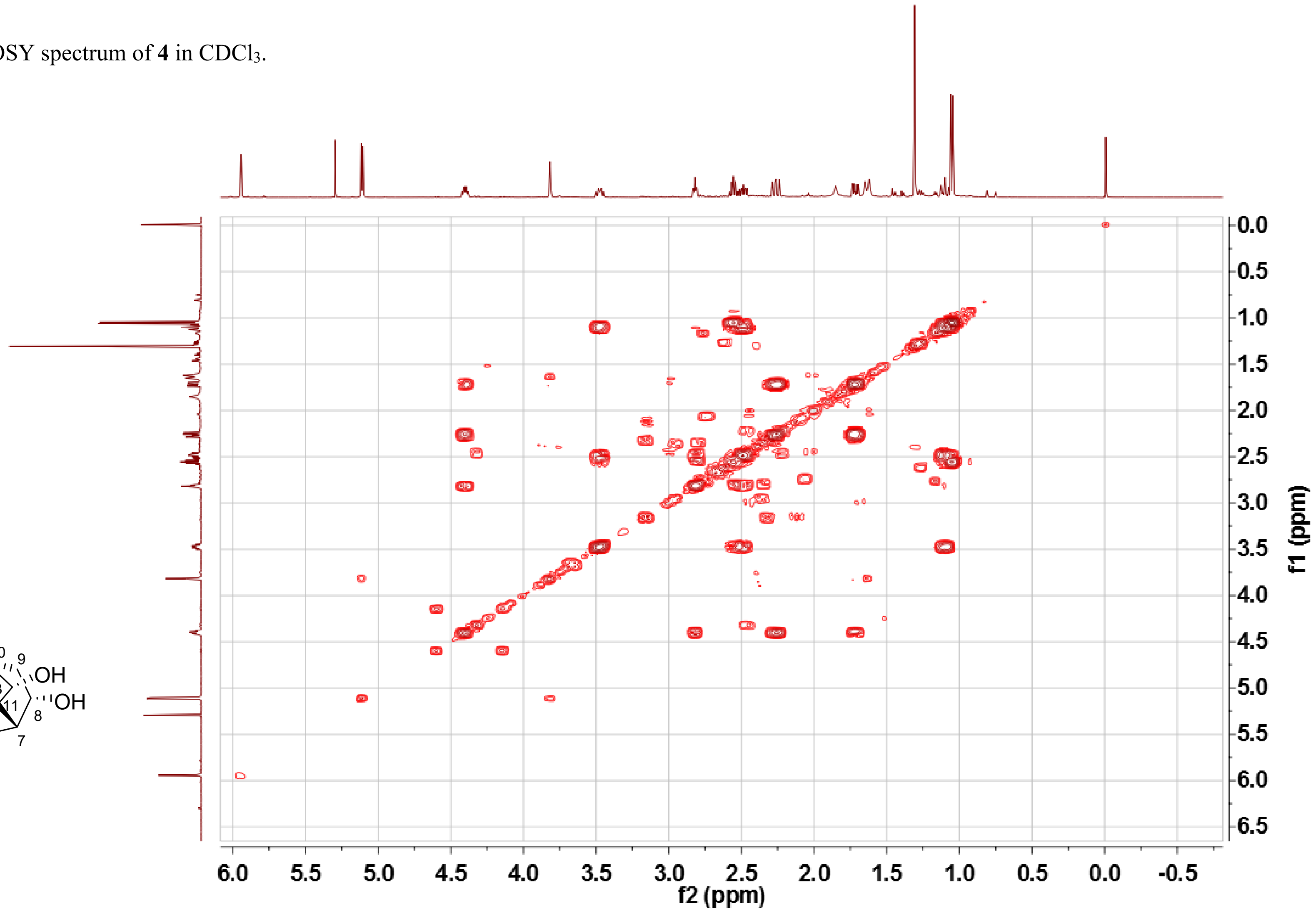

Figure S37. HMBC spectrum of **4** in $CDCl_3$.

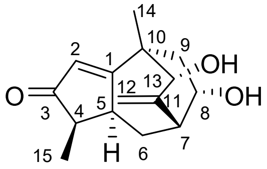

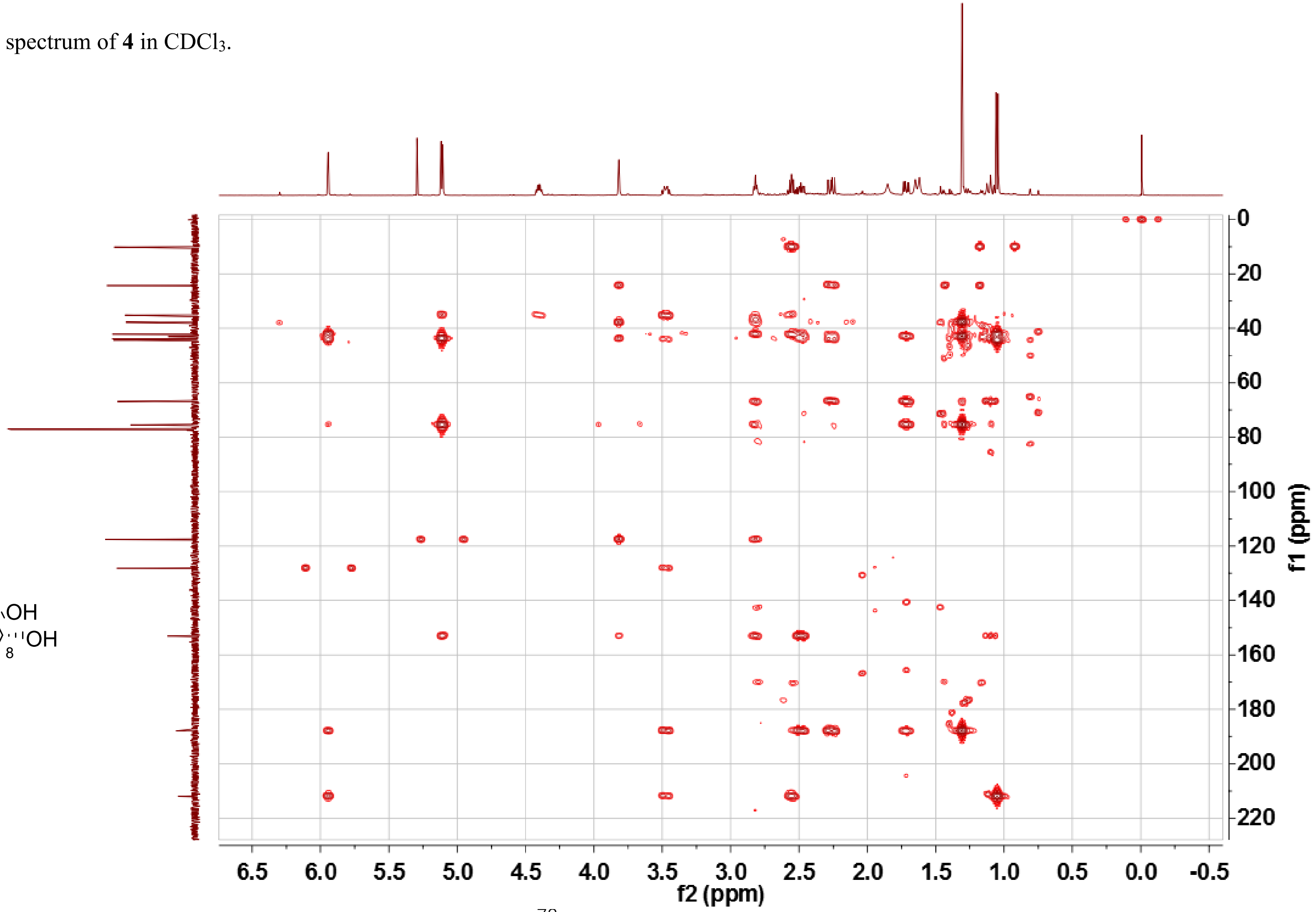

Figure S38. ROESY spectrum of **4** in $CDCl_3$.

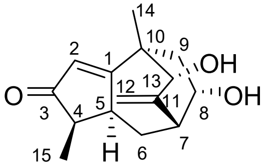

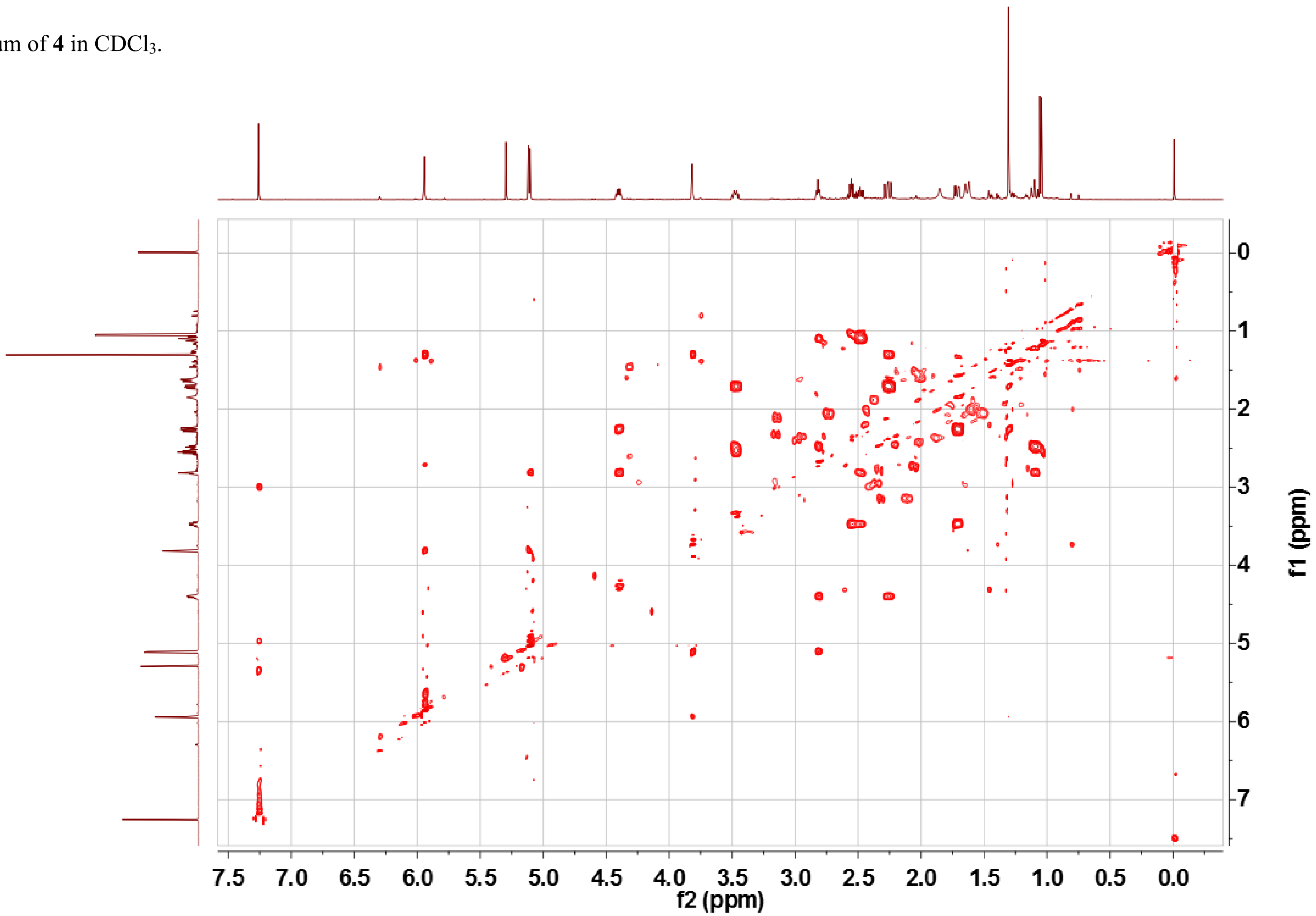

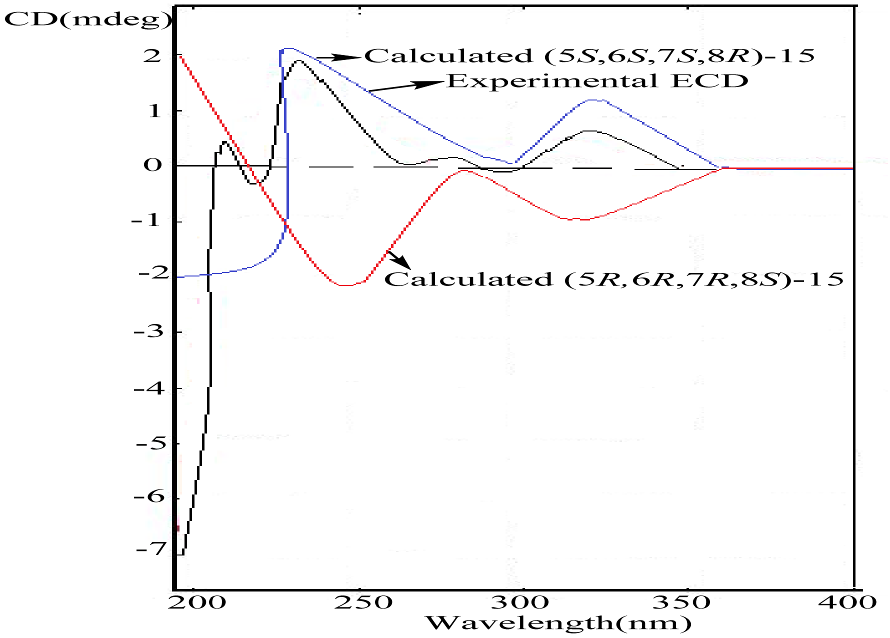

Figure S39. Experimental and computed ECD spectra of **15**.
